# TP53_PROF: a machine learning model to predict impact of missense mutations in *TP53*

**DOI:** 10.1093/bib/bbab524

**Published:** 2022-01-18

**Authors:** Gil Ben-Cohen, Flora Doffe, Michal Devir, Bernard Leroy, Thierry Soussi, Shai Rosenberg

**Affiliations:** Gaffin Center for Neuro-Oncology, Sharett Institute for Oncology, Hadassah Medical Center and Faculty of Medicine, Hebrew University of Jerusalem, Israel; The Wohl Institute for Translational Medicine, Hadassah Medical Center and Faculty of Medicine, Hebrew University of Jerusalem, Israel; INSERM UMR 1186, Integrative Tumor Immunology and Immunotherapy, Gustave Roussy, Faculté de Médecine, Université Paris-Sud, Université Paris-Saclay, 94805 Villejuif, France; Gaffin Center for Neuro-Oncology, Sharett Institute for Oncology, Hadassah Medical Center and Faculty of Medicine, Hebrew University of Jerusalem, Israel; The Wohl Institute for Translational Medicine, Hadassah Medical Center and Faculty of Medicine, Hebrew University of Jerusalem, Israel; Sorbonne Université, UPMC Univ Paris 06, F- 75005 Paris, France; Sorbonne Université, UPMC Univ Paris 06, F- 75005 Paris, France; INSERM, U1138, Centre de Recherche des Cordeliers, Paris, France; Department of Immunology, Genetics and Pathology, Uppsala University, Uppsala, Sweden; Gaffin Center for Neuro-Oncology, Sharett Institute for Oncology, Hadassah Medical Center and Faculty of Medicine, Hebrew University of Jerusalem, Israel; The Wohl Institute for Translational Medicine, Hadassah Medical Center and Faculty of Medicine, Hebrew University of Jerusalem, Israel

**Keywords:** *TP53*, genetic counseling, Li-Fraumeni syndrome, precision medicine, personalized oncology, machine learning

## Abstract

Correctly identifying the true driver mutations in a patient’s tumor is a major challenge in precision oncology. Most efforts address frequent mutations, leaving medium- and low-frequency variants mostly unaddressed. For *TP53*, this identification is crucial for both somatic and germline mutations, with the latter associated with the Li-Fraumeni syndrome (LFS), a multiorgan cancer predisposition. We present TP53_PROF (prediction of functionality), a gene specific machine learning model to predict the functional consequences of every possible missense mutation in *TP53*, integrating human cell- and yeast-based functional assays scores along with computational scores. Variants were labeled for the training set using well-defined criteria of prevalence in four cancer genomics databases. The model’s predictions provided accuracy of 96.5%. They were validated experimentally, and were compared to population data, LFS datasets, ClinVar annotations and to TCGA survival data. Very high accuracy was shown through all methods of validation. TP53_PROF allows accurate classification of *TP53* missense mutations applicable for clinical practice. Our gene specific approach integrated machine learning, highly reliable features and biological knowledge, to create an unprecedented, thoroughly validated and clinically oriented classification model. This approach currently addresses *TP53* mutations and will be applied in the future to other important cancer genes.

## Introduction

Cancer is caused by a sequence of acquired somatic genomic aberrations [1]. Subset of tumors is familial and occurs on a background of germline mutations [2]. Large-scale sequencing studies showed that individual patients have unique mutations profiles, some of which are druggable [3].

This personalized medicine approach already led to major clinical achievements such as in targeting *BRAF* V600E mutations in melanoma [4] and in targeting *EGFR* mutations in lung cancer [5]. It was also shown in a meta-analysis of phase II clinical trials that personalized approach is more beneficial in clinical trials as compared to non-personalized approach [6]. However, application of personalized genomic medicine in cancer is still limited and minority of cancer patients is assigned to this approach [7, 8]. One of the major obstacles is that tumors usually have many mutations, and it is difficult to define the major drivers and accordingly to prioritize drug selections [3]. Two major approaches were proposed and are being used to address this challenge: (i) predicting the consequence of mutations based on biological reasoning such as appearance of mutations in active areas of cancer genes or the occurrence of similar mutations in other cancer patients [3, 9] and (ii) curating literature and knowledge databases for clinical response of tumors with similar mutations to candidate drugs [10, 11]. Nevertheless, these approaches offer limited aid to frequent mutations and not to intermediate frequency and rare mutations. It was estimated that variants in cancer genes in The Cancer Genome Atlas (TCGA) include 88% of variants of unknown significance (VUS) [12]. This limitation can be overcome by the use of computational biology prediction scores for mutations impact using evolutionary conservations and 3D structural considerations. A multitude of in silico predictors aimed at predicting such effects has been proposed but they often do not comprehensively provide similar effects. Furthermore, the relation between the prediction of a deleterious effect in a protein and the pathogenicity is far from being straightforward. Such predictors vary widely by the considerations and score assemblies they integrate, by the type of models they use and by the level of analysis they perform. Consequently, different scores present with different advantages and limitations, but some patterns may be observed. For example, a recent examination of 44 existing in silico tools revealed that, although the majority of tools showed high sensitivity, most were also substantially prone to overcalling deleteriousness, and over two-thirds presented with specificity of under 50% on the measured datasets [13]. In another comprehensive assessment of 33 such predictive algorithms, it was shown that cancer specific tools such as CHASM [14] tend to outperform more general tools [15]. Even so, the top performing algorithms performed more poorly on a set with many low-frequency mutations. The fact that cancer specific algorithms performed better in this analysis strengthens the hypothesis that a simplification of the predictive task is favorable. Focusing the algorithm training method to a gene specific approach is another potential way of simplification. The question of whether such approaches will raise the prediction accuracy is an open question that the work presented below attempts to address.

In comparison to the in-silico tools, functional assays specifically related to the phenotype of the disease should be more accurate, but they are only available for a small number of genes. Among the 27 criteria defined by the American College of Medical Genetics and Genomics (ACMG) to classify disease associated variants, the PS3 criteria (functional studies) has a stronger weight than PP3 (computational prediction) [16]. Whether or not the combination of these two criteria improves variant classification is unknown.

Mutations in *TP53* occur in roughly 42% of tumors [17]. A germline mutation in *TP53* causes the Li-Fraumeni syndrome (LFS) with severe genetic predisposition to cancer [18]. Classification of *TP53* variants from human cancer is highly challenging [19]. Although, the coding sequence of *TP53* is small (1800 nucleotides for a 393 amino acid protein), distinguishing true driver variants from sequencing artifacts, passenger mutations and benign polymorphisms is particularly difficult as missense variants have been found at nearly every *TP53* codons albeit at various frequencies with a high concentration in the 200 residues of the DNA binding domain of the protein [17]. Multiple studies have addressed the loss of function (LOF) of p53 variants using various predictive tools, but results are heterogeneous. In addition, although LOF prediction for hot spot variants and their strong association with a pathogenic score is quite good, it is more heterogenous for infrequent variants [20]. As the clinical relevance of *TP53* diagnostic is increasing for both somatic and germline mutations, there is a dire need for an accurate evaluation of p53 variants LOF [21].

The greatest advantage for the analysis of missense mutations in *TP53* is that the read-out of p53 functions can be easily monitored. In a key paper published in 2003, the group of C. Ishioka published the first large-scale analysis of p53 using a transactivation assay developed in yeast [22]. This functional data, unique for a cancer gene, have been widely used to increase the prediction accuracy of p53 variants [23]. Nevertheless, multiple studies have shown that the relation between the transcriptional activity of p53 variants and the outcome of their biological function such as growth arrest or apoptosis is not straightforward [24]. The two recent large-scale analysis performed in mammalian cells are a new step to increase the accurate identification of p53 variants that sustain a LOF [25, 26]. Taking together these three studies, functional activity of more than 10 000 p53 variants from 12 different readouts are available (see Materials and Methods).

In the present study, we have created TP53_PROF (prediction of functionality), a machine learning model to predict the functional consequences of every possible missense mutation in *TP53*. The model has been validated using multiple independent datasets of normal and cancer patients and it allows a better predictive value for survival analysis.

## Materials and Methods

### Databases

#### The UMD_TP53 database

The 2019_R1 release of UMD_TP53 was used for the present study. It includes the *TP53* status of more than 80 400 tumors, individuals with germline mutations and cell lines analyzed both by conventional Sanger sequencing as well as NGS. A full description and validation of the database in relation with *TP53* mutations from the TCGA has been recently published [27].

*TP53* Mutant Loss of Activity Database, TP53MULTLOAD, first released in 2012 and includes comprehensive details on the properties of p53 variants based on 600 publications. TP53MULTLOAD includes multiple activity fields, such as change of transactivation on various promoters, apoptosis or growth arrest performed in multiple experimental conditions [28]. For several hot spot mutants, multiple gain of function activities are also included. As of today, TP53MULTLOAD includes more than 150 000 entries with multiple entries for most variants. In this manuscript, we used the recommended nomenclature and reference for *TP53* mutations; NM_000546 and NP_000537 for both cDNA and protein variants respectively.

#### Cancer mutation databases

Data from the TCGA and MSKCC studies were downloaded from the cBioPortal (http://www.cbioportal.org/, October 2019). Data from ICGC portal were downloaded from the ICGC website (https://dcc.icgc.org/, data release 26, 17 December 2017). In the first step, minimal genomic data, such as genomic coordinates and genetic events, were extracted from each dataset to define the correct annotation according to HGVS recommendations [29]. In a second step, variant annotation was validated by using the Name Checker tool developed by Mutalyzer (https://mutalyzer.nl/).

TCGA survival data were downloaded from cBioPortal via “TCGA PanCancer Atlas Studies” quick select. Samples with more than one mutation were excluded from the analysis to prevent conflicting conclusions. Patients with more than one sample were also removed to prevent conflict. The rest of the tumors with their *TP53* mutational status and survival information were used (*n* = 10 322). The four categories of *TP53* mutational status, as presented in the results section, each contain: (i) no mutation in the *TP53*—6987 samples, (ii) missense mutation in *TP53* predicted by TP53_PROF to be non-deleterious—58 samples, (iii) missense mutation in *TP53* predicted by TP53_PROF to be deleterious—2126 samples and (iv) tumors with a truncating non-missense mutation in *TP53*—1151 samples. Frameshift deletion or insertion, nonsense, mutations in splice region or in splice site were considered truncating. Tumors with other non-missense mutations in *TP53* were excluded from the analysis.

#### Population database

The Genome Aggregation Database (gnomAD) is a resource developed for aggregating and harmonizing exome and genome sequencing from normal population [30]. It is the largest source of SNP available and includes data from 141 456 individuals. p53 variants were extracted from version 2.1 (version no cancer) and validated by using the Name Checker tool developed by Mutalyzer (https://mutalyzer.nl/).

#### Predictive data

dbNSFP is a database that compiles prediction scores from multiple algorithms, along with conservation scores and other related information, for every potential non-synonymous variant in the human genome [31]. Data for *TP53* were extracted from version 3.5 and manually curated to be specific to the full p53 protein and 21 dbNSFP scores were retained for the analysis (Supplementary Table S8 available online at http://bib.oxfordjournals.org/). Scores originating from seven other in silico predictive softwares were also included in the present study leading to a total of 28 different scores used for the training analysis (Supplementary Table S8 available online at http://bib.oxfordjournals.org/).

#### Functional data

The UMD TP53 database includes three sets of functional data for p53 variants. The first set was described in detail in a previous report [22, 32]. p53 transcriptional activity that is essential for its tumor suppressive function was tested on eight different promoter sequences in a yeast assay. The average and median value of the eight activities were also included as readouts as they can improve the training. The second set of functional data integrated in the UMD_TP53 database corresponds to the analysis of 5300 p53 variants performed by Kotler *et al*. [26]. Cell cycle arrest activity of all variants localized in the DNA-binding domain of p53 was assessed in H1299 cells. The third set corresponds to the study of Giacomelli *et al*. [25]. In this study, dominant negative activity, LOF and response to etoposide were analyzed in mammalian cells for 8258 p53 variants. Taken together, 14 different readouts for p53 function were available. These functional data were removed from TP53MULTLOAD to avoid circular analysis.

Although these three independent studies used different assays, correlation analysis and multidimensional scaling showed excellent agreement between all these variables [33]. In order to avoid any semantic confusion between ‘pathogenicity’ that denote a clinical impact and which is defined by using multiple independent criteria, as recommended by Brnich *et al*., we will use the terminology ‘functionally normal’ or ‘functionally abnormal’ to describe the functional impact of a variant as measured in a given assay [34].

#### The positive and negative training sets

As of yet, training sets used for defining p53 LOF used either the whole set of mutations found in various databases or via the selection of the most frequent p53 variants using an arbitrary cutoff based on the frequency of the variant [9, 35]. Both methods are biased as the first one includes potential passenger and artifactual variants that plagues the various cancer databases and the second does not take into account infrequent non-functional variants. The concept of Cancer Shared Datasets (CSD) was developed to optimize the representativeness of dysfunctional variants in the training set and circumvent the issues described above. *TP53* mutations data were extracted from four non-overlapping databases and variants found at least one time in each dataset were included in the CSD [36]. The four datasets were as follows (i) p53 variants from the UMD database issued from studies that used exclusively conventional Sanger sequencing for the diagnostic; (ii and iii) data from the TCGA and MSKCC studies respectively; (iv) data from ICGC portal. Two hundred and ninety missense variants were found to be shared by the four datasets hereafter named CSD_p53 variants (Supplementary Table S1 available online at http://bib.oxfordjournals.org/). As these four datasets are derived from independent studies using different patients and different methodologies, it is highly likely that these 290 shared variants are true recurrent cancer associated variants. CSD includes both hot spot variants as well as less frequent variants and is more representative of the heterogenous frequency of *TP53* mutations in human cancer [36]. For the negative set, protein variants that were never found (693 variants) or found only once (323 variants) in human cancer have been selected (no_cancer p53 variants) (Supplementary Table S1 available online at http://bib.oxfordjournals.org/). In this analysis, only missense variants have been included as they are the most common alterations detected for *TP53* and the most difficult to classify.

#### Variants for experimental validation

A large-scale analysis of 14 independent genomic databases from the human population led to the identification of multiple missense p53 variants with a minor allele frequency ranging from 10^−6^ to 0.8 [37]. Forty-one of these variants (set 41) were suspected to be either potential non deleterious SNP or pathogenic variants from asymptomatic individuals. These variants were kept aside for experimental validation including seven and five variants from the positive and the negative training datasets, respectively. The criteria used for this selection include variants detected in three or more population datasets at frequency above 5^*^10^−6^ in at least one dataset [37].

#### Scores

For this analysis, 42 different scores were used, and can be divided to two classes: (i) the 28 computational scores and (ii) the 14 functional scores from the three large-scale mutagenesis analysis (Supplementary Table S8 available online at http://bib.oxfordjournals.org/). For scores with missing values, imputation was performed using the median value of each such score.

#### GVS ratio and MMF

The UMD_TP53 database included p53 variants identified in various types of tumors, but in most cases, as normal DNA is usually not available it is possible that rare non-pathogenic SNPs are misclassified as somatic variants. Nevertheless, the large number of variants included in TP53_UMD, allows some specific analysis to identify potential constitutional SNPs. Two criteria have been thus defined, i.e. the germinal to somatic (GVS) ratio and the frequency of multiple mutations (MMF). As the UMD_TP53 database includes both germline and somatic mutations and since the distribution of variants is similar in both, it is possible to define for each variant, the GVS ratio. This will define if a p53 variant is found at higher frequency as germline variants. Similarly, for the MMF score, we investigate the frequency at which each p53 variant is found associated with one or more than one other p53 variant in the same tumor. This score will detect variants that are frequently co-selected because they are either benign passenger variants or low-frequency SNPs. For all variants included in UMD_TP53, the number of p53 variants per tumor has been fully recorded. Although the majority of tumors (91%) express a single p53 variant, 7% and 2% express either two or more than two p53 variants, respectively.

#### LFS analysis

For the LFS validation, two independent datasets were analyzed. The first one is issued from the IARC database and includes 144 families with certified LFS [38]. The second LFS dataset, described by Gao *et al*. [39] was collected from four centers: the MD Anderson [40], The National Cancer Institute (NCI) LFS dataset [41], the Dana Farer Cancer Institute (DFCI) LFS dataset [42] and the Children’s Hospital Of Philadelphia (CHOP) cancer predisposition program. The dataset contains 324 LFS families. The p53 founder variant LRG321t1:c. 1010G > A, (p.R337H) found predominantly in Brazil and included at high frequency in both datasets has been excluded.

#### ClinVar

ClinVar is a public database of variant interpretations that has steadily grown to become the largest publicly available genetic variant database and became a valuable resource to support clinical variant interpretations [43]. ClinVar uses the five tier classification system recommended by the ACMG: Pathogenic (P), Likely pathogenic (LP), Uncertain significance (VUS), Likely benign (LB) or Benign (B) [16]. For the analysis using the confusion matrix, P and LP were grouped and defined as D (deleterious), whereas LB and B were defined as ND (Not deleterious).

As of February 2020, ClinVar included 778 missense variant entries in p53. Thirty variants were removed from the analysis including duplicate variants (22 with similar labels and 8 variants issued for indel). Similar variants with different prediction status or without prediction were defined as VUS. Final analysis was performed on 748 missense p53 variants.

#### Functional analysis

Colony formation assay was performed as previously described [44]. H1299 cells were plated into six-well plates and transfected with various *TP53* constructs. Twenty-four hours after transfection, the cells were dissociated and plated in six-well plates in selective media (G418, 1 mg/ml.) Cells were then stained with crystal violet after 2 weeks.

### In silico tools comparison

The comparison with in silico tools shown in Supplementary Table S7B, available online at http://bib.oxfordjournals.org/, was performed using annotations derived from three experimental assays performed on the set of 41 variants, as described in Supplementary Table S7A available online at http://bib.oxfordjournals.org/. The validation and test set’s labels were taken from the training data. For TP53_PROF’s performance on the validation set, we used the accuracy score obtained during the validation stage, after training only on the training set. ClinVar’s 190 variants with non VUS annotations were considered as another cohort for comparison.

The six tools derived from TP53_UMD were obtained with their classifications. For polyphen, ‘probably damaging’ and ‘possibly damaging’ were considered D. for Mutassessor, ‘low’ and ‘neutral’ were considered ND and ‘medium’ was considered D. Two cutoffs were used for the Revel score, Revel_b (cutoff at 0.5) and Revel_c (cutoff at 0.7), as described in the analysis performed by Cubuk *et al*. [13]. Two thresholds were also used for the CHASM score, CHASM threshold (0.768) and a gene-specific cutoff calculated for *TP53*, CHASM *TP53* truthset threshold (0.892) [14]. For EVE, annotations were used for EVE90pct and EVE75pct, which account for the percentage of certainty. The EVE90pct sets 10% of the variants as ‘uncertain,’ and EVE75pct sets 25% of the variants as ‘uncertain’ [45].

### Data analysis

All data analysis was performed in R, a statistical programming language [46].

The data went through simple pre-processing before transmission to the machine learning models. Preprocessing included replacing non-available (NA) values with the median of all mutations that did have a value for that certain feature.

### Machine learning analysis

Machine learning analysis was performed using two algorithms: Random forests (RF) and gradient boosting machine (GBM). The R Caret [47] package was used to run the machine learning algorithms, to perform hyperparameter tuning, to validate and to test the models. We used a design of 60:20:20 as follows: 60% of the samples used for training and for hyperparameter tuning with 10-fold cross validation. 20% for validation and for choosing the best performing algorithm. The last 20% was reserved for a final test set examination of the model’s performance. Unsupervised multidimensional scaling was done using Euclidean distance.

#### Random forests

We ran the RF algorithm using the ‘rf’ method of the CARET [47] package. ‘RF’ is an ensemble learning method, used in our case for binary classification. This is done by using the bagging technique on decision trees. Bagging means to repeatedly select *n* (a hyperparameter) random samples from the training set, before fitting a decision tree for those samples. In addition to bagging, ‘RF’ also randomly selects a subset of features. The decision tree is thus trained on a subset of samples and on a subset of features from the original data. Each tree is trained as a binary classifier, and the algorithm chooses the class according to a majority tree votes.

#### Gradient boosting machine

The model ran using the ‘xgbTree’ method of the CARET [47] package. GBM is an approach for improving predictions resulting from decision trees. It fits decision trees in sequential order. The first trees are fit to parts of the original data, and trees are than fit to information from previously grown trees. This allows a slow process of improving the model’s residuals. The loss function is slightly improved by each tree, and thus different areas of poor performance can be improved independently [48]. Hyperparameter tuning was performed via random search, i.e. performance was tested under randomly picked hyperparameter values. The random search approach was proven to be better, both empirically and theoretically, than the (non-random) grid search approach [49].

### Survival analysis

Survival plots and statistical distinction analysis were done using the functions Survfit and coxph (both are from the Survival package).

Multivariable analysis was performed with the coxph function in R. Only TCGA samples with missense mutation in *TP53* were included in the analysis. The tumor types PCPG (*n* = 1 samples with missense mutation in *TP53*), TGCT (*n* = 1) and DLBC (*n* = 3) were considered outliers due to extreme survival values coupled with very small sample sizes. They were therefore excluded from this analysis.

## Results

### Data assembly

The open reading frame of the major transcript of *TP53* (NM_000546.6) can sustain 3546 single nucleotide substitutions leading to 2569 different cDNA variants (c-variants) and the synthesis of 2314 potential protein variants (p-variants) (Supplementary Figure S1A available online at http://bib.oxfordjournals.org/). Among the 1750 c-variants (1624 p-variants) that have been described in the 2019 release of UMD_TP53 database, 103 c-variants (102 p-variants) were described in more than 100 cases corresponding to 70% of missense variants detected in human tumors with eight protein variants described more than 1000 times and corresponding to 29% of the patients (Supplementary Figure S1B available online at http://bib.oxfordjournals.org/). On the other hand, 1158 c-variants (1077 p-variants) were described at low frequency (1–9 times) and correspond to 6% of patients in the database. Four hundred eighty nine c-variants (470 p-variants) are found at intermediate frequency (10–99 times) and correspond to 24% of the patients. Although for a few hot spot variants oncogenic activity was profusely validated in multiple cellular or mouse models, information related to less frequent p53 variants is scarce.

Despite the fact that the spectrum of mutation types may vary across tissues, it remains comparable for different tumor types, and missense mutations are consistently predominant. (Supplementary Figure S1C available online at http://bib.oxfordjournals.org/).

**Figure 1:**
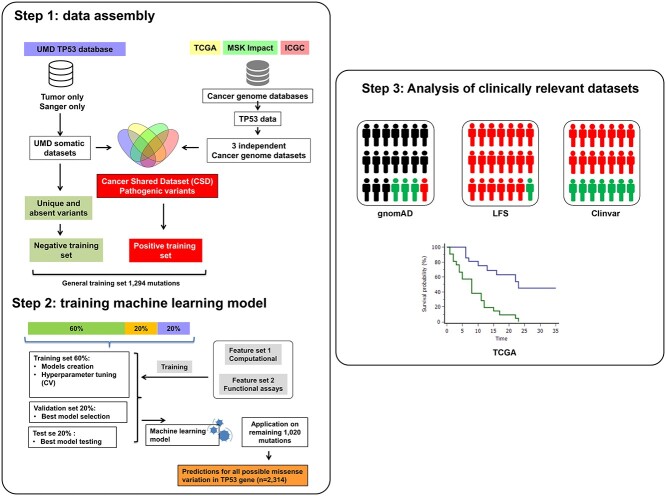
A workflow diagram illustrates the strategy used for the development of the algorithm. Step 1 diagram outlines the various datasets that were used for the development and the validation of the predictor algorithm. It also outlines the analytical outline. Step 2 depicts the training and the validation processes used in this study. Step 3 describes the various datasets used to validate the analysis.

The number of novel missense variants has not increased significantly for several years now [50], indicating that a saturation plateau has been reached with the discovery of all potential p53 variants that sustain a loss of their tumor suppressor functions (Supplementary Figure S2 available online at http://bib.oxfordjournals.org/). Among all different missense p-variants that can be issued from single nucleotide substitutions in the coding region of *TP53*, one third (693 out of 2314, 29.9%) have never been described in human cancer and thus can be considered as non-oncogenic [17]. This assumption is supported by the observation that these variants did not display a LOF in multiple large-scale analyses [33]. This unique situation for a tumor suppressor gene provided the opportunity to define innovative positive and negative training datasets for our analysis. Therefore, the functional part of the training set was defined using rare (described once in only one cancer database) or absent variants whereas the non-functional variants used for the training set were issued from the CSD, that includes p53 variants coexisting in four major independent cancer mutation databases [36] (see Materials and Methods) (Figure 1 and Supplementary Table S1 available online at http://bib.oxfordjournals.org/). The high redundancy of these cancer associated p53 variants in multiple large-scale datasets gave us the opportunity to alleviate all possible bias associated with variants selected according to any specific predefined thresholds. Overall, the negative set contains 1016 variants (1011 after removing variants kept for experimental validation) and the positive set contains 290 variants (283 after removing variants kept for experimental validation). The final curated dataset of 1294 variants, their labels and the 42 possible features is given in Supplementary Table S2 available online at http://bib.oxfordjournals.org/.

### Model development

The training dataset was randomly divided into 60:20:20% groups. Sixty percent of the selected variants for training. Twenty percent of the variants were used for validation, hyperparameter tuning and model selection (Supplementary Table S3 available online at http://bib.oxfordjournals.org/). About 20% were left for final testing. Models were trained using three combinations of feature sets: [1] all features and then separately on the [2] computational and the [3] functional variables. The models were constructed in accordance with the ACMG criteria for p53 variant interpretation, a guideline developed for clinical interpretation of sequence variants for genetic consultation. Under these criteria, functional assays are considered as strong criteria to indicate pathogenicity and computational biology-based scores are considered as moderate ones [16]. Accordingly, three models were created: based on all the features, based solely on functional scores, and based solely on computational scores. The functional model and the computational model each correspond to the type of features used for learning and can fit the respective ACMG criteria. The third model integrates both computational and functional features into one learning model. Training was done using 60% of the training set. Two machine learning algorithms, RF and GBM, were used to analyze the above-mentioned combinations. The accuracy of these different runs was tested on the validation (unused 20%) data.

**Figure 2:**
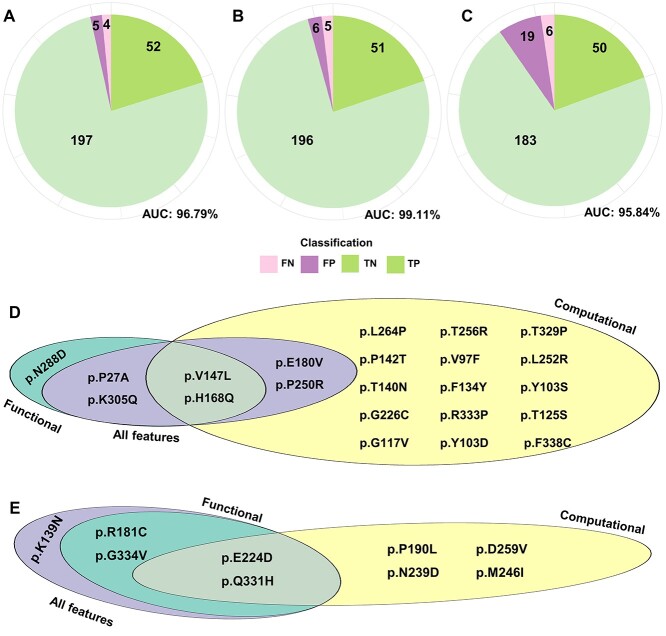
Results and discrepancies on the test set for functional, computational and for all the features models. (**A**) Pie-chart with the performance of the functional model analyzed on the test set. The functional model correctly predicted 249 out of 258 variants in the test set (96.51% accuracy), AUC is 96.79%. Green is for true positive (TP) predictions, light green for true negative (TN), light purple for false negative (FN) and dark purple for false positive (FP). (**B**) Pie-chart similar to (**A**) for all features model. (**C**) Pie-chart similar to (**A**) for the computational model. (**D**) Venn diagram showing discrepancies in the negative set of the three models and their intersections. (**E**) Venn diagram similar to (**D**), for the positive set.

The analysis performed using GBM and RF showed similar performances. GBM performed with 99.66% AUC for both functional and all feature models, and with 97.64% AUC for the computational model (Supplementary Table S4A available online at http://bib.oxfordjournals.org/). RF performed with 99.76% AUC for the functional model, 99.56% AUC for all the features model and with 97.62% for the computational model (Supplementary Table S4B available online at http://bib.oxfordjournals.org/). The computational model performed worse both for RF and GBM. To determine the best model for further testing, 10 tuned runs were performed for each algorithm on both functional and all feature models and the mean AUC was compared (Supplementary Table S4C available online at http://bib.oxfordjournals.org/). The GBM models performed better according to this analysis, by 1.2% for all features model and by 0.02% for the functional model, with the functional model again outperforming the model using all the features. GBM also performed better than RF in terms of accuracy, by 1.17% for all features models and by 3.1% for the functional models (Supplementary Table S4A and B available online at http://bib.oxfordjournals.org/). Hence, at the end of the validation process, the GBM algorithm was chosen over RF, and specifically the functional features GBM model is chosen at the front for deep further analysis. Since the other two models have relevance in matching the different ACMG criteria, they were further tested as well, as presented below. For the final step, each model was trained on 80% of the data (train and validation sets combined) and tested on the test set of 20% that was kept aside for this purpose. As shown in Figure 2, the functional features model had an AUC of 96.8% and an accuracy of 96.5% (Figure 2A, ROC curve in Supplementary Figure S3A available online at http://bib.oxfordjournals.org/). The sensitivity was 92.8% and specificity was 97.5%. The performance of the other two algorithms is presented in Figure 2B and C, ROC curves in Supplementary Figure S3B and C available online at http://bib.oxfordjournals.org/. A table of the 2314 missense variants with their TP53_PROF functional model predictions is provided in Supplementary Table S5 available online at http://bib.oxfordjournals.org/. Variable importance scores of the functional features for the functional model’s predictions were calculated, indicating that features from the Giacomelli (0.44) and Kotler (0.36) papers were most dominant in the model’s predictions process (Supplementary Table S6 available online at http://bib.oxfordjournals.org/).

We examined the discrepancy variants issued from the algorithm’s predictions on the test set (Figure 2D and E). The best algorithm (based on functional features) predicted four variants to be functionally active although they are included in the non-functional training set. A close examination of these four variants shows that they are indeed functionally impaired cancer associated variants (Supplementary document TP53 variants update). Two of these variants (p.E224D and p.Q331H) are localized at the vicinity of exon/intron junction sequences and are well known to be associated with dysfunctional splicing and nonsense-mediated mRNA decay [33, 51]. As all functional scores are issued from experimental data based on forced expression of protein variants, functional data as well as in silico protein function predictors used for these null variants will not be accurate thus causing false negative scores. The third variant is localized in codon 181 (p.R181C) that have been shown to be essential with codon 180 for dimer stability [52, 53]. Variants at codon 181 such as p.R181C or p.R181H do not fully abolish p53 function and have differential LOF depending on the *TP53* target genes (see also experimental validation in the next section and Supplementary document TP53 variants update). Germline variants of the form p.R181C were found to be a founder mutation associated with an increased risk of breast cancer in Arab families and is the only p53 variant that can be identified in a situation of homozygosity, suggesting a low penetrance associated with a partial loss of the tumor suppressive function [54]. The fourth variant, p.G334V, is located in the tetramerization domain and is well known to disrupt p53 oligomerization [55–57]. Variants in this domain are difficult to interrogate in cellular assay as artificial overexpression alleviate this defect with forced oligomerization and a potent activity (see next section). Taken together, these four false negative variants are included in the CSD and are indeed non-functional but cannot be accurately assessed via the current algorithms. On the other hand, five variants labeled as functional (never detected in human cancer) were predicted to be functionally impaired. Although multiple explanations can be considered such as no sufficient loss of activity to impair the tumor suppressive effect of p53, it is also possible that these variants are counter selected in normal cells due to a toxic effect.

### Similarity between the training set and the whole mutations space

**Figure 3:**
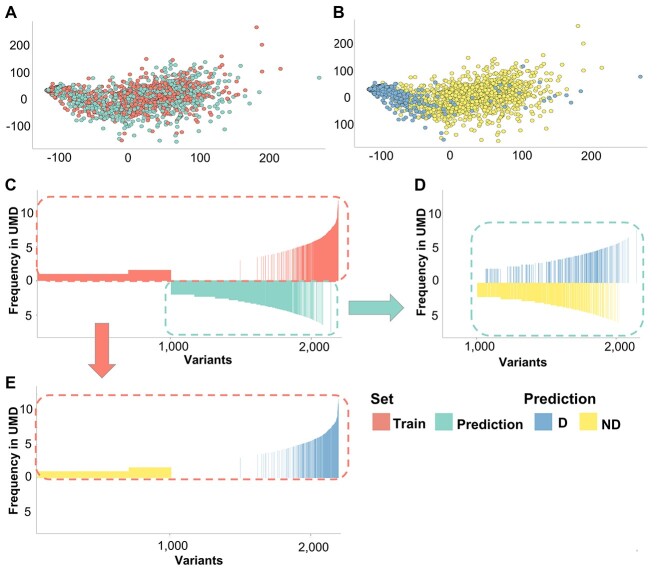
Bidimensional MDS. Euclidean distance between each pairwise variants of the UMD database was calculated using all variant features. (**A**) MDS discerning the variants in the training set (orange) and the rest of the variants predicted by TP53_PROF (blue). The two groups disperse evenly in the variants space. (**B**) MDS discerning deleterious (blue) versus non-deleterious (yellow) variants. (**C**) Variant frequency in UMD of the variants predicted by the algorithm. Variants used for training are in red and the variants from the prediction set are in turquoise. *X*-axis: p53 variants ranked according to their frequency in UMD from left to right. *Y*-axis: frequency of each variant in UMD (Log2 scale). (**D**) Similar to (**C**), with variants from the prediction set only. Variants are colored by their TP53_PROF prediction: Deleterious variants in yellow, non-deleterious variants in blue. The deleterious and non-deleterious variants in this set present with a mixed frequency in UMD. (**E**) Similar to (**D**), with variants from the training set only.

The algorithm for TP53_PROF was trained on selected variants rather than on randomly chosen ones, since it is possible to label only a subset of variants as functional or non-functional. To minimize biases, we predefined general and simple rules for the inclusion of variants in the training data sets as mentioned above and as described in Materials and Methods. Using a training set that was taken from the 1294 labeled variants, rather than chosen randomly from all the 2314 variants may result in a biased algorithm that cannot predict accurately the remaining unlabeled 1020 variants from the prediction set. Hence, it is important to verify that the training set is not distinct from the prediction set in its properties. Accordingly, a dimensionality reduction algorithm was applied to examine whether the training variants are dispersed evenly in the features space with all the rest of the variants and thus represent the whole mutational landscape. Multidimensional scaling (MDS) reduced the 42 features into a two-dimensional space and was performed on variants used for the training as well as on the rest of variants from UMD_TP53 database. Variants used for training and the rest of the variants were presented with even distributions on the two MDS axes, supporting the assumption that these variants represent well the mutational landscape of *TP53* (Figure 3A). On the other hand, as expected, functionally inactive variants are more centered compared to active variants (Figure 3B). Moreover, these findings allow us to group the labeling of the training set and the algorithm’s prediction of the prediction set into a unified approach to predict pathogenicity to all 2314 missense variants of *TP53*. In the coming sections, we show the validation of this unified approach.

Figure 3C–E shows the algorithm’s predictions as related to variants frequency in the UMD_TP53 database. As expected, non-functional variants tend to have higher frequency compared to functional variants. Nevertheless, the two groups cannot be separated accurately using frequency information alone—thus highlighting the importance of the predictive algorithm.

### SNP prediction and experimental validation

The 41 p53 variants (set 41) that were kept aside from the training for experimental purposes, include p53 variants found in population databases and suspected to be either potential benign SNP or pathogenic variants from asymptomatic individuals [37] (see Materials and Methods). Among this set, the 15 variants that were recently validated as *bona fide* SNPs [37], including the two most common polymorphisms p.P72R and p.P47S, were classified as functional by TP53_PROF (Table 1 and Supplementary Table S7A available online at http://bib.oxfordjournals.org/). None of these variants have been described as dysfunctional either in the three large-scale functional datasets or in the MUTLOAD database (Supplementary Figure S4A and B available online at http://bib.oxfordjournals.org/). Furthermore, their frequency of multiple mutations (MMF) and germinal to somatic (GVS) scores (see Materials and Methods) show that they were outliers found at high frequency as germline variants or associated with other p53 variants in human tumors (Supplementary Figure S5A and Supplementary Table S7A available online at http://bib.oxfordjournals.org/). Among the remaining 20 variants also classified as functional, nine variants are likely SNPs found at low frequency in the human population, 10 cannot be classified precisely and the remaining one is a well-known passenger variant (p.R175C) [37]. None of these variants display any loss of activity in the three large-scale functional datasets and have high GVS and/or MMF scores (Supplementary Figure S4A and B and Supplementary Table S7A available online at http://bib.oxfordjournals.org/). Finally, the six variants that were defined as non-functional by TP53_PROF include five *bona fide* cancer associated variants found in multiple cancer patients (Table 1 and Supplementary Table S7A available online at http://bib.oxfordjournals.org/).

**Table 1:** Predictions and functional analysis of p53 variants from set 41

TP53 variant	TP53_PROF	Growth arrest (present work)	Biological activity [37]	Activity (Mutload)	Clinvar (October 2020)	UMD (Count)	gnomAD AC/AF	Classification [37]
TP53 SNP								
c.31G > C_p.E11Q	Functional	Active	Active	Active	VUS	71	14/0.000049	Benign (BA1) SNP^b^
c.91G > A_p.V31I	Functional	Active	Active	Active	VUS	77	66/0.000235	Benign (BA1) SNP^b^
c.139C > T_p.P47S	Functional	Active	Active	Active	B	0	450/0.0016	Benign (BA1) SNP^a^
c.173C > G_p.P58R	Functional	Active	Active	Active	VUS	4	27/0.00009	Benign (BS1 + BS3) SNP^b^
c.215C > G_p.P72R	Functional	Active	Active	Active	B	0	186 832/0.663	Benign (BA1) SNP^a^
c.319 T > C_p.Y107H	Functional	Active	Active	Active	B	18	34/0.00012	Benign (BA1) SNP^b^
c.329G > A_p.R110H	Functional	Active	Active	Active	LB	41	13/0.000046	Likely benign (BS3 + BP4) SNP^b^
c.566C > T_p.A189V	Functional	Active	Active	Active	VUS	53	6/0.000024	Benign (BA1) SNP^b^
c.704A > G_p.N235S	Functional	Active	Active	Active	B	80	55/0.0002	Benign (BA1) SNP^b^
c.847C > T_p.R283C	Functional	Active	Active	Active	VUS	157	21/0.000074	Benign (BS1 + BS3) SNP^b^
c.869G > A_p.R290H	Functional	Active	Active	Active	B	118	43/0.00015	Benign (BS1 + BS3) SNP^b^
c.935C > G_p.T312S	Functional	Active	Active	Active	B	9	21/0.000074	Benign (BS1 + BS3) SNP^b^
c.1015G > A_p.E339K	Functional	Active	Active	Active	VUS	21	18/0.0000638	Benign (BS1 + BS3) SNP^b^
c.1073A > T_p.E358V	Functional	Active	Active	Active	VUS	17	5/0.00002	Benign (BA1) SNP^b^
c.1079G > C_p.G360A	Functional	Active	Active	Active	LB	25	54/0.0002	Benign (BS1 + BS3) SNP^b^
Functional TP53 variants								
c.188C > G_p.A63G	Functional	Not done	Active	Active	VUS	2	1/0.000032	No classification
c.214C > G_p.P72A	Functional	Not done	Active	Active	VUS	32	4/0.0000142	VUS (rare SNP?)
c.248C > T_p.A83V	Functional	Active	Active	Active	VUS	12	35/0.00014	VUS (rare SNP?)
c.374C > T_p.T125M^c^	Functional	Not done	Not done	Partial	VUS	124	1/0.000032	No classification
c.466C > T_p.R156C	Functional	Not done	Active	Active	VUS	49	1/0.000004	Likely benign (BS3 + BP4)
c.523C > T_p.R175C	Functional	Active	Active	Active	VUS	109	5/0.000017	Passenger mutation
c.554G > A_p.S185N	Functional	Active	Active	Active	LB	7	1/0.000004	Likely benign (BS3 + BP4)
c.558 T > A_p.D186E	Functional	Not done	Active	Active	VUS	5	1/0.000004	No classification
c.642 T > G_p.H214Q	Functional	Not done	Active	Active	VUS	8	8/0.000032	VUS (rare SNP?)
c.665C > T_p.P222L	Functional	Active	Active	Active	VUS	36	6/0.00002	VUS (rare SNP?)
c.760A > G_p.I254V	Functional	Active	Active	Active	VUS	24	4/0.000016	VUS (rare SNP?)
c.877G > T_p.G293W	Functional	Not done	Active	Active	LB	20	3/ 0.00001	No classification
c.884C > T_p.P295L	Functional	Not done	Active	Active	VUS	18	6/0.000021	VUS (rare SNP?)
c.949C > A_p.Q317K	Functional	Not done	Active	Active	VUS	13	2/0.000007	No classification
c.998G > A_p.R333H	Functional	Not done	Active	Active	VUS	16	12/0.000042	VUS (rare SNP?)
c.1025G > A_p.R342Q	Functional	Not done	Active	Active	VUS	14	2/0.000008	No classification
c.1061A > G_p.Q354R	Functional	Not done	Active	Active	VUS	7	2/0.000008	No classification
c.1096 T > G_p.S366A	Functional	Active	Active	Active	LB	14	15/0.00006	VUS (rare SNP?)
c.1120G > C_p.G374R	Functional	Not done	Active	Active	LB	3	2/0.000007	No classification
c.1129A > C_p.T377P	Functional	Active	Active	Active	No data	11	71/0.00025	VUS (rare SNP?)
Non-functional TP53 variants								
c.460G > A_p.G154S^d^	Non-functional	Not done	Inactive	Partial	VUS	36	6/0.000021	No classification
c.467G > A_p.R156H	Non-functional	Not done	Inactive	Inactive	VUS	70	4/0.000016	Pathogenic
c.542G > A_p.R181H	Non-functional	Inactive	Inactive	Inactive	P	133	4/0.000014	Pathogenic
c.713G > A_p.C238Y	Non-functional	Not done	Inactive	Inactive	P/LP	457	2/0.000008	Pathogenic
c.743G > A_p.R248Q	Non-functional	Not done	Inactive	Inactive	P	3963	3/0.000012	Pathogenic
c.848G > A_p.R283H^d^	Non-functional	Not done	Active	Partial	VUS	79	10/0.00004	No classification

*Note*: TP53_PROF: Functional or non-functional variants as defined by the present study. Activity measures are given in three different columns: Growth arrest as defined in the present study; Biological activity as defined by [37]; Functional activity issued from mutload. ClinVar: annotations given in ClinVar. UMD (Count): number of occurrences in UMD. gnomAD AC/AF: Allele count (AC) and allele frequency (AF) in gnomAD.

^a^Variants previously defined as *bona fide TP53* SNPs.

^b^ *TP53* variants recently defined as benign or likely benign SNPs.

^c^p53 variants at codon 125 are well known to impair *TP53* splicing precluding protein expression. Forced artificial expression of these variants will not be evidence of functionality.

^d^Literature regarding these variants is controversial with no definitive classification but this ambiguity is usually associated with a partial defect (see Supplementary Table S7A, available online at http://bib.oxfordjournals.org/ for more information).

**Figure 4:**
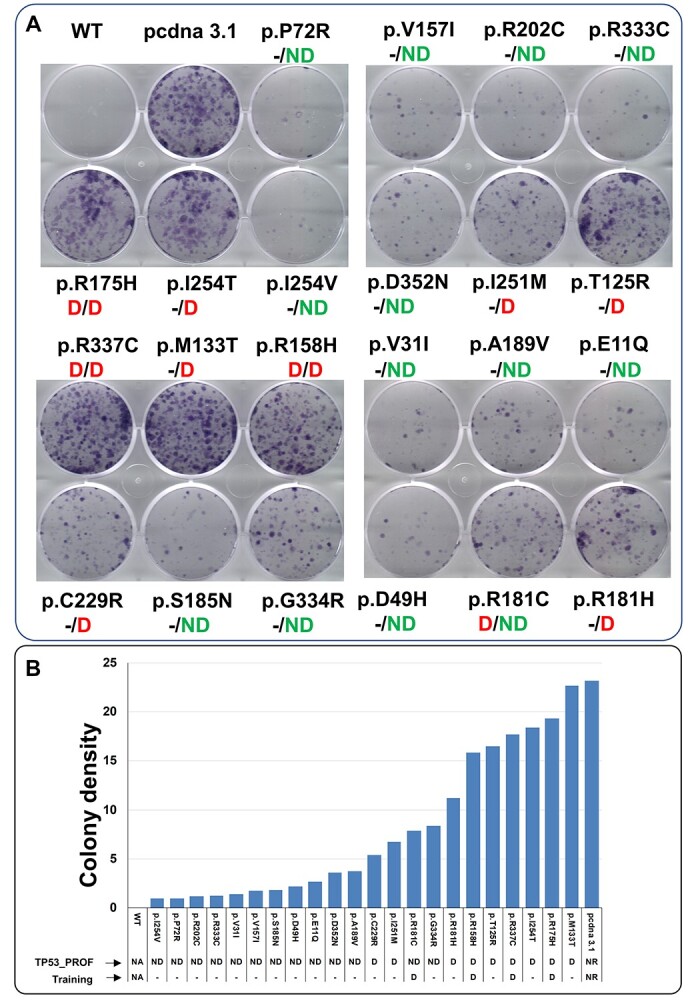
Functional analysis of p53 variants. (**A**) Representative photos of colony formation assay. Plates were stained two weeks after transfection. Wild type p53 as well as several p53 variants can inhibit colony formation whereas the cancer associated variant, p.R175H, used as a positive control, does not inhibit colony formation. Inclusion of p53 variants in the training or in the predictive set is shown under the name of the variant separated by a backslash. ‘D’, ‘ND’ or ‘-‘: deleterious, non-deleterious or not included. For variants in the test set (20% of the training set) both labeling and prediction are given. (**B**) Quantitative summary of the colony formation assay in (**A**). Inclusion of p53 variants in the training or in the predictive set is shown under the name of the variant.

Growth arrest activity of p53 variants confirmed the prediction of TP53_PROF (Figure 4, Table 1). As expected, the variant at position p.R181C have an intermediated phenotype (see Supplementary document TP53 variants update). Variant, p.G334R, located in the tetramerization domain shows a partial defect in our functional assay but display an intact activity in all published multiple large-scale studies, stressing the difficulty to analyze variants in this particular domain (Supplementary document TP53 variants update). Nevertheless, this variant was recently shown to be a pathogenic, Ashkenazi Jewish—predominant mutation associated with a familial multiple cancer syndrome, although the LOF was partial [58]. Taken together, analysis of p53 variants from this set of 41 variants, as well as the experimental data, are in good agreement with the predictions of TP53_PROF.

The set of 41 variants was also used to compare our model with other available scores. This set is enriched with non-deleterious variants, thus allowing for the examination of specificity performances, where most current models perform poorly. TP53_PROF’s classification was compared with six scores that provided formal classification cutoff in the TP53_UMD database (Polyphen2 HumVar, Polyphen2 HumDiv, Sift, Condel, Provean and Mutassessor). EVE, a recently released deep generative model of evolutionary data [45], CHASM, a cancer-specific algorithm that showed peak performances in recent analysis [14, 15] and Revel, another in-silico tool that presented with best balanced accuracy in a recently published algorithms comparison [13, 59], were also used for this analysis with different defined cutoffs for LOF provided by the scores documentation (see Methods). Annotations based on the three experimental validations done on the set of 41 variants (presented in Supplementary Table S7A available online at http://bib.oxfordjournals.org/) were used as the truth set. TP53_PROF outperformed the other scores, with accuracy ranging between 97.5% and 100% (Supplementary Table S7B available online at http://bib.oxfordjournals.org/). The second-best score was EVE75pct (92.6–100% accuracy). However, EVE75pct defines 25% of its predictions as ‘uncertain,’ and therefore had uncertainty regarding 34% [14] of the 41 variants set. EVE90, which defines 10% of its predictions as ‘uncertain,’ had accuracy ranging between 71.1% and 84.8%.

For further comparison, the scores were also compared to the performance of TP53_PROF on the validation and test sets (*n* = 258 each, also enriched for non-deleterious variants) and on ClinVar’s non-VUS variants set (*n* = 190, enriched for deleterious variants). On both the validation and the test sets, TP53_PROF maintained its advantage with 98.06% and 96.5% accuracy scores, respectively. For the validation set, Eve75pct performed second best with 94.6%, but lacked predictions for the significant portion of 78% (202) of the variants. For the test set, CHASM_TP53 (a gene-specific suggested cutoff for CHASM) was second best with 91.4% accuracy, serving as another indication for the utility of the gene-specific approach.

On the ClinVar set, which is highly enriched with deleterious variants, TP53_PROF maintained best performance of 96.3% accuracy, with SIFT and Revel_b presenting with a similar score. The Eve75pct score had an accuracy of 98.03%. However, EVE75pct had predictions for only a subset of 80.5% (153) of ClinVar’s variants. The rest of the scores also performed very well on ClinVar (accuracy ranging between 86.6% and 95.9%). This emphasizes the fact that most models perform well on classifying deleterious variants but not as good in the classification of non-deleterious variants [13]. TP53_PROF by contrast performs well for both deleterious and non-deleterious variants.

### Testing the model on population data

**Figure 5:**
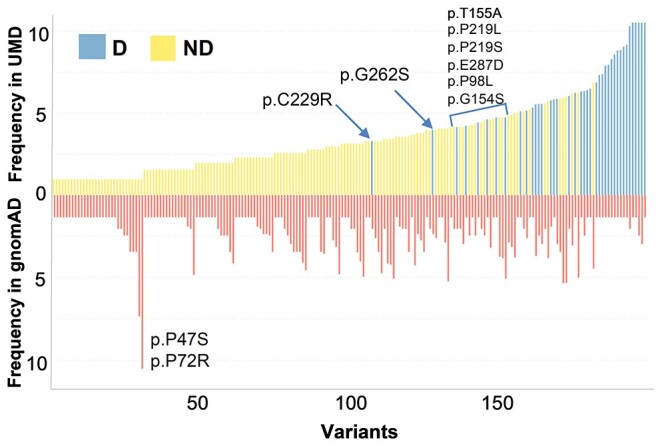
Frequency of 196 variants in gnomAD and UMD databases. Bars showing frequency in gnomAD are colored in red. Bars showing frequency in UMD are colored by the model’s prediction, Blue for deleterious and yellow for non-deleterious variants. Frequency is represented in log2 scale.

We first applied the TP53_PROF on gnomAD, the largest set of genetic variations found in the human population. Although gnomAD includes mostly benign variants, recent studies indicate that it also contains pathogenic variants in tumor suppressor genes such as BRCA1 or TP53 [36, 60]. Indeed, 39 out of the 196 missense p53 variants included in gnomAD have been classified as deleterious by the algorithm, including 22 CSD variants (Figure 5 and Supplementary Figure S7 available online at http://bib.oxfordjournals.org/). They are identified both in the complete database as well as the no-cancer versions of gnomAD, indicating that they are associated with asymptomatic individuals carrying pathogenic p53 variants (Figure 5 and Supplementary Figure S8 available online at http://bib.oxfordjournals.org/). This high frequency is in accordance with the elevated (15–30%) number of *TP53 de novo* mutations in the early onset cancer patients not associated with familial history [61, 62]. On the other hand, 157/196 variants were defined as non-deleterious, including the 15 *TP53* SNPs described above that were recently validated as *bona fide* SNPs [37] (Supplementary Figures S9 and S10 available online at http://bib.oxfordjournals.org/). Although these include the three pathogenic variants described above (splice variants p.E224D and p.T125M and the Brazilian variant p.R337H, known to be associated with adrenocortical carcinoma and whose loss of activity have been difficult to appraise), the remaining variants are found at very low frequency both in gnomAD and UMD and are likely very infrequent private SNPs or sequencing errors (Supplementary Figure S10 available online at http://bib.oxfordjournals.org/).

### Testing the model on data of Li-Fraumeni syndrome patients

Another line of validation was done using variants taken from datasets of LFS patients that should include only pathogenic non-functional variants [39]. First, we used the 144 families (60 different p53 variants) included in the LFS dataset from the IARC database [38]. Two variants were miss-identified SNPs (p.R290H and p.N235S) [37], indeed predicted to be functional by the algorithm. Fifty-four variants were classified as non-functional including 24 variants that were not included in the training set and are from the prediction set (Supplementary Figure S11A available online at http://bib.oxfordjournals.org/). The four remaining variants predicted as functional were low-frequency variants without any obvious loss of activity. Overall, this led to a model prediction accuracy of 93.1% (54/58) after removing the two miss-identified SNPs.

The second dataset described by Gao *et al*. includes 77 p53 variants issued from 324 LFS families. Seventy-one variants were predicted to be deleterious (63 from the training set and eight from the prediction set) (Supplementary Figure S11B available online at http://bib.oxfordjournals.org/). Among the six remaining variants predicted to be functional, three were miss-identified SNP (p.I254V, p.R283C and p.N235S) [11], two are pathogenic variants localized in the tetramerization domain of p53 and the remaining one is the pathogenic variant p.R181C discussed in the previous section (model prediction accuracy is 95.9% after removing the three SNPs).

### Testing the model on ClinVar database

The ClinVar database that is extensively used in genetic testing programs includes 748 p53 missense variants that were classified using the five-tier classification system (pathogenic, likely pathogenic, uncertain significance, likely benign, or benign) [63]. First, matching ClinVar with the two training sets shows that the negative set includes only variants classified as benign (B), likely benign (LB) or VUS, whereas the positive set includes only variants defined as pathogenic (P), likely pathogenic (LP) or VUS, thus supporting our labeling selection procedure (Supplementary Figure S12 available online at http://bib.oxfordjournals.org/).

**Figure 6:**
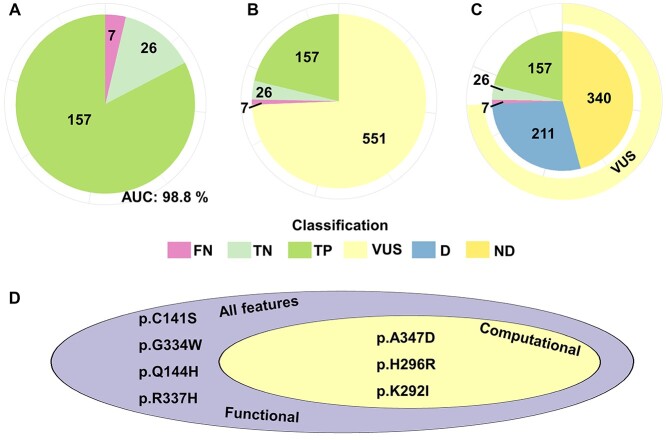
ClinVar comparison with TP53_PROF’s predictions. (**A**) Variants annotated by ClinVar as benign (B), likely benign (LB), pathogenic (P) or likely pathogenic (LP) are compared with TP53_PROF’s deleterious (D) and non-deleterious (ND) predictions. 26 variants were predicted as B or LB by ClinVar and as ND by TP53_PROF and are considered true negative (TN), given in light green. 157 variants were predicted as P or LP by ClinVar and as D by TP53_PROF and are considered TP, given in green. Seven variants were predicted as P or LP by ClinVar and as ND by TP53_PROF and are considered false negative (FN), given in purple. All B or LB variants were predicted ND by TP53_PROF, with no false positives. (**B**) same as (**A**), with the addition of 551 variants annotated by ClinVar as VUS, given in bright yellow. The VUS variants make up 74.3% of the variants in the analysis. (**C**) same as (**B**), with VUS variants (shown by the bright yellow outer circle) also colored by their TP53_PROF prediction. 211 VUS variants were predicted as D, given in blue. 340 VUS variants were predicted as ND, given in yellow. (**D**) Venn diagram showing discrepancies in the TP53_PROF- ClinVar comparison. The discrepancies were similar for the functional and all-features models. The computational model showed better performance in this analysis, with four of the seven false negatives correctly classified, and a remaining discrepancy of three variants.

TP53_PROF predicts that the 26 B or LB variants included in ClinVar are functional (True negative 100%, no false positive) (Figure 6 and Supplementary Figure S13 available online at http://bib.oxfordjournals.org/). On the other hand, among the 164 P or LP variants, 157 are predicted to be non-functional (Supplementary Figure S13 available online at http://bib.oxfordjournals.org/). Hence, the functional model’s predictions are 95.73% sensitive, 100% specific, the accuracy is 96.32% and the area under the curve (AUC) is 98.8%. Among the seven false negative variants, three were localized in the tetramerization domain of the protein (Supplementary Figure S13 available online at http://bib.oxfordjournals.org/).

Five hundred fifty-one ClinVar variants are annotated as VUS, 223 (40%) of which were defined as non-functional by our model. Although, the VUS status suggests an unresolved issue for these variants, the observation that 68 (30%) of them have been described as somatic variants in more than 100 independent studies is highly suggestive of a pathogenic class (Figure 6C).

### Testing the model on survival information

To test TP53_PROF predictions on survival data, we used pan-cancer tumor samples from TCGA. We divided the samples into four categories of *TP53* mutational status: (i) no mutation in *TP53* (*n* = 6987), (ii) missense mutation in *TP53* predicted by TP53_PROF to be functional (*n* = 58), (iii) missense mutation in *TP53* predicted by TP53_PROF to be non-functional (*n* = 2126), (iv) tumors with a truncating non-missense mutation in *TP53* (*n* = 1151).

**Figure 7:**
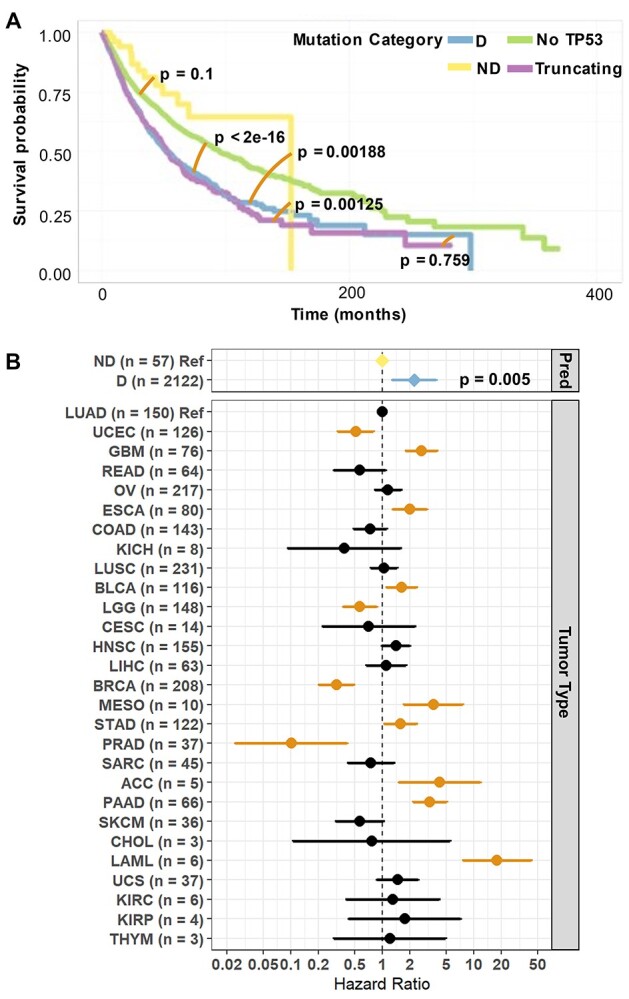
(**A**) Survival curve of tumors from TCGA database. TCGA tumor samples are presented in four groups, by their *TP53* mutational status. Green: Samples with no *TP53* mutation (No *TP53*, *n* = 6987). Yellow: Missense *TP53* mutations predicted by TP53_PROF to be non-deleterious (ND, *n* = 58). Blue: Missense *TP53* mutations predicted by TP53_PROF to be deleterious (D, *n* = 2126). Purple: Samples with a truncating *TP53* mutation (truncating, *n* = 1151). *P*-values for the comparison between these groups are also shown, with an orange line indicating the two groups being compared. Survival predictions are distinct when comparing No *TP53* and D (*P* < 2e-16), comparing ND and D (*P* = 0.00188) and comparing ND and truncating (*P* = 0.00125). Survival predictions are indistinctive for comparisons between D and truncating (*P* = 0.759) and between ND and No *TP53* (*P* = 0.1). (**B**) Multivariable analysis for the cox regression survival model with TP53_PROF’s predictions (pred) and tumor type as variables. The survival distinction of the patient groups D compared to ND predictions, was significant at the *P* = 0.005, independent of the effect of tumor type. Tumor types that had significant effect on the hazard ratio are colored in orange.

Patients with *TP53* mutations predicted to be functional had longer survival times compared to patients with *TP53* mutations predicted to be non-functional (*P* = 0.00188) and compared to patients with truncating mutations (*P* = 0.00125) (Figure 7A). Patients with *TP53* mutations predicted to be non-functional by the algorithm had significantly shorter survival time as compared to patients with no *TP53* mutations (*P* < 2e-16). There was no significant survival difference between patients with functional mutations and patients with no *TP53* mutations (*P* = 0.109), nor between patients with non-functional mutations and patients with known truncating mutations (*P* = 0.759). Interestingly, minority of mutations in the somatic database of TCGA were predicted to be functional (58/3335, 1.74%) and this seems to reflect that such mutations are not positively selected in cancer. These findings provide an independent validation that the algorithm’s prediction has accurate clinical implication. Similar analysis performed for the other models (based on computation features and based on all features) provided comparable results as shown in Supplementary Figure S14A and B available online at http://bib.oxfordjournals.org/.

Since the survival comparison was performed across tumor types, potential bias caused by the high level of heterogeneity of tumor types may be the cause for the presented statistical significance. To address this, multivariable analysis was performed using the cox regression model, with TP53_PROF’s predictions and the tumor type as the variables. A *P*-value of 0.005 was obtained for the comparison of D and ND predictions in this analysis as well, indicating that the significant results are maintained independently of the tumor type (Figure 7B). Since the number of ND predicted samples in this analysis is small, a similar comparison in a tumor specific manner is not likely to present with statistically significant results due to lack of power, but it may hint towards a similar pattern. Therefore, survival comparisons were performed on tumors with more than three samples predicted as ND by TP53_PROF, and where general *TP53* mutational state presents with statistically significant survival prediction distinctions: lung adenocarcinoma (LUAD) and uterus corpus endothelial carcinoma (UCEC). There were no death events in the ND predicted group for UCEC (*n* = 4) and only one deceased patient in the same group for LUAD (*n* = 7, Supplementary Figure S14C and D available online at http://bib.oxfordjournals.org/).

## Discussion

Predicting the pathogenicity of somatic or germline genomic variations represents an unmet need in genetic consultation and precision genomic medicine for cancer treatment. Regarding *TP53*, somatic *TP53* status is used in routine clinical practice in several types of cancer such as chronic lymphocytic leukemia (CLL) [64], acute myeloid leukemia (AML) [65] and myelodysplastic syndrome, in order to identify patients likely to benefit from specific treatments. Furthermore, it has been clearly established that germline p53 variants are frequent in familial cancer syndromes, such as LFS or in families with hereditary breast and ovarian cancer, and surveillance of individuals with an identified germline *TP53* mutation is highly beneficial to improve the likelihood of early tumor detection and subsequently improved outcomes. Therefore, there is a necessary need to have accurate information regarding p53 variants. This challenge is complicated by the landscape of p53 variants which is composed predominantly of multiple missense variants spread-out on the entire gene. Among the 2314 possible missense variants in the coding region, 1621 (70%) have been described in at least one tumor and among them only 190 have interpretation in ClinVar, the leading genomic variant database.

In the current study, we described TP53_PROF, a predictive machine learning algorithm aimed to predict the LOF of every possible missense variant in *TP53*. This study was made possible by the conjunction of two factors, namely the large number of p53 variants reported occurrences in the literature (more than 150 000) and the publication of three large-scale functional analysis of more than 15 000 p53 variants. The presence of variants in four independent datasets enables defining a robust positive training set. The fact that almost no new p53 variants are detected (‘saturation’) supports grouping variants that were not reported or reported only once into a negative training set. It was therefore possible to develop a robust original and reliable positive and negative training sets that are the prime requisite for machine learning. The algorithm selected for this model, GBM, uses decision tree structures in an iterative manner to improve residual discrepancies. GBM is considered highly efficient for tabular data and is ideal for the amount of data used to train TP53_PROF. TP53_PROF achieved high level of accuracy of 96.5% on the independent test set. Interestingly, the model using all features had the highest AUC on the test set but the model using the functional features only, provided slightly better accuracy (Figure 2). While accuracy is better oriented towards the model’s final purpose of predicting deleteriousness, it also depends on the final cutoff decision made during validation. This may cause accuracy variations on the test set. AUC on the other hand can be thought of as the expectation of accuracies given all possible cutoffs and can therefore be considered as a more robust estimation of performance.

TP53_PROF was validated using various independent datasets enriched in functional variants (validated *TP53* SNPs in set 41 or gnomAD) or non-functional variants (LFS patients or ClinVar). ClinVar analysis showed that TP53_PROF reach an accuracy of 96.3% and a sensitivity of 95.7%. Benign and likely benign variants in ClinVar were detected with a sensitivity of 100%. LFS database analysis showed accuracy of 93–95.9% and the analysis of 41 gnomAD variants showed accuracy of at least 93%.

We further validated our approach using the survival data of TCGA. Survival of patients with missense variants predicted as non-functional by TP53_PROF is comparable to the survival of patients with truncating variants in *TP53*. By contrast, patients with variants predicted functional by TP53_PROF had longer overall survival that was comparable to patients with no *TP53* mutations. These analyses portray a picture of a highly robust model, with the capability of correctly identifying *TP53* mutations when somatic or germline, benign or pathogenic, in healthy and in sick patients. The discrepancies presented by the model are in almost all cases explainable by technical limitations of the functional assays, or by the complexity of identifying specific variants.

Although, TP53_PROF is very efficient to detect functional variants such as benign SNPs included in gnomAD, we observed a few false negatives linked to variants associated with specific features. First, exonic splice variants, usually localized in codons close to splice junctions, are not readily classified. As functional assays used exogenous cDNA-based expression of p53, variants associated with nonsense-mediated mRNA decay (NMD) such as nonsense, no stop or splice mutations will lead to random results. We have recently shown that some of these variants can be spotted and efficiently classified by data mining and expression data and that *TP53* RNA expression is lower for these variants [33]. Increasing the number of studies that will include both genomic sequencing and expression data will improve the detection of variants that target quantitative and/or qualitative RNA expression.

Second, although variants localized in the DNA-binding domain and linked directly to the transcriptional function of p53 are efficiently classified, we noticed that this is not the case for variants in the oligomerization domains. It has been previously shown that the LOF of variants in this region are more difficult to analyze. This is the case for two well established population specific non-functional p53 variants, i.e*.* p.R337H, associated with familial pediatric adrenocortical carcinoma in Brazil or p.G334R in hereditary breast cancer in Ashkenazi Jews. For both cases as well as for other variants in this domain, classical functional assays using read-out such as growth arrest or transcriptional activities are not efficient. Indeed, examination of functional data from the dominant negative assay of Giacomelli *et al*. [25] shows that all *TP53* variants localized in the oligomerization domain of p53 scored fully active. Refine analysis of these variants using more sensitive and specific assays will be needed to assess the functionality of variants in the oligomerization domain.

Finally, we used the subset of missense variants for which definitive prediction is given in ClinVar and compared to our approach (190 variants). For our full set of labels and predictions (training set and predicting set) the level of accuracy was 96.32%. These numbers represent the accuracy of our approach. ClinVar is enriched by variants predicted to be deleterious (183/190—96.3%). Indeed, all the discrepancies are in variants for which ClinVar predicted non-functionality, our model predicted functionality, and they are all in the prediction set (*n* = 7).

The ACMG guidelines include multiple rules for variant classification. Among them, PS3 (Pathogenic Strong, well-established assay, deleterious effect) and BS3 (Benign Strong, well-established assay, no deleterious effect) rules that are strong but challenging criteria to define the final pathogenicity of a variant. These rules cannot be applied to every gene due to the lack of functional assays suitable to capture a direct readout that can be directly linked to the pathology. Instead, PP3 rules (pathogenic supporting, multiple lines of computational evidence support a deleterious effect on the gene or gene product) or BP4 (benign supporting, multiple lines of computational evidence suggest no impact on gene or gene product) are widely used but they must be used with extreme caution due to their lack of specificity. Indeed, the accuracy of our model enables a more confident application of PP3 and BP4 rules.

A further reaching application of our model can be to create a new Bayesian-based framework that incorporates the model’s prediction with family history to provide a probability score of functionality for individual patients. Such application requires further validation using large databases of clinical genetic consultation that can help tune the framework and provide retrospective validation. The model can also play a role in classification of somatic mutations in *TP53* for therapeutic decision making such as for CLL patients.

Most predictive tools used to assess the effect of amino acid substitutions on the function of a protein rely on non-specific or indirect computational features. In the present analysis, using various *TP53* specific features, we have been able to reach a high accuracy for the prediction of cancer associated p53 variants. Although, as of today, only a few genes can be so deeply interrogated, it is likely that large-scale functional and omics analysis as well as in deep structural studies will be more readily available making the strategy used for the development of TP53_PROF suitable for other genes of clinical interest.

## Data Availability

UMD_TP53 variant database, 2019_R1 version, was used for the analysis and can be downloaded from http://p53.fr/download-the-database/. Functional activity scores were also extracted from UMD_TP53. TP53MUTLOAD is available at http://p53.fr/tp53-database/mutload/. TCGA (mutations and survival) and MSKCC data were downloaded from http://www.cbioportal.org/, updated to October 2019. ICGC data were downloaded from https://dcc.icgc.org/, data release 26, 17 December 2017. gnomAD *TP53* variant data were extracted from version 2.1, available at https://gnomad.broadinstitute.org/downloads.

Computational scores were extracted from dbNSFP version 3.5, available at https://sites.google.com/site/jpopgen/dbNSFP?authuser=0 (Sources of the few exceptions are described in Supplementary Table S8 available online at http://bib.oxfordjournals.org/). LFS cohort data were extracted from (i) the IARC database [40] and (ii) Gao *et al*. [39]. ClinVar’s p53 missense variant annotations can be retrieved from https://www.ncbi.nlm.nih.gov/clinvar. Source code is available at https://github.com/gilbenc/TP53_PROF.

**Key Points**

- Highly accurate gene specific machine learning model predicts the impact of all missense mutations in *TP53.*
- Integrating p53 specific functional assay scores and computational scores as features.
- Unprecedented thorough validation and in-depth examination of model’s performances in clinical and experimental context.
- Model is competitive in identifying deleteriousness (sensitivity) and is far superior over other examined scores in identifying non-deleteriousness (specificity).
- Data assembly and learning methods are generalizable to other major cancer genes.

## Supplementary Material

Supplementary_information_bbab524

supplementary_Figure_S4a_and_b_bbab524

Supplementary_Table_S1_bbab524

Supplementary_Table_S2_bbab524

Supplementary_Table_S5_bbab524

Supplementary_Table_S7_bbab524

Supplementary_Table_S8_bbab524

TP53_variants_update_bbab524

## Notes

**Gil Ben Cohen** is an MD-PhD student at The Hebrew University of Jerusalem, focused on precision oncology and machine learning. He is the main student working on this project.

**Flora Doffe** is a PhD student at the University of Paris-Saclay, who contributed to the functional analysis of p53 variants.

**Mihcal Devir** is a computer science programmer and a research assistant at The Hebrew University of Jerusalem who took part in this project.

**Bernard Leroy** is a computer analyst at Sorbonne University. He developed and maintains the *TP53* mutation database.

**Thierry Soussi** is a professor at Sorbonne University. His research is focused on the analysis of the consequences of *TP53* mutations in human cancer. His analysis provides analytical tools used in clinical routine to decipher and interpret genomic data obtained via tumor sequencing.

**Shai Rosenberg** is a senior lecturer and neuro-oncologist in the (1) Gaffin Center for Neuro-Oncology, Sharett Institute for Oncology, Hadassah Medical Center and Faculty of Medicine, Hebrew University of Jerusalem, Israel and (2) The Wohl Institute for Translational Medicine, Hadassah Medical Center and Faculty of Medicine, Hebrew University of Jerusalem, Israel. His research in focused on cancer genomics, precision medicine and machine learning. He heads the laboratory of Computational Biology of Cancer and the Sagol program for dual degree in medicine and computer science in the Hebrew University.
