# Supplementary material for: TP53_PROF: a machine learning model to predict impact of missense mutations in TP53
Source: Brief Bioinform. 2022 Jan 18;23(2):bbab524. doi: 10.1093/bib/bbab524 (PMC8921628; doi:10.1093/bib/bbab524)
Supplement: Supplementary_information_bbab524 [file supplementary_information_bbab524.pdf]

**Machine learning model to predict impact for missense mutations in *TP53***

By Ben-Cohen et al.

## Supplementary Information

**Supplementary Figures S1 to S13**

**Supplementary figure S1.** *TP53* mutation landscape.

**Supplementary figure S2.** Number of novel p53 missense variants published each year (mutant novelty).

**Supplementary figure S3.** ROC curves for the functional, all features and computational models' predictions performed on the test set.

**Supplementary figure S4.** Functional activity of the 41 p53 variants included in the experimental validation set.

**Supplementary figure S5.** Frequency of multiple mutations (MMF) and Germinal to Somatic (GVS) ratio for p53 variants.

**Supplementary figure S6.** p53 variants anti proliferative activity.

**Supplementary figure S7.** UMD and gnomAD frequency of the 39 non-functional p53 variants included in gnomAD.

**Supplementary figure S8.** Non-functional variants are not enriched by data issued from cancer patients.

**Supplementary figure S9.** p53 variants in gnomad versus UMD (part 1).

**Supplementary figure S10.** p53 variants in gnomad versus UMD (Part 2A and 2B).

**Supplementary Figure S11:** TP53_PROF analysis of two LFS cohorts.

**Supplementary Figure S12.** ClinVar data used for the analysis.

**Supplementary Figure S13.** ClinVar analysis using PROF.

**Supplementary Figure S14.** Survival curve of tumors from TCGA database

**Supplementary Tables S1 to S5**

**Supplementary table S1.** p53 variants used for the positive and negative training. **(Excel file)**

For each protein variant, all possible single nucleotide substitutions are shown.

**Supplementary table S2.** Curated dataset of 1,294 variants, with their labels and their values for the 42 features used for TP53_PROF. **(Excel file)**

**Supplementary table S3.** Hyperparameters used for three final GBM models.

**Supplementary table S4.** Three models result on the validation set.

**Supplementary table S5.** 2,314 p53 missense variants with their TP53_PROF functional model predictions. **(Excel file)**

**Supplementary table S6.** Variable Importance scores of the functional features according to the functional model.

**Supplementary table S7.** In silico and functional analysis of p53 variants from set 41. **(Excel file)**

**Supplementary table S8.** Features used for training TP53_PROF. **(Excel file)**

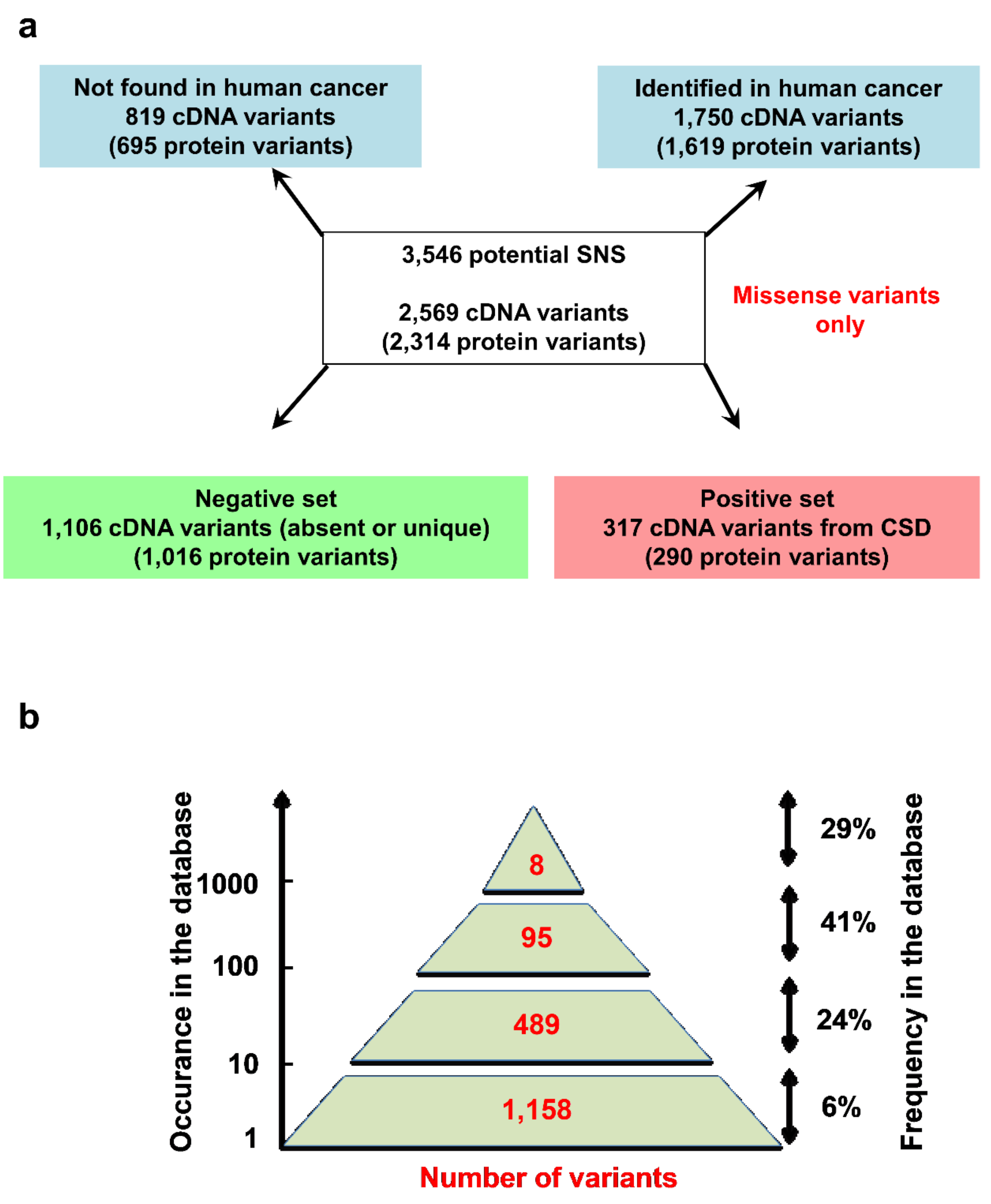
a
Not found in human cancer
819 cDNA variants
(695 protein variants)
Identified in human cancer
1,750 cDNA variants
(1,619 protein variants)
3,546 potential SNS
2,569 cDNA variants
(2,314 protein variants)
Missense variants only
Negative set
1,106 cDNA variants (absent or unique)
(1,016 protein variants)
Positive set
317 cDNA variants from CSD
(290 protein variants)
b
Occurance in the database
1000
100
10
1
8
95
489
1,158
29%
41%
24%
6%
Frequency in the database
Number of variants

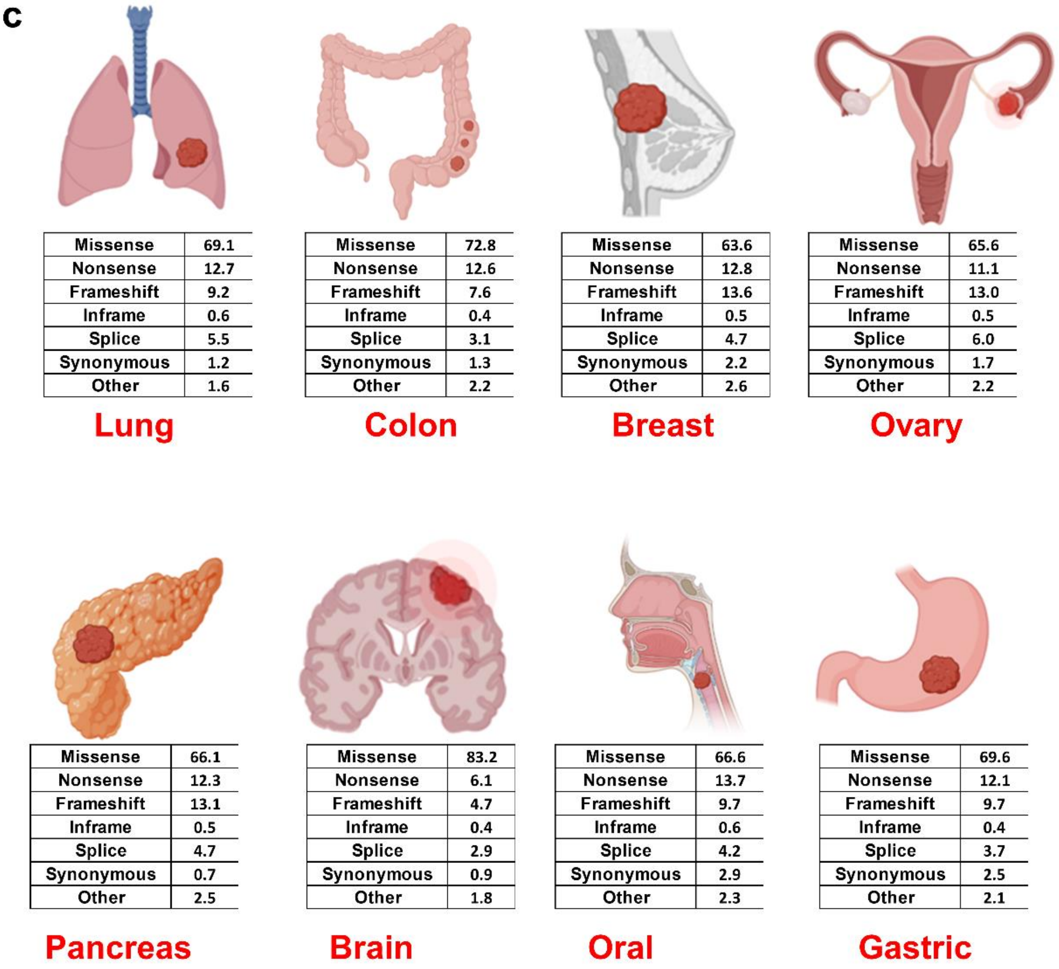


| | |
|---|---|
| Missense | 69.1 |
| Nonsense | 12.7 |
| Frameshift | 9.2 |
| Inframe | 0.6 |
| Splice | 5.5 |
| Synonymous | 1.2 |
| Other | 1.6 |

| | |
|---|---|
| Missense | 72.8 |
| Nonsense | 12.6 |
| Frameshift | 7.6 |
| Inframe | 0.4 |
| Splice | 3.1 |
| Synonymous | 1.3 |
| Other | 2.2 |

| | |
|---|---|
| Missense | 63.6 |
| Nonsense | 12.8 |
| Frameshift | 13.6 |
| Inframe | 0.5 |
| Splice | 4.7 |
| Synonymous | 2.2 |
| Other | 2.6 |

| | |
|---|---|
| Missense | 65.6 |
| Nonsense | 11.1 |
| Frameshift | 13.0 |
| Inframe | 0.5 |
| Splice | 6.0 |
| Synonymous | 1.7 |
| Other | 2.2 |

| | |
|---|---|
| Missense | 66.1 |
| Nonsense | 12.3 |
| Frameshift | 13.1 |
| Inframe | 0.5 |
| Splice | 4.7 |
| Synonymous | 0.7 |
| Other | 2.5 |

| | |
|---|---|
| Missense | 83.2 |
| Nonsense | 6.1 |
| Frameshift | 4.7 |
| Inframe | 0.4 |
| Splice | 2.9 |
| Synonymous | 0.9 |
| Other | 1.8 |

| | |
|---|---|
| Missense | 66.6 |
| Nonsense | 13.7 |
| Frameshift | 9.7 |
| Inframe | 0.6 |
| Splice | 4.2 |
| Synonymous | 2.9 |
| Other | 2.3 |

| | |
|---|---|
| Missense | 69.6 |
| Nonsense | 12.1 |
| Frameshift | 9.7 |
| Inframe | 0.4 |
| Splice | 3.7 |
| Synonymous | 2.5 |
| Other | 2.1 |

**Supplementary figure S1.** TP53 mutation landscape.

**A** Mutability of *TP53*. Two thousand three hundred and fourteen different missense protein variants can be issued from the open reading frame of *TP53*. About 30% of these variants have never been identified in any database (UMD, TCGA, MSKSCC or GENIE; 2019 release). Variants selected for the negative and positive sets are shown in the lower part of the figure.

**b** Frequency of p53 variants in the UMD TP53 database (2019 release). p53 variants have been split in four classes according to their occurrence in the database (1-10, 11-100; 101-1,000 and more than 1 ,000). Left axis: occurrence of each variant in patients included in UMD (log scale). Nine variants (p.R175H, p.R248W, p.R273C, p.R273H, p.R282W, p.R248Q, p.Y220C, p.G245S and p.R249S) have been reported in more than 1,000 patients; right axis: cumulated frequency of p53 variants in the four classes. Only missense variants have been analyzed in this graph. SNS: single nucleotide substitution.

**c** Spectrum of p53 variants in different tumor types. Although, the frequency of *TP53* mutations can vary across the types of cancer, the spectrum remains comparable with a high predominance of missense variants.

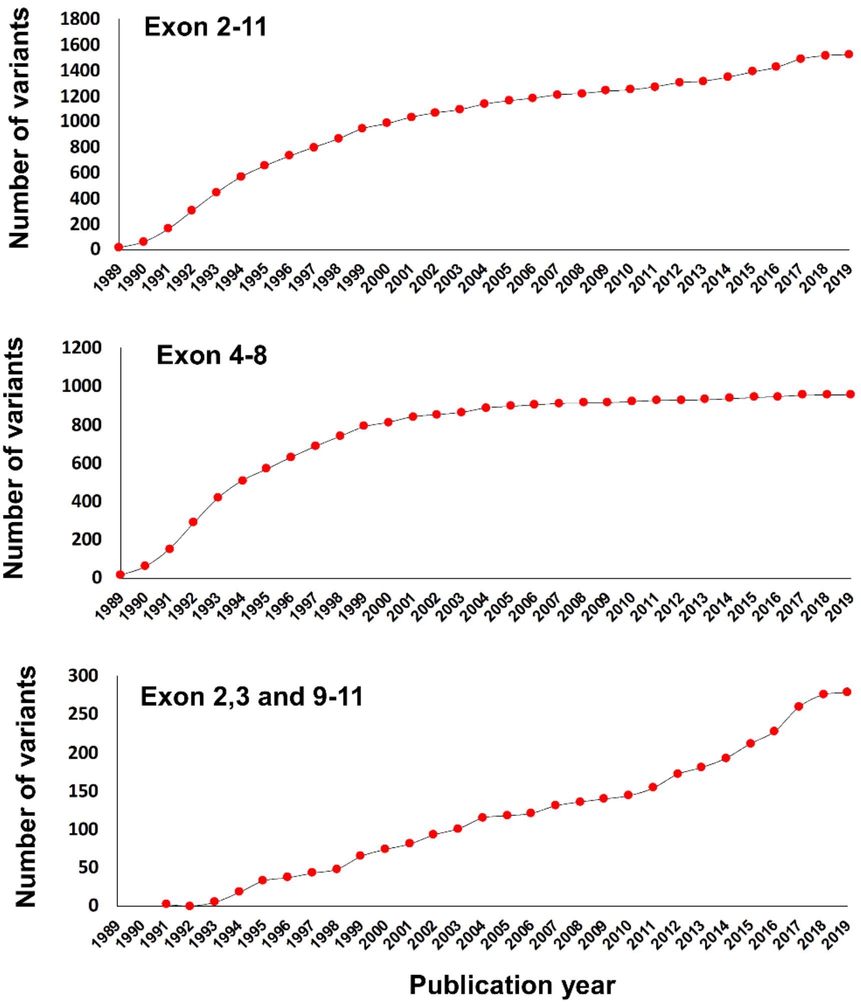


**Supplementary figure S2.** Number of novel p53 missense variants published each year (mutant novelty).

During the first 10 years (1989-1999), the number of novel variants detected in human tumors has increased steadily with the frequent description of new variants. This increase has then slowed down and has reached a plateau indicating that a saturation point has been reached with the description of all potential missense variants associated with a tumor suppressive defect. Data for the DNA binding domain (exon 4-8) have quickly reached a plateau due to the high number of studies focusing on this region. Data corresponding to the amino and carboxy-terminus (exon 2,3 and 9-11) have plateaued at a slower pace. Data used for this analysis is issued from the 2019 release of the UMD_TP53 database (http://p53.fr).

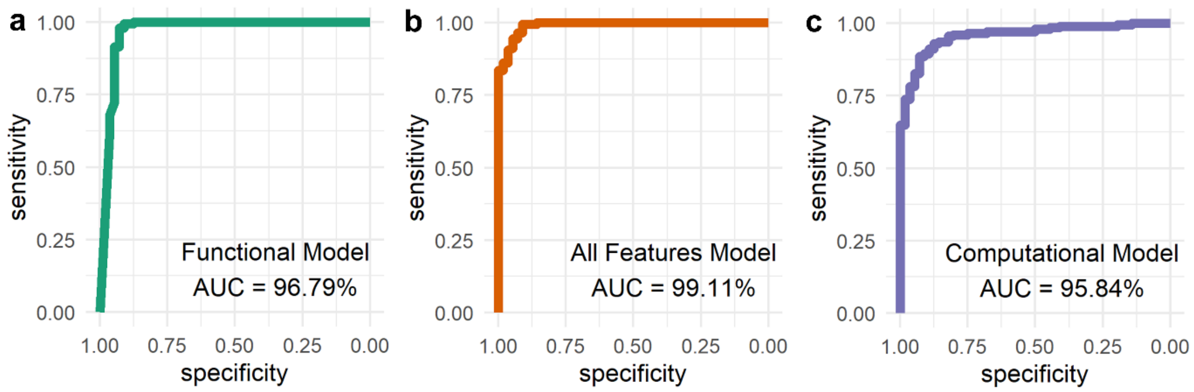


**Supplementary Figure S3.** ROC curves for TP53_PROF performance on the **a** functional, **b** all-features and **c** computational models on the test set.

For Figure S4, See separate PDF file.

**Supplementary figure S4.** Functional activity of the 41 p53 variants included in the experimental validation set.

**a** The 12 different activity readouts have been defined as deleterious (red), partially deleterious (yellow) or not deleterious (green) according to the remaining p53 activity(1).

**b** Transcriptional activity for eight *TP53* response elements analyzed in yeast are from Kato et al.(2). For studies performed in mammalian cells, data were issued from the work of Kotler et al.(3). Growth activity of all variants localized in the DNA binding domain of p53 was assessed in H1299 cells. Only variants between residues 100 and 300 have been reported in this study. The third set corresponds to the study of Giacomelli et al.(4). In this analysis, dominant negative activity (p53WT_Nut), loss of function (p53NULL_nut) and response to etoposide (p53NULL_Eto) were analyzed in mammalian cells for 8,258 p53 variants. Taken together, 14 different readouts for p53 function were available for most variants.

Green: functional activity is not impaired; yellow: functional activity is partially impaired; Red: functional activity is totally impaired. Activity of each variant is indicated by the diamond.

Data from Kato et al., was normalized from 0 (no activity) to 1 (full activity) as described previously(5). Data from Kotler et al or Giacomelli et al corresponds to the score published by the authors.

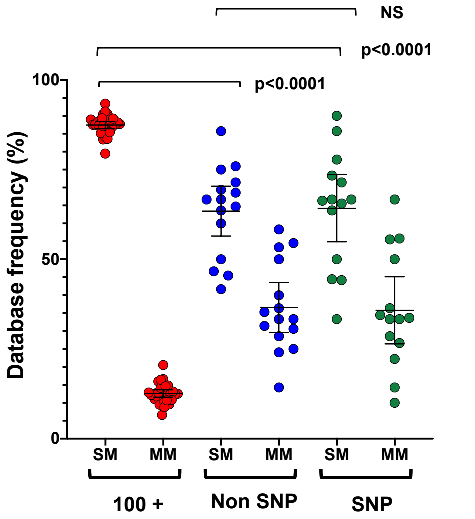


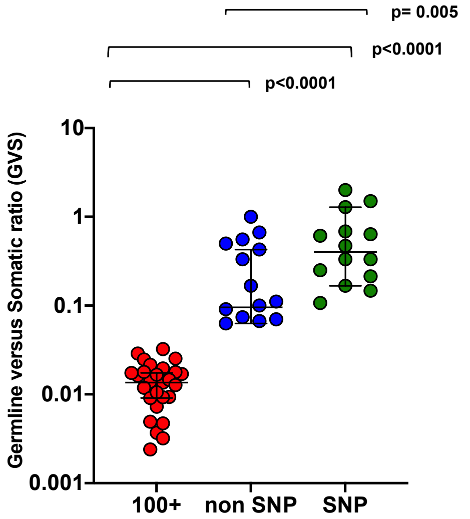


**Supplementary figure S5.** Frequency of multiple mutations (MMF) and Germinal to Somatic (GVS) ratio for p53 variants.

**a** The frequency at which each p53 variant occurs as a single variant (SM) or is associated with a second variant (MM) is indicated on the Y-axis. 100+: Hot spot p53 variants found more than 100 times in UMD. Non-SNP: p53 variants from the 41 datasets that are not considered to be polymorphic variants. SNP: Newly discovered p53 SNP included in the UMD database.

The two common p53 SNP (p.P72R and p.47S) are not included in the UMD and are not included in the present analysis.

**b** GVS ratio is depicted on the Y-axis (log scale). The three sets of p53 variant are described in A.

Statistical significance was indicated by t tests.

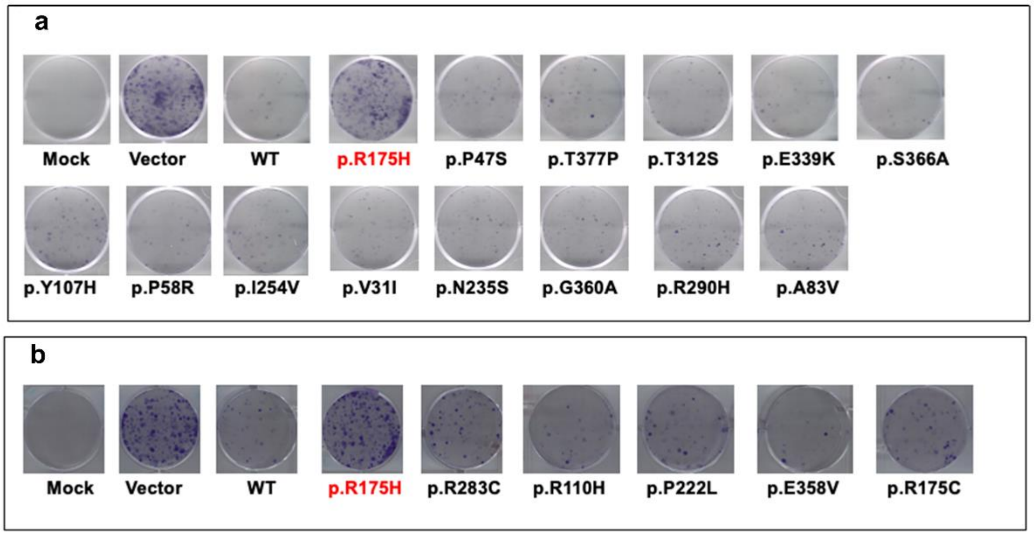


**Supplementary figure S6.** p53variants anti proliferative activity. Representative photos of colony formation assay. Plates were stained 2 weeks after transfection. Wild type p53 as well as p53 variants are able to inhibit colony formation whereas cancer associated variant, p.R175H, used as a positive control, is defective. **a** and **b** are two different experiments.

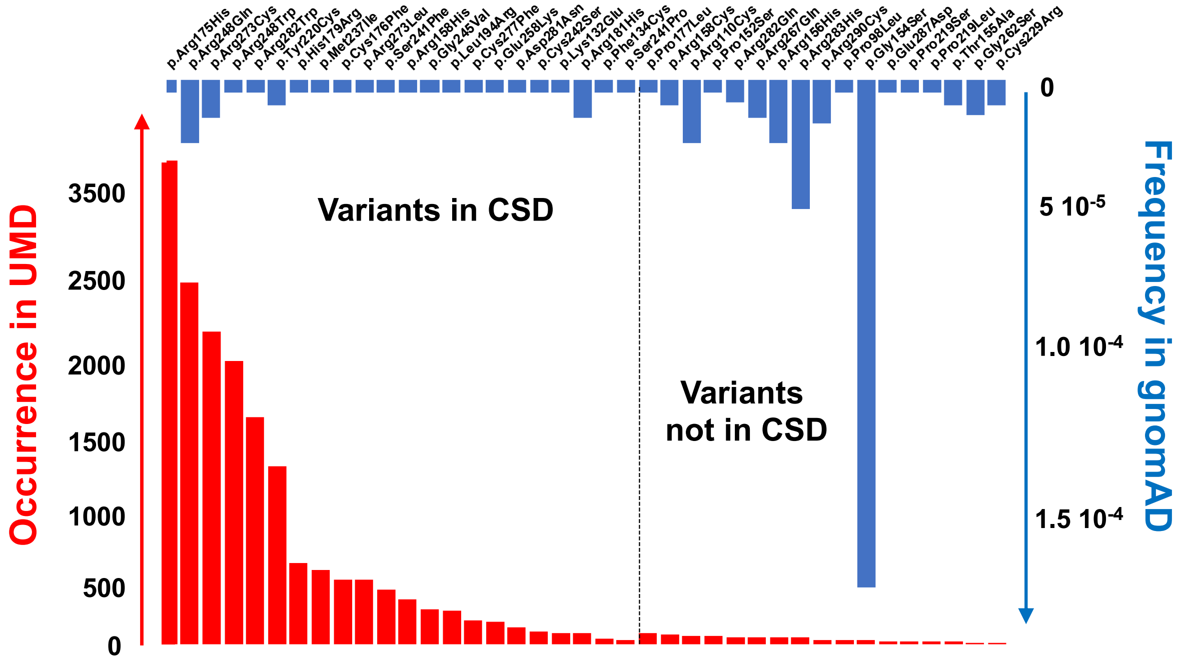


**Supplementary figure S7.** UMD and gnomAD frequency of the 39 non-functional p53 variants included in gnomAD.

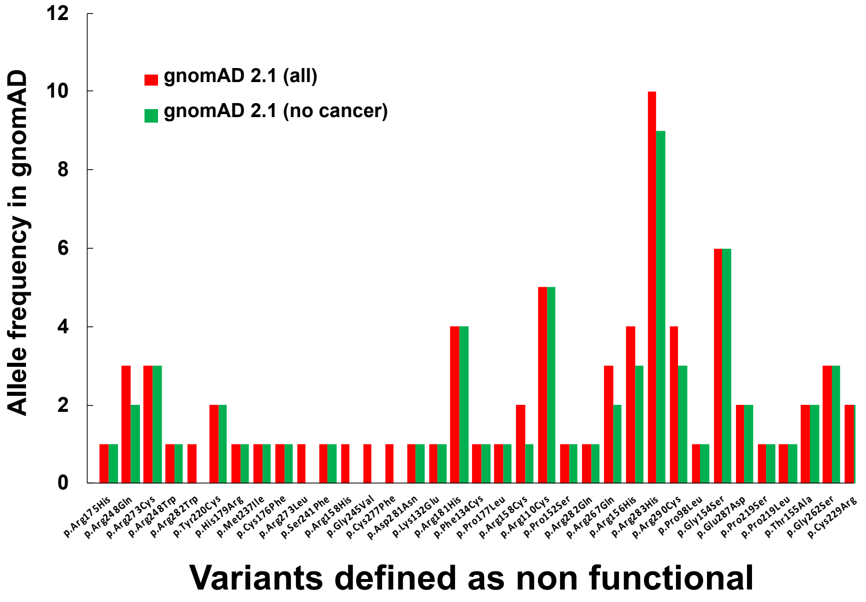


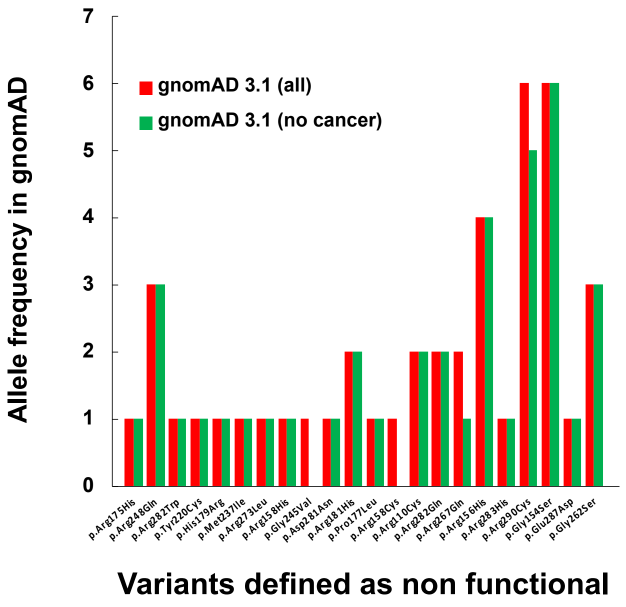


**Supplementary figure S8.** Non-functional p53 variants are not enriched by data issued from cancer patients.

## Variants defined as functional absent from UMD

| | Frequency in gnomAD | Frequency in UMD | Comment |
|---|---|---|---|
| p.Pro72Arg | | Absent | SNP |
| p.Pro47Ser | | Absent | SNP |
| p.Val31Ile | | Absent | SNP |
| p.Asn235Ser | | Absent | SNP |
| p.Gly360Ala | | Absent | SNP |
| p.Arg290His | | Absent | SNP |
| p.Tyr107His | | Absent | SNP |
| p.Pro58Arg | | Absent | SNP |
| p.Arg283Cys | | Absent | SNP |
| p.Thr312Ser | | Absent | SNP |
| p.Glu339Lys | | Absent | SNP |
| p.Glu11Gln | | Absent | SNP |
| p.Arg110His | | Absent | SNP |
| p.Gly389Arg | | Absent | No status |
| p.Ala189Val | | Absent | SNP |
| p.Pro47Thr | | Absent | No status |
| p.Glu358Val | | Absent | SNP |
| p.Ala76Glu | | Absent | No status |
| p.Gln5Arg | | Absent | No status |
| p.Ser315Thr | | Absent | No status |
| p.Asp49His | | Absent | SNP |
| p.Asp49Gly | | Absent | No status |
| p.Ser116Ala | | Absent | No status |
| p.Arg333Gly | | Absent | No status |
| p.Glu339Val | | Absent | No status |
| p.Leu350Val | | Absent | No status |
| p.Lys357Arg | | Absent | No status |
| p.Gly360Glu | | Absent | No status |
| p.Ser362Cys | | Absent | No status |
| p.Arg363Gly | | Absent | No status |
| p.His368Gln | | Absent | No status |
| p.Thr377Ser | | Absent | No status |
| p.Met384Thr | | Absent | No status |
| p.Thr387Arg | | Absent | No status |
| p.Asp393Tyr | | Absent | No status |
| p.Ser6Pro | | Absent | No status |
| p.Asp57Gly | | Absent | No status |
| p.Ala78Gly | | Absent | No status |
| p.Leu114Ser | | Absent | No status |
| p.Gly117Ala | | Absent | No status |
| p.Pro12Arg | | Absent | No status |
| p.Glu2Lys | | Absent | No status |
| p.Asp21Glu | | Absent | No status |
| p.Glu326Asp | | Absent | No status |

**Supplementary figure S9.** p53 variants defined as functional and absent from UMD. The most frequent variants in gnomAD are functional SNPs.

# gnomAD variants defined as functional included in UMD (part a)

| | Frequency in gnomAD | Frequency in UMD | | Comment |
|---|---|---|---|---|
| p.Glu224Asp | | | 74 | Pathogenic splice variant |
| p.Arg175Cys | | | 71 | Passenger mutation |
| p.Thr125Met | | | 59 | Pathogenic splice variant |
| p.Arg337His | | | 47 | Pathogenic variant |
| p.Arg156Cys | | | 36 | NA |
| p.Met160Ile | | | 33 | NA |
| p.Glu287Lys | | | 31 | NA |
| p.Val157Ile | | | 30 | NA |
| p.Pro222Leu | | | 28 | NA |
| p.Thr170Met | | | 25 | NA |
| p.Arg196Gln | | | 24 | NA |
| p.Arg202Cys | | | 23 | NA |
| p.Gly108Ser | | | 22 | NA |
| p.Asp49His | | | 20 | NA |
| p.Glu298Lys | | | 19 | NA |
| p.Pro142Leu | | | 18 | NA |
| p.Ile254Val | | | 17 | NA |
| p.Pro82Leu | | | 16 | NA |
| p.Gly154Asp | | | 16 | NA |
| p.Gly293Arg | | | 16 | NA |
| p.Glu298Gln | | | 16 | NA |
| p.Pro295Leu | | | 15 | NA |
| p.Pro191Leu | | | 15 | NA |
| p.Val217Met | | | 15 | NA |
| p.Pro72Ala | | | 14 | NA |
| p.Asp186Asn | | | 14 | NA |
| p.Met160Val | | | 14 | NA |
| p.Thr155Ser | | | 12 | NA |
| p.Thr304Ala | | | 12 | NA |
| p.Gly293Trp | | | 12 | NA |
| p.Glu221Lys | | | 11 | NA |
| p.Lys292Arg | | | 10 | NA |
| p.Gln317Lys | | | 10 | NA |
| p.Met133Ile | | | 10 | NA |
| p.Glu11Lys | | | 10 | NA |
| p.Asp259Gly | | | 10 | NA |
| p.Pro295Ser | | | 9 | NA |
| p.Val73Met | | | 9 | NA |
| p.Lys291Arg | | | 9 | NA |
| p.Asn263Asp | | | 8 | NA |
| p.His214Gln | | | 8 | NA |
| p.Asn131Lys | | | 8 | NA |
| p.Pro36Leu | | | 8 | NA |
| p.Gln317Arg | | | 8 | NA |
| p.Ala83Val | | | 7 | NA |
| p.Ser366Ala | | | 7 | NA |
| p.Val10Ile | | | 7 | NA |
| p.Arg342Gln | | | 7 | NA |
| p.Asp49Asn | | | 7 | NA |
| p.Ser185Asn | | | 7 | NA |
| p.Thr329Ile | | | 7 | NA |
| p.Met133Val | | | 7 | NA |
| p.Arg333Cys | | | 6 | NA |
| p.Pro72Ser | | | 6 | NA |
| p.Asn235Ile | | | 6 | NA |

**Supplementary figure S10a.**

# gnomAD variants defined as functional included in UMD (part b)

| | Frequency in gnomAD | Frequency in UMD | | Comment |
|---|---|---|---|---|
| p.Pro72Ser | | | 6 | NA |
| p.Asn235Ile | | | 6 | NA |
| p.His365Tyr | | | 6 | NA |
| p.Arg333His | | | 5 | NA |
| p.Glu204Gly | | | 5 | NA |
| p.Gly112Ser | | | 5 | NA |
| p.Asp148Tyr | | | 5 | NA |
| p.Glu294Asp | | | 5 | NA |
| p.Arg306Gln | | | 5 | NA |
| p.Gly360Val | | | 4 | NA |
| p.Arg379His | | | 4 | NA |
| p.Leu35Phe | | | 4 | NA |
| p.Gln167Pro | | | 4 | NA |
| p.Pro191Arg | | | 4 | NA |
| p.Gln354Arg | | | 4 | NA |
| p.Gly117Glu | | | 4 | NA |
| p.Pro191His | | | 4 | NA |
| p.Gly356Arg | | | 4 | NA |
| p.Gly334Glu | | | 3 | NA |
| p.Asp324His | | | 3 | NA |
| p.Ala74Val | | | 3 | NA |
| p.Cys124Ser | | | 3 | NA |
| p.Glu339Gln | | | 3 | NA |
| p.Gly360Arg | | | 3 | NA |
| p.Asp61Gly | | | 3 | NA |
| p.Pro72Thr | | | 3 | NA |
| p.Pro13Ser | | | 3 | NA |
| p.Ser314Phe | | | 3 | NA |
| p.Asp324Asn | | | 3 | NA |
| p.Asp324Gly | | | 3 | NA |
| p.Gly325Val | | | 3 | NA |
| p.Pro77Ser | | | 2 | NA |
| p.Gly59Cys | | | 2 | NA |
| p.Gln354Lys | | | 2 | NA |
| p.Ala63Val | | | 2 | NA |
| p.Ala84Thr | | | 2 | NA |
| p.Asn310Lys | | | 2 | NA |
| p.Arg335His | | | 2 | NA |
| p.Phe338Leu | | | 2 | NA |
| p.Ser367Asn | | | 2 | NA |
| p.Gly374Cys | | | 2 | NA |
| p.Arg379Cys | | | 2 | NA |
| p.Asp42Asn | | | 2 | NA |
| p.Arg283Ser | | | 2 | NA |
| p.Leu323Val | | | 1 | NA |
| p.Gly108Cys | | | 1 | NA |
| p.Gly374Arg | | | 1 | NA |
| p.Ser362Asn | | | 1 | NA |
| p.Arg379Ser | | | 1 | NA |
| p.Leu43Phe | | | 1 | NA |
| p.Gln52Arg | | | 1 | NA |
| p.Asp7His | | | 1 | NA |
| p.Pro77Thr | | | 1 | NA |
| p.Ala83Glu | | | 1 | NA |
| p.Ser9Arg | | | 1 | NA |
| p.Val10Gly | | | 1 | NA |
| p.Gly112Ala | | | 1 | NA |
| p.Leu114Phe | | | 1 | NA |
| p.Asp186Glu | | | 1 | NA |
| p.Val225Leu | | | 1 | NA |
| p.Pro316Thr | | | 1 | NA |

**Supplementary figure S10b.**

**a**

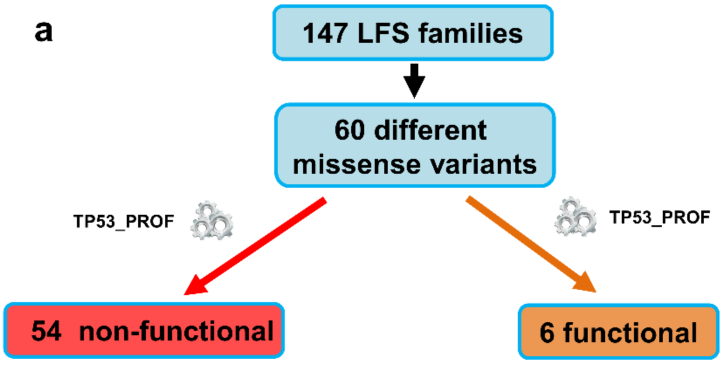


| Variant | Frequency in UMD | Frequency in gnomAD | Status |
|---|---|---|---|
| p.R290H | 0 | 0.00015 | SNP |
| p.Q144L | 25 | absent | Unknown |
| p.K292I | 1 | absent | Unknown |
| p.R290L | 14 | absent | Unknown |
| p.N235S | 0 | 0.00019 | SNP |
| p.R196Q | 59 | 3.976E-06 | Unknown |

**b**

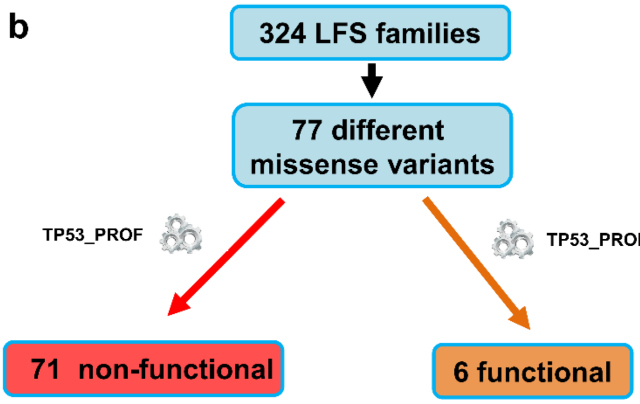


| Variant | Frequency in UMD | Frequency in gnomAD | Status |
|---|---|---|---|
| p.A347D | 4 | absent | Unknown |
| p.G334R | 4 | 0.000003990 | Unknown |
| p.I254V | 0 | 0.00001590 | SNP |
| p.R181C | 101 | absent | Pathogenic |
| p.R283C | 0 | 0.0000742 | SNP |
| p.R290H | 0 | 0.00015 | SNP |

**Supplementary Figure S11.** TP53_PROF analysis of two LFS cohorts. **a** *TP53* cohort from the IARC database; **b** *TP53* cohort from Gao et al. Tables in A and B detail the false negative p53 variants.

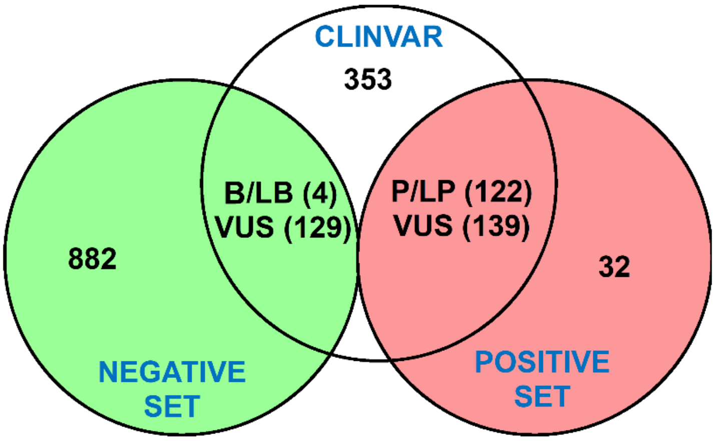


**Supplementary Figure S12.** Clinvar data used for the analysis.

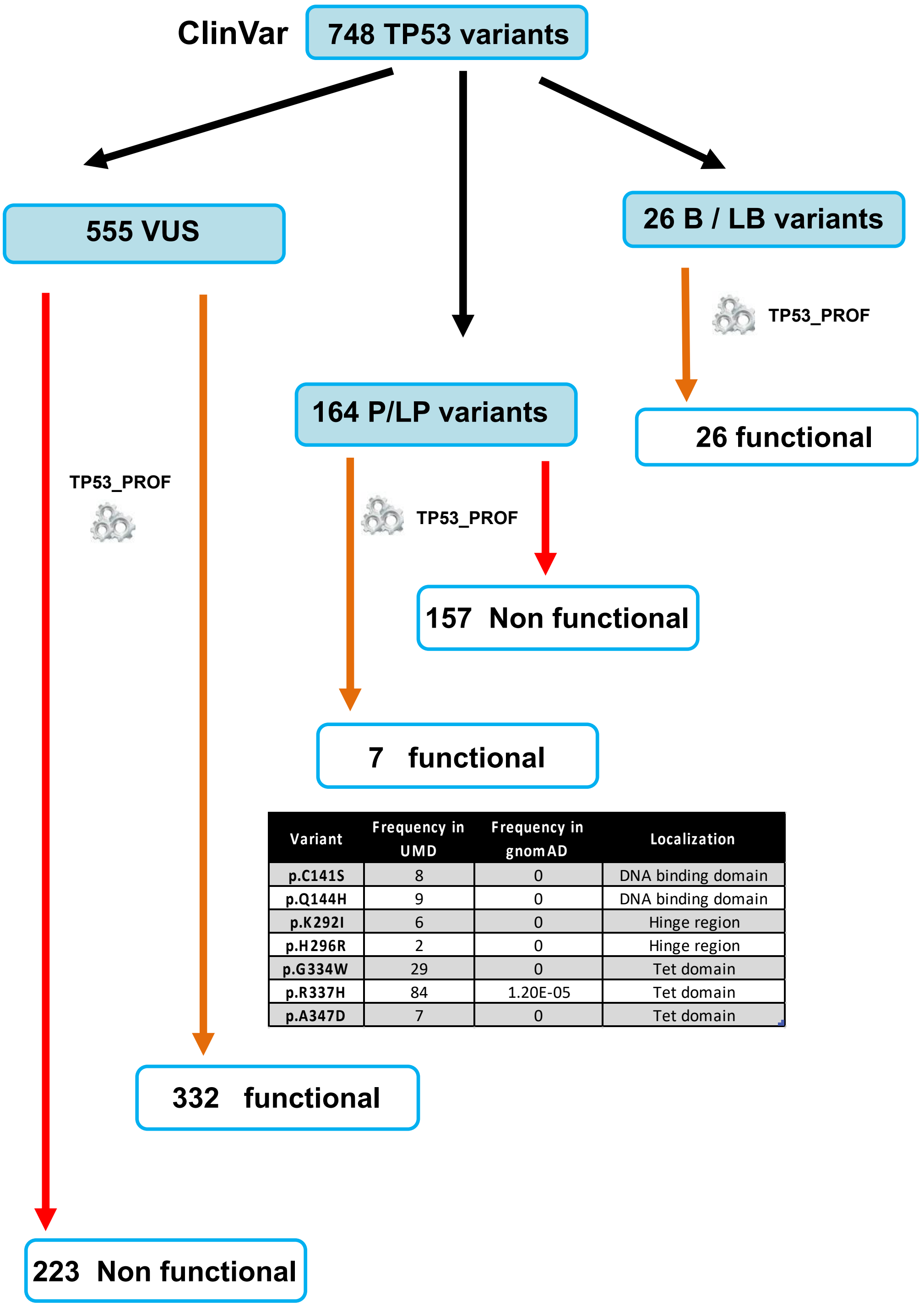


| Variant | Frequency in UMD | Frequency in gnomAD | Localization |
|---|---|---|---|
| p.C141S | 8 | 0 | DNA binding domain |
| p.Q144H | 9 | 0 | DNA binding domain |
| p.K292I | 6 | 0 | Hinge region |
| p.H296R | 2 | 0 | Hinge region |
| p.G334W | 29 | 0 | Tet domain |
| p.R337H | 84 | 1.20E-05 | Tet domain |
| p.A347D | 7 | 0 | Tet domain |

**Supplementary Figure S13:** Clinvar analysis using PROF.

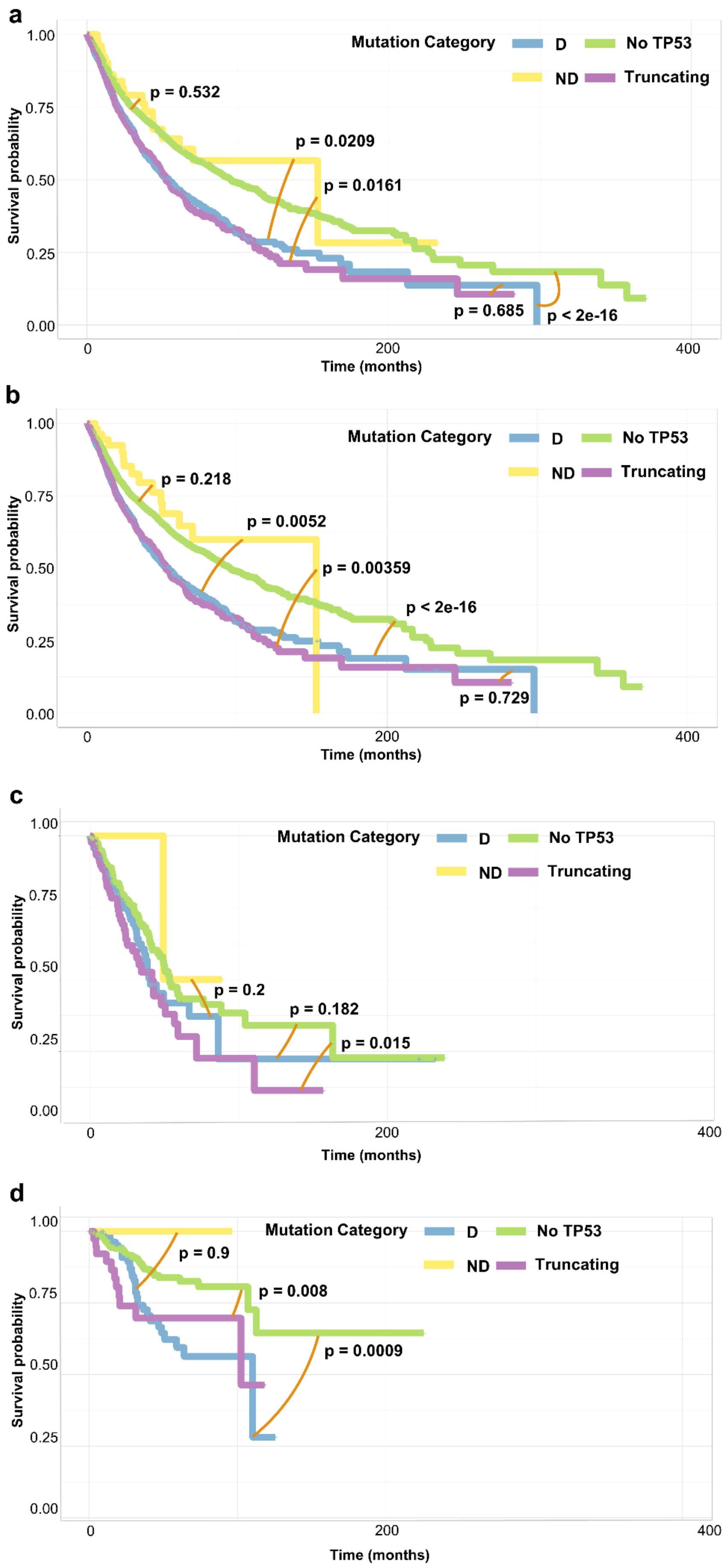

a
Survival probability
1.00
0.75
0.50
0.25
0.00
Mutation Category
D
No TP53
ND
Truncating
p = 0.532
p = 0.0209
p = 0.0161
p = 0.685
p < 2e-16
0
200
400
Time (months)
b
Survival probability
1.00
0.75
0.50
0.25
0.00
Mutation Category
D
No TP53
ND
Truncating
p = 0.218
p = 0.0052
p = 0.00359
p < 2e-16
p = 0.729
0
200
400
Time (months)
c
Survival probability
1.00
0.75
0.50
0.25
0.00
Mutation Category
D
No TP53
ND
Truncating
p = 0.2
p = 0.182
p = 0.015
0
200
400
Time (months)
d
Survival probability
1.00
0.75
0.50
0.25
0.00
Mutation Category
D
No TP53
ND
Truncating
p = 0.9
p = 0.008
p = 0.0009
0
200
400
Time (months)

**Supplementary Figure S14.** Survival curve of tumors from TCGA database. **a** The model based on computational features. **b** The model based on all features. Survival curves for TCGA tumor samples are presented in four groups, separated by their *TP53* mutational state. Green: Samples with no *TP53* mutation (No *TP53*). Yellow: Missense *TP53* mutations predicted by TP53_PROF to be non-deleterious (ND). Blue: Missense *TP53* mutations predicted by TP53_PROF to be deleterious (D). Purple: Samples with a truncating p53 mutation (Truncating). P-values for the comparison between these groups are also shown, with an orange line indicating the two curves being compared. (A) Survival predictions are distinct when comparing No *TP53* and D (p < 2e-16), when comparing ND and D (p = 0.0209), and when comparing ND and Truncating (p = 0.0161), Survival predictions are indistinctive for comparisons between D and Truncating (p = 0.685) and between ND and No *TP53* (0.532). (B) Survival predictions are distinct when comparing No *TP53* and D (p < 2e-16), when comparing ND and D (p = 0.0052), and when comparing ND and Truncating (p = 0.00359), Survival predictions are indistinctive for comparisons between D and Truncating (p = 0.729) and between ND and No *TP53* (0.218). **c + d** Survival analysis performed on TCGA samples of specific tumors, Lung Adenocarcinoma (LUAD, 13c) and Uterus corpus endothelial carcinoma (UCEC, 13d). The tumor types were selected due to distinct survival predictions for samples with or without a mutation (all types of mutations) in *TP53* and based on the relatively larger number of samples predicted as ND (n = 7 in LUAD, n = 4 in UCEC). For LUAD, survival predictions are distinct when comparing truncating and No *TP53* samples (p = 0.015), but not when comparing D to No *TP53* (p = 0.182). No statistically significant survival prediction was observed for D compared to ND samples (p = 0.2). For UCEC, survival predictions are distinct when comparing truncating and No *TP53* samples (p = 0.008) and when comparing D and No *TP53* samples (p = 0.0009), and not for D and ND samples (p = 0.9). This is likely due to the lack of power in both tests Indeed, the few ND samples tend to be like samples not mutated in *TP53*, with only 1 event for patients in the LUAD cohort and no events in the UCEC cohort.

| | All features | Functional | Computational |
|---|---|---|---|
| **Nrounds** | 26 | 30 | 30 |
| **Eta** | 0.09495805 | 0.08622785 | 0.09014091 |
| **Max_depth** | 5 | 3 | 3 |
| **gamma** | 0.2551619 | 0.2804512 | 0.2257494 |
| **colsample_bytree** | 0.7369411 | 0.7805748 | 0.4595327 |
| **min_child_weight** | 1 | 1 | 1 |
| **subsample** | 0.8628348 | 0.729023 | 0.8270071 |
| **cutoff** | 0.4057707 | 0.2724045 | 0.1421284 |

**Supplementary table S3.** Hyperparameters used for the three final GBM models as selected in the validation process.

| GBM | All features model | | Computational model | | Functional model | |
|---|---|---|---|---|---|---|
| | **Label** | | | | | |
| **Prediction** | **D** | **ND** | **D** | **ND** | **D** | **ND** |
| **D** | 55 | 6 | 54 | 27 | 53 | 2 |
| **ND** | 1 | 196 | 2 | 175 | 3 | 200 |
| **AUC** | 99.66% | | 97.64% | | 99.66% | |
| **Accuracy** | 97.29% | | 88.76% | | 98.06% | |

**Supplementary table S4a.** GBM models performance on the validation set.

| RF | All features model | | Computational model | | Functional model | |
|---|---|---|---|---|---|---|
| | Label | | | | | |
| **Prediction** | **D** | **ND** | **D** | **ND** | **D** | **ND** |
| **D** | 55 | 9 | 52 | 16 | 55 | 12 |
| **ND** | 1 | 193 | 4 | 186 | 1 | 190 |
| **AUC** | 99.56% | | 97.62% | | 99.76% | |
| **Accuracy** | 96.12% | | 92.25% | | 94.96% | |

**Supplementary table S4b.** Random Forests models performance on the validation set.

| | 10 tuned runs AUC | | | | | Mean |
|---|---|---|---|---|---|---|
| **GBM All-features** | 99.18% | 98.97% | 99.17% | 99.27% | 99.31% | 99.17% |
| | 99.15% | 98.92% | 99.36% | 99.20% | 99.16% | |
| **GBM Functional** | 99.74% | 99.72% | 99.76% | 99.72% | 99.71% | 99.74% |
| | 99.69% | 99.73% | 99.83% | 99.73% | 99.76% | |
| **GBM Computational** | 97.20% | 97.38% | 97.18% | 97.43% | 97.22% | 97.29% |
| | 97.22% | 97.49% | 97.36% | 97.24% | 97.22% | |
| **RF All-features** | 98.39% | 97.68% | 98.49% | 97.98% | 98.27% | 97.97% |
| | 97.83% | 97.26% | 98.33% | 97.82% | 97.69% | |
| **RF Functional** | 99.73% | 99.73% | 99.68% | 99.68% | 99.73% | 99.72% |
| | 99.64% | 99.75% | 99.77% | 99.80% | 99.77% | |
| **RF computational** | 97.33% | 97.71% | 97.52% | 97.64% | 97.52% | 97.57% |
| | 97.45% | 97.65% | 97.65% | 97.60% | 97.60% | |

**Supplementary table S4c.** GBM and RF 10 runs comparison of AUC on the validation set. Mean of the 10 runs is given.

| | **TP53_PROF Variable Importance** |
|---|---|
| **Giac_A549_WT_Nut_norm** | 0.436425 |
| **Kotler_RFS_H1299_norm** | 0.357332 |
| **percent_14_3_3_s** | 0.053113 |
| **AIP_percent** | 0.042953 |
| **WAF1_percent** | 0.030313 |
| **Giac_A549_Null_Eto_norm** | 0.028602 |
| **Median_activity** | 0.020142 |
| **BAX_percent** | 0.010444 |
| **p53R2_percent** | 0.007311 |
| **GADD45_percent** | 0.007138 |
| **Giac_A549_Null_Nut_norm** | 0.003498 |
| **NOXA_percent** | 0.00147 |
| **Average_activity** | 0.00073 |
| **MDM2_percent** | 0.000528 |

**Supplementary Table S6.** Variable importance of functional features in TP53_PROF

## References


1. Carbonnier V, Leroy B, Rosenberg S, Soussi T. Comprehensive assessment of TP53 loss of function using multiple combinatorial mutagenesis libraries. Scientific reports. 2020;10(1):20368.
2. Kato S, Han SY, Liu W, Otsuka K, Shibata H, Kanamaru R, et al. Understanding the function-structure and function-mutation relationships of p53 tumor suppressor protein by high-resolution missense mutation analysis. Proc Natl Acad Sci U S A. 2003;100(14):8424-9.
3. Kotler E, Shani O, Goldfeld G, Lotan-Pompan M, Tarcic O, Gershoni A, et al. A Systematic p53 Mutation Library Links Differential Functional Impact to Cancer Mutation Pattern and Evolutionary Conservation. Molecular cell. 2018;71(5):873.
4. Giacomelli AO, Yang X, Lintner RE, McFarland JM, Duby M, Kim J, et al. Mutational processes shape the landscape of TP53 mutations in human cancer. Nature genetics. 2018;50(10):1381-7.
5. Soussi T, Kato S, Levy PP, Ishioka C. Reassessment of the TP53 mutation database in human disease by data mining with a library of TP53 missense mutations. Hum Mutat. 2005;25(1):6-17.
