# Supplementary material for: TP53_PROF: a machine learning model to predict impact of missense mutations in TP53
Source: Brief Bioinform. 2022 Jan 18;23(2):bbab524. doi: 10.1093/bib/bbab524 (PMC8921628; doi:10.1093/bib/bbab524)
Supplement: supplementary_Figure_S4a_and_b_bbab524 [file supplementary_figure_s4a_and_b_bbab524.pdf]

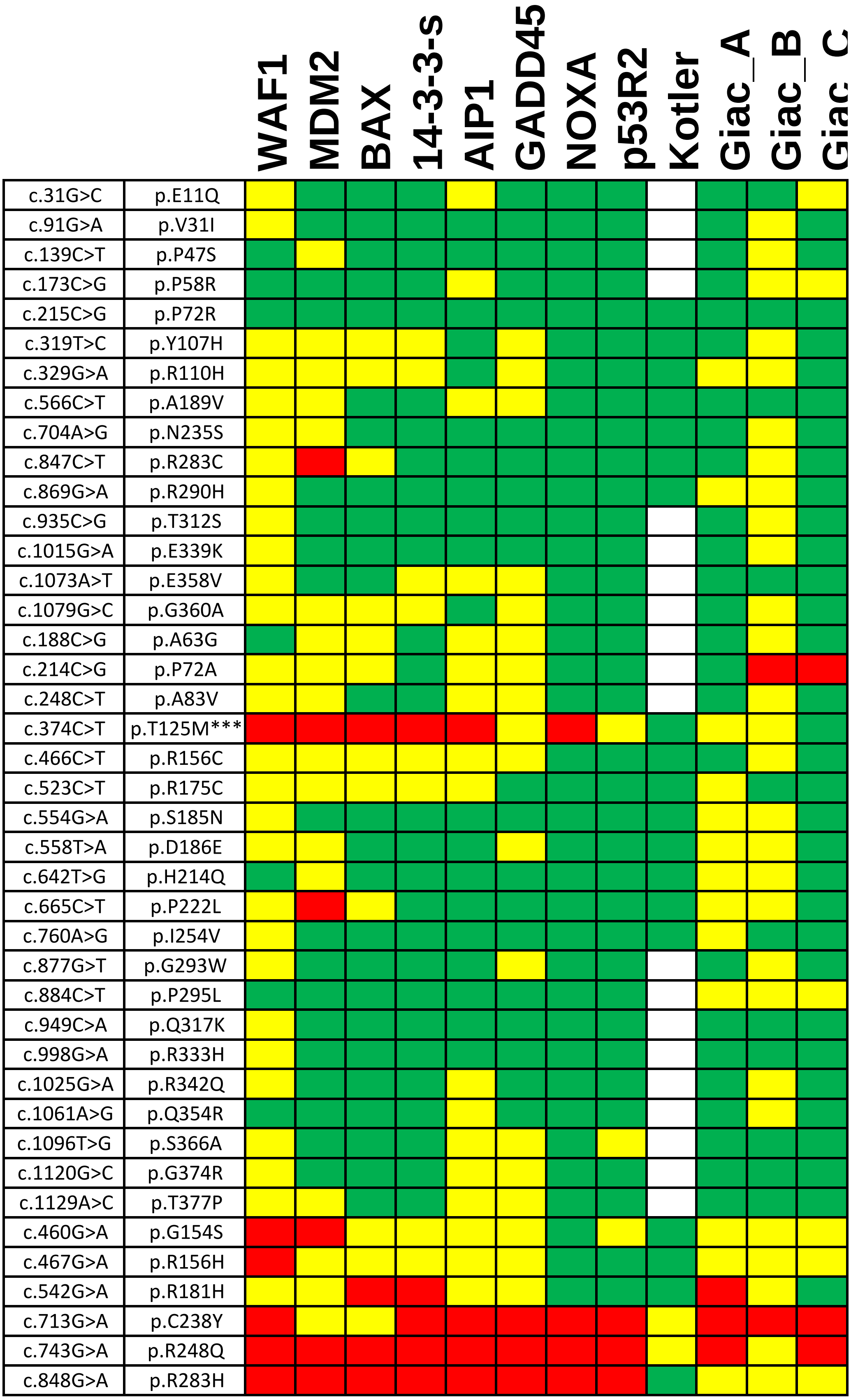

Supplementary Figure S4A

TP53 Functional analysis and ACMG Assessment

Variant Nomenclature

HG19 variant
chr17:g.7579882C>G

HG38 variant
chr17:g.7676564C>G

TP53 RefSeqGene (LRG_321)
NG_017013.2:g.15987G>C

NM_000546.6
c.31G>C

NP_000537.3
p.Glu11Gln

Mutation Consequence
Missense_variant

p.E11Q

Functional activity

Kato & al.(Yeast Assay)

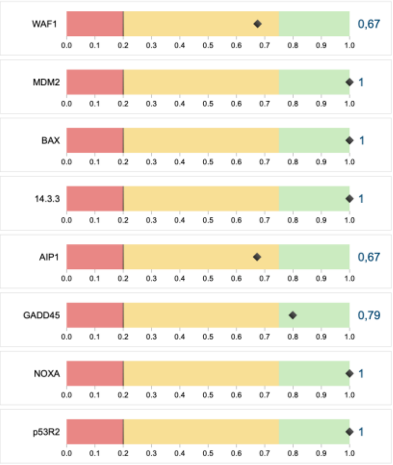

Kotler & al.(mammalian cells)

Giacomelli & Al.(mammalian cells)

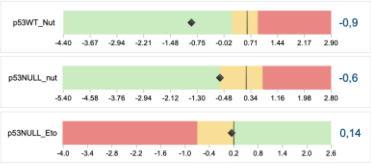

Supplementary Figure S4B

TP53 Functional analysis and ACMG Assessment

Variant Nomenclature

HG19 variant
chr17:g.7579705C>T

HG38 variant
chr17:g.7676387C>T

TP53 RefSeqGene (LRG_321)
NG_017013.2:g.16164G>A

NM_000546.6
c.91G>A

NP_000537.3
p.Val31Ile

Mutation Consequence
Missense_variant

p.V31I

Functional activity

Kato & al.(Yeast Assay)

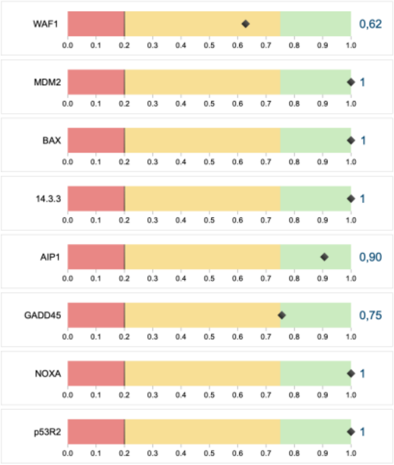

Kotler & al.(mammalian cells)

Giacomelli & Al.(mammalian cells)

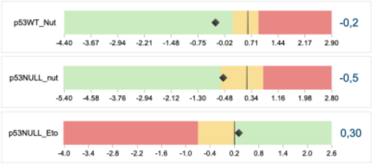

Supplementary Figure S4B

TP53 Functional analysis and ACMG Assessment

Variant Nomenclature

p.P47S

HG19 variant
chr17:g.7579548G>A

HG38 variant
chr17:g.7676230G>A

TP53 RefSeqGene (LRG_321)
NG_017013.2:g.16321C>T

NM_000546.6
c.139C>T

NP_000537.3
p.Pro47Ser

Mutation Consequence
Missense_variant

Functional activity

Kato & al.(Yeast Assay)

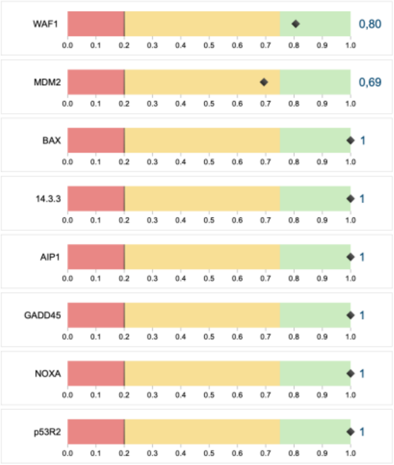

Kotler & al.(mammalian cells)

Giacomelli & Al.(mammalian cells)

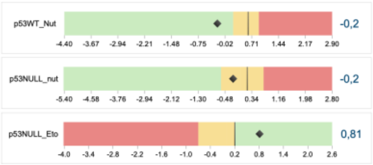

Supplementary Figure S4B

TP53 Functional analysis and ACMG Assessment

Variant Nomenclature

p.P58R

HG19 variant
chr17:g.7579514G>C

HG38 variant
chr17:g.7676196G>C

TP53 RefSeqGene (LRG_321)
NG_017013.2:g.16355C>G

NM_000546.6
c.173C>G

NP_000537.3
p.Pro58Arg

Mutation Consequence
Missense_variant

Functional activity

Kato & al.(Yeast Assay)

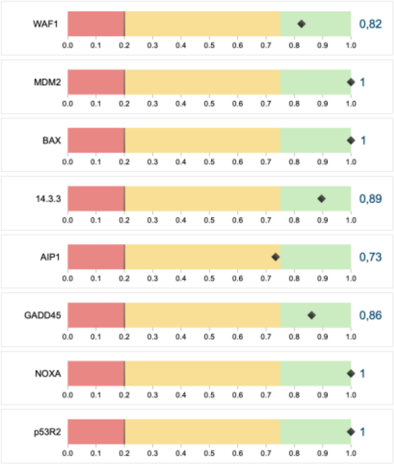

Kotler & al.(mammalian cells)

Giacomelli & Al.(mammalian cells)

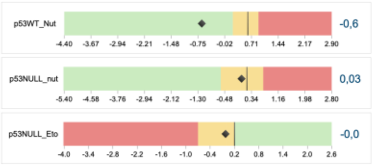

Supplementary Figure S4B

TP53 Functional analysis and ACMG Assessment

Variant Nomenclature

HG19 variant
chr17:g.7579499G>C

HG38 variant
chr17:g.7676181G>C

TP53 RefSeqGene (LRG_321)
NG_017013.2:g.16370C>G

NM_000546.6
c.188C>G

NP_000537.3
p.Ala63Gly

Mutation Consequence
Missense_variant

p.A63G

Functional activity

Kato & al.(Yeast Assay)

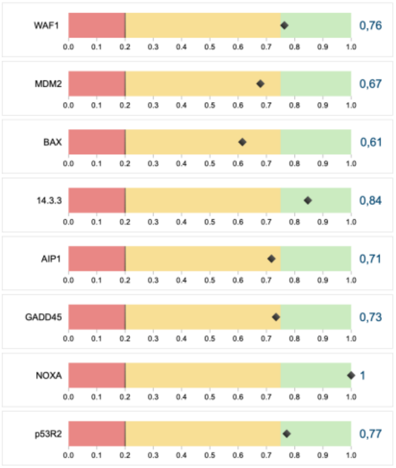

Kotler & al.(mammalian cells)

Giacomelli & Al.(mammalian cells)

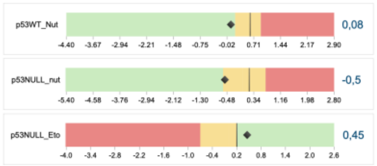

Supplementary Figure S4B

TP53 variant p.Ala63Gly: functional assessment

Variant Nomenclature

GRCh37.p13 chr 17
chr17:g.7579473G>C

GRCh38.p12 chr 17
chr17:g.7676155G>C

TP53 RefSeqGene (LRG_321)
NG_017013.2:g.16396C>G

NM_000546.6
c.214C>G

NP_000537.3
p.Pro72Ala

Mutation Consequence
Missense

p.P72A

Functional activity

Kato & al.(Yeast Assay)

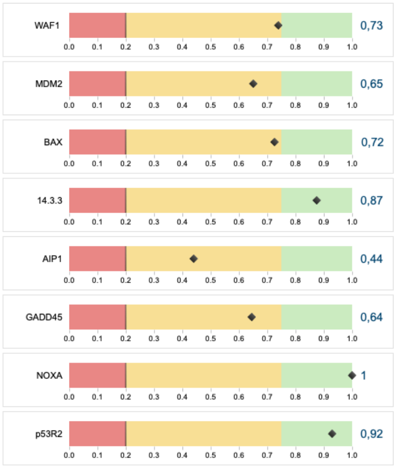

Kotler & al.(mammalian cells)

Giacomelli & Al.(mammalian cells)

Supplementary Figure S4B

TP53 Functional analysis and ACMG Assessment

Variant Nomenclature

HG19 variant
chr17:g.7579472G>C

HG38 variant
chr17:g.7676154G>C

TP53 RefSeqGene (LRG_321)
NG_017013.2:g.16397C>G

NM_000546.6
c.215C>G

NP_000537.3
p.Pro72Arg

Mutation Consequence
Missense_variant

p.P72R

Functional activity

Kato & al.(Yeast Assay)

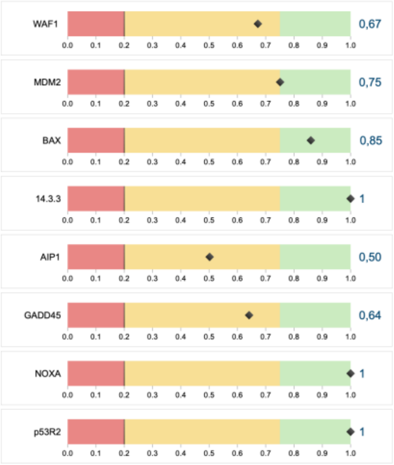

Kotler & al.(mammalian cells)

Giacomelli & Al.(mammalian cells)

Supplementary Figure S4B

TP53 variant p.Ala63Gly: functional assessment

Variant Nomenclature

GRCh37.p13 chr 17
chr17:g.7579439G>A

GRCh38.p12 chr 17
chr17:g.7676121G>A

TP53 RefSeqGene (LRG_321)
NG_017013.2:g.16430C>T

NM_000546.6
c.248C>T

NP_000537.3
p.Ala83Val

Mutation Consequence
Missense

p.A83V

Functional activity

Kato & al.(Yeast Assay)

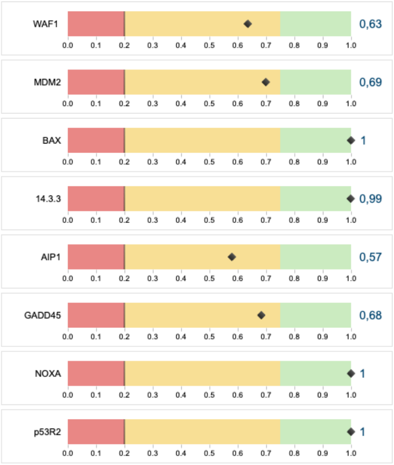

Kotler & al.(mammalian cells)

Giacomelli & Al.(mammalian cells)

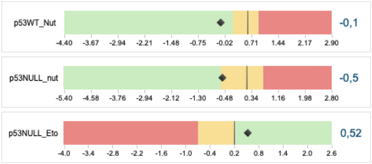

Supplementary Figure S4B

TP53 Functional analysis and ACMG Assessment

Variant Nomenclature

HG19 variant
chr17:g.7579368A>G

HG38 variant
chr17:g.7676050A>G

TP53 RefSeqGene (LRG_321)
NG_017013.2:g.16501T>C

NM_000546.6
c.319T>C

NP_000537.3
p.Tyr107His

Mutation Consequence
Missense_variant

p.Y107H

Functional activity

Kato & al.(Yeast Assay)

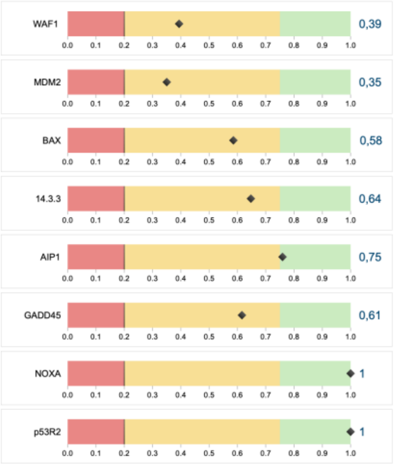

Kotler & al.(mammalian cells)

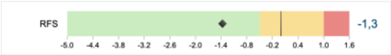

Giacomelli & Al.(mammalian cells)

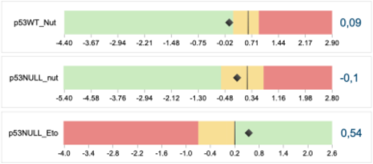

Supplementary Figure S4B

TP53 Functional analysis and ACMG Assessment

Variant Nomenclature

HG19 variant
chr17:g.7579358C>T

HG38 variant
chr17:g.7676040C>T

TP53 RefSeqGene (LRG_321)
NG_017013.2:g.16511G>A

NM_000546.6
c.329G>A

NP_000537.3
p.Arg110His

Mutation Consequence
Missense_variant

p.R110H

Functional activity

Kato & al.(Yeast Assay)

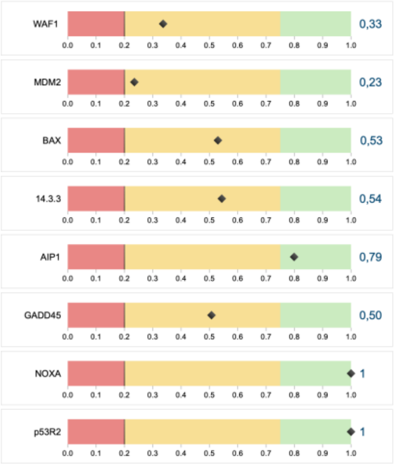

Kotler & al.(mammalian cells)

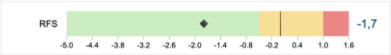

Giacomelli & Al.(mammalian cells)

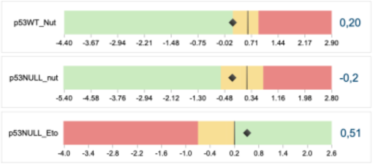

Supplementary Figure S4B

TP53 variant p.Ala63Gly: functional assessment

Variant Nomenclature

GRCh37.p13 chr 17
chr17:g.7579313G>A

GRCh38.p12 chr 17
chr17:g.7675995G>A

TP53 RefSeqGene (LRG_321)
NG_017013.2:g.16556C>T

NM_000546.6
c.374C>T

NP_000537.3
p.Thr125Met

Mutation Consequence
Missense

p.T125M

Functional activity

Kato & al.(Yeast Assay)

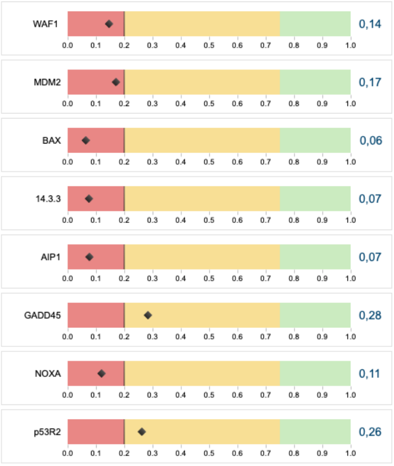

Kotler & al.(mammalian cells)

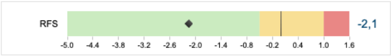

Giacomelli & Al.(mammalian cells)

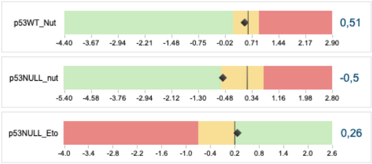

Supplementary Figure S4B

TP53 Functional analysis and ACMG Assessment

Variant Nomenclature

GRCh37.p13 chr 17
chr17:g.7578470C>T

GRCh38.p12 chr 17
chr17:g.7675152C>T

TP53 RefSeqGene (LRG_321)
NG_017013.2:g.17399G>A

NM_000546.6
c.460G>A

NP_000537.3
p.Gly154Ser

Mutation Consequence
Missense

p.G154S

Functional activity

Kato & al.(Yeast Assay)

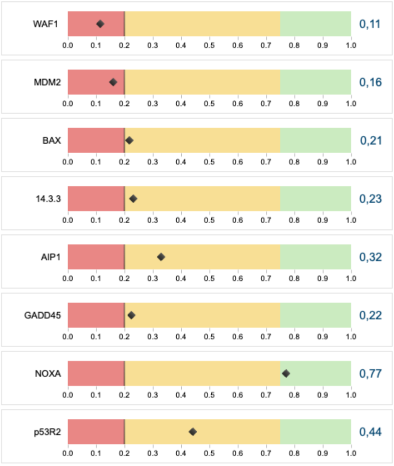

Kotler & al.(mammalian cells)

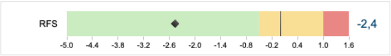

Giacomelli & Al.(mammalian cells)

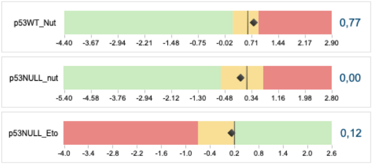

Supplementary Figure S4B

TP53 variant p.Ala63Gly: functional assessment

Variant Nomenclature

GRCh37.p13 chr 17
chr17:g.7578464G>A
GRCh38.p12 chr 17
chr17:g.7675146G>A
TP53 RefSeqGene (LRG_321)
NG_017013.2:g.17405C>T
NM_000546.6
c.466C>T
NP_000537.3
p.Arg156Cys
Mutation Consequence
Missense

p.R156C

Functional activity

Kato & al.(Yeast Assay)

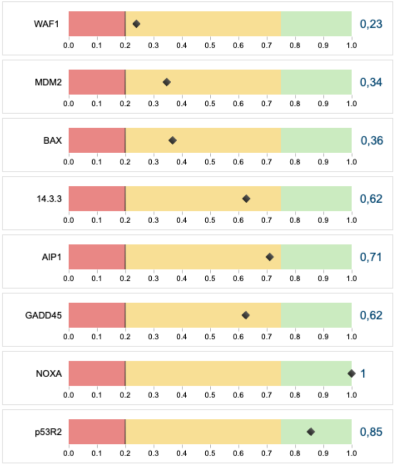

Kotler & al.(mammalian cells)

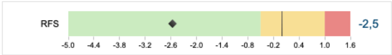

Giacomelli & Al.(mammalian cells)

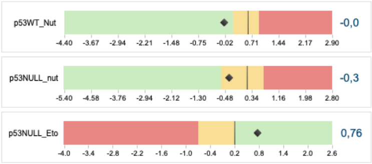

Supplementary Figure S4B

TP53 Functional analysis and ACMG Assessment

Variant Nomenclature

GRCh37.p13 chr 17
chr17:g.7578463C>T
GRCh38.p12 chr 17
chr17:g.7675145C>T
TP53 RefSeqGene (LRG_321)
NG_017013.2:g.17406G>A
NM_000546.6
c.467G>A
NP_000537.3
p.Arg156His
Mutation Consequence
Missense

p.R156H

Functional activity

Kato & al.(Yeast Assay)

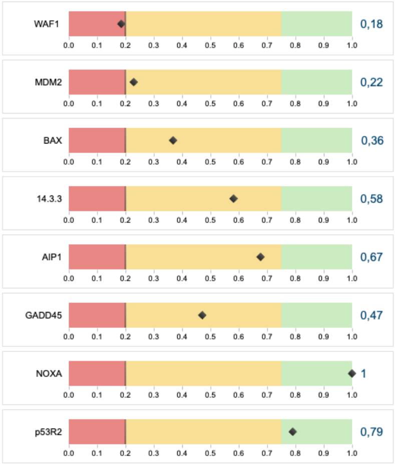

Kotler & al.(mammalian cells)

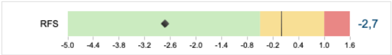

Giacomelli & Al.(mammalian cells)

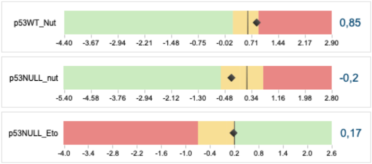

Supplementary Figure S4B

TP53 variant p.Ala63Gly: functional assessment

Variant Nomenclature

GRCh37.p13 chr 17
chr17:g.7578407G>A

GRCh38.p12 chr 17
chr17:g.7675089G>A

TP53 RefSeqGene (LRG_321)
NG_017013.2:g.17462C>T

NM_000546.6
c.523C>T

NP_000537.3
p.Arg175Cys

Mutation Consequence
Missense

p.R175C

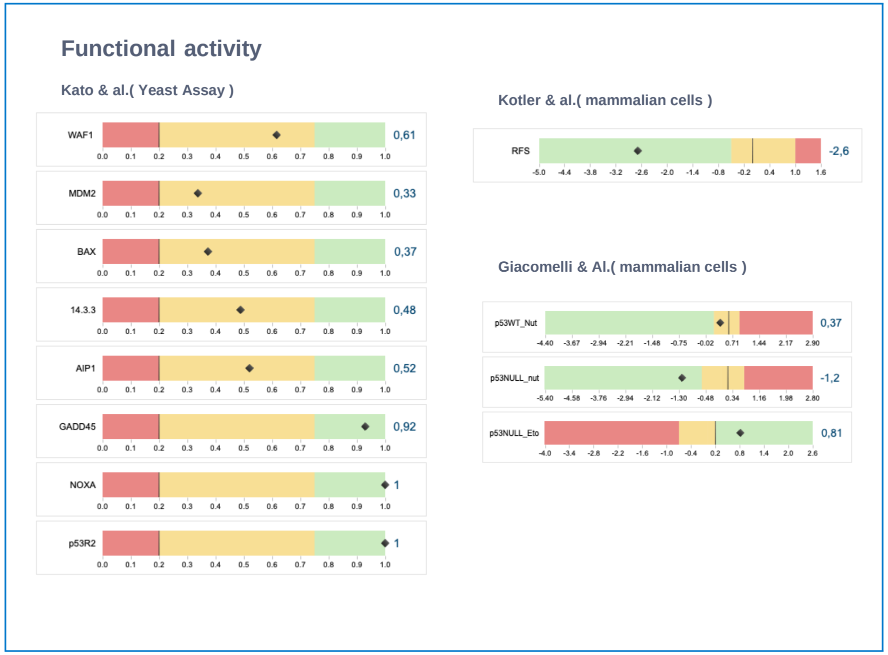

Supplementary Figure S4B

TP53 Functional analysis and ACMG Assessment

Variant Nomenclature

GRCh37.p13 chr 17
chr17:g.7578388C>T

GRCh38.p12 chr 17
chr17:g.7675070C>T

TP53 RefSeqGene (LRG_321)
NG_017013.2:g.17481G>A

NM_000546.6
c.542G>A

NP_000537.3
p.Arg181His

Mutation Consequence
Missense

p.R181H

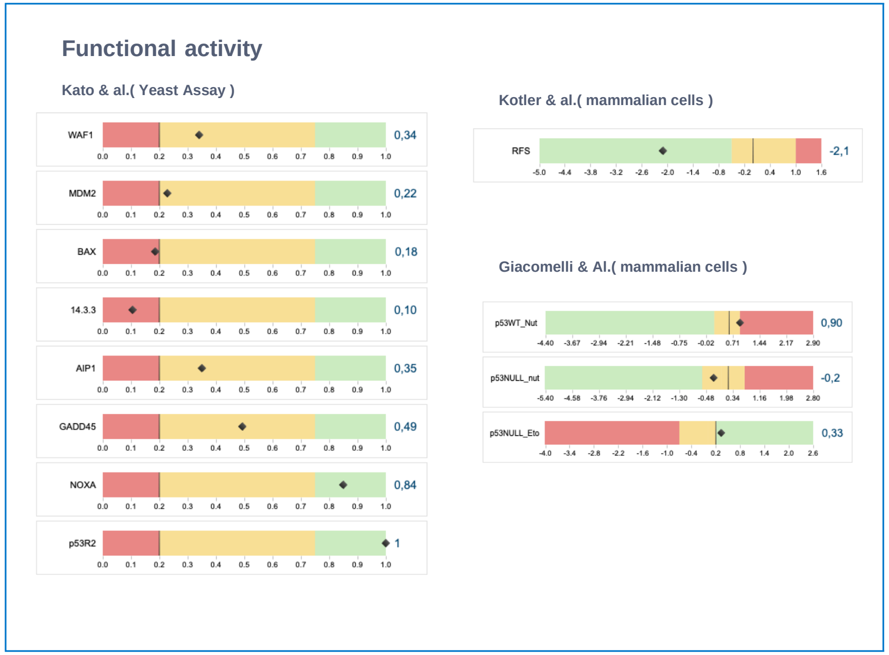

Supplementary Figure S4B

TP53 variant p.Ala63Gly: functional assessment

Variant Nomenclature

GRCh37.p13 chr 17

chr17:g.7578376C>T

GRCh38.p12 chr 17

chr17:g.7675058C>T

TP53 RefSeqGene (LRG_321)

NG_017013.2:g.17493G>A

NM_000546.6

c.554G>A

NP_000537.3

p.Ser185Asn

Mutation Consequence

Missense

p.R185N

Functional activity

Kato & al.(Yeast Assay)

Kotler & al.(mammalian cells)

Giacomelli & Al.(mammalian cells)

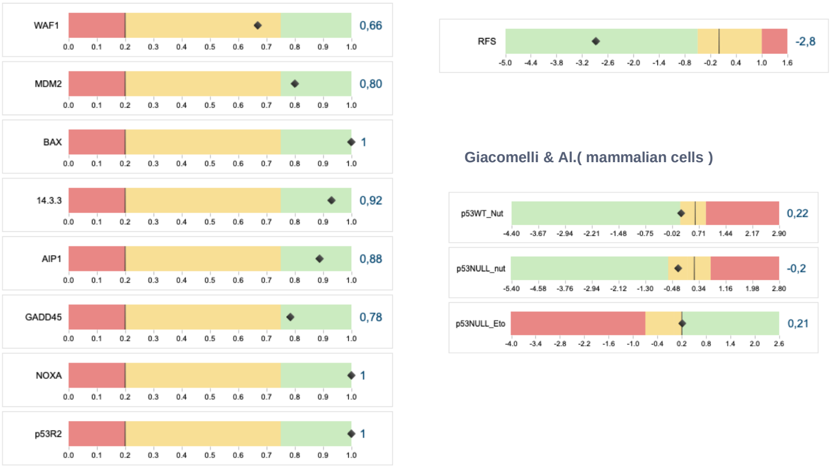

Supplementary Figure S4B

TP53 variant p.Ala63Gly: functional assessment

Variant Nomenclature

GRCh37.p13 chr 17

chr17:g.7578372A>T

GRCh38.p12 chr 17

chr17:g.7675054A>T

TP53 RefSeqGene (LRG_321)

NG_017013.2:g.17497T>A

NM_000546.6

c.558T>A

NP_000537.3

p.Asp186Glu

Mutation Consequence

Missense

p.D186E

Functional activity

Kato & al.(Yeast Assay)

Kotler & al.(mammalian cells)

Giacomelli & Al.(mammalian cells)

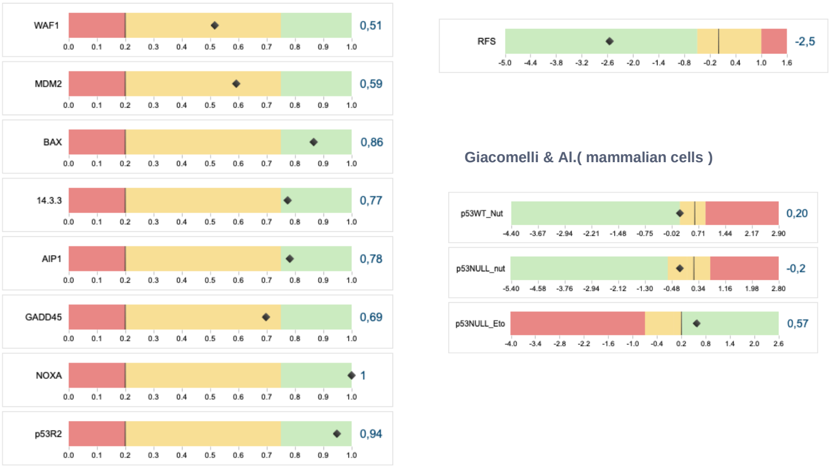

Supplementary Figure S4B

TP53 Functional analysis and ACMG Assessment

Variant Nomenclature

HG19 variant
chr17:g.7578283G>A

HG38 variant
chr17:g.7674965G>A

TP53 RefSeqGene (LRG_321)
NG_017013.2:g.17586C>T

NM_000546.6
c.566C>T

NP_000537.3
p.Ala189Val

Mutation Consequence
Missense_variant

p.A189V

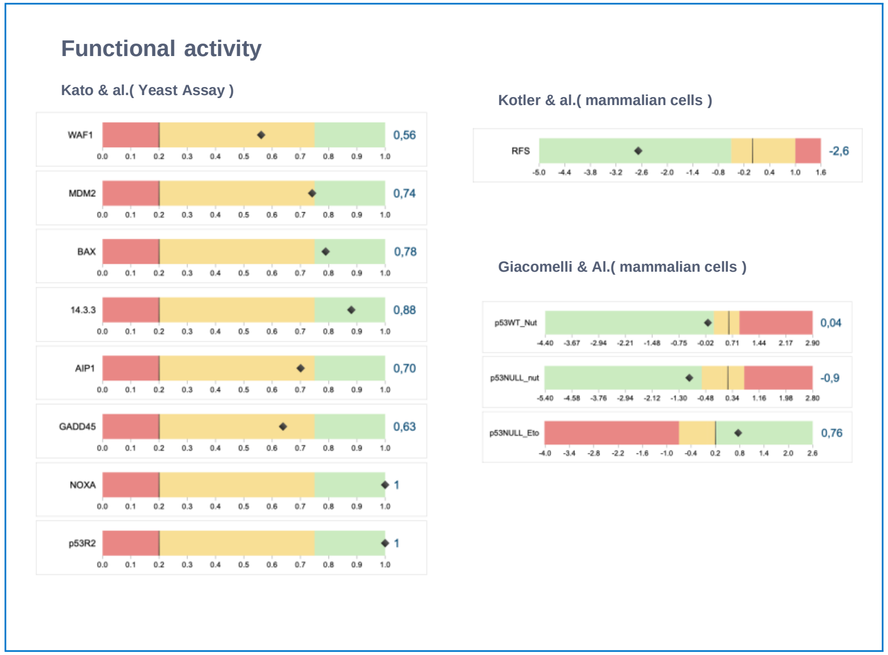

Supplementary Figure S4B

TP53 variant p.Ala63Gly: functional assessment

Variant Nomenclature

GRCh37.p13 chr 17
chr17:g.7578207A>C

GRCh38.p12 chr 17
chr17:g.7674889A>C

TP53 RefSeqGene (LRG_321)
NG_017013.2:g.17662T>G

NM_000546.6
c.642T>G

NP_000537.3
p.His214Gln

Mutation Consequence
Missense

p.H214Q

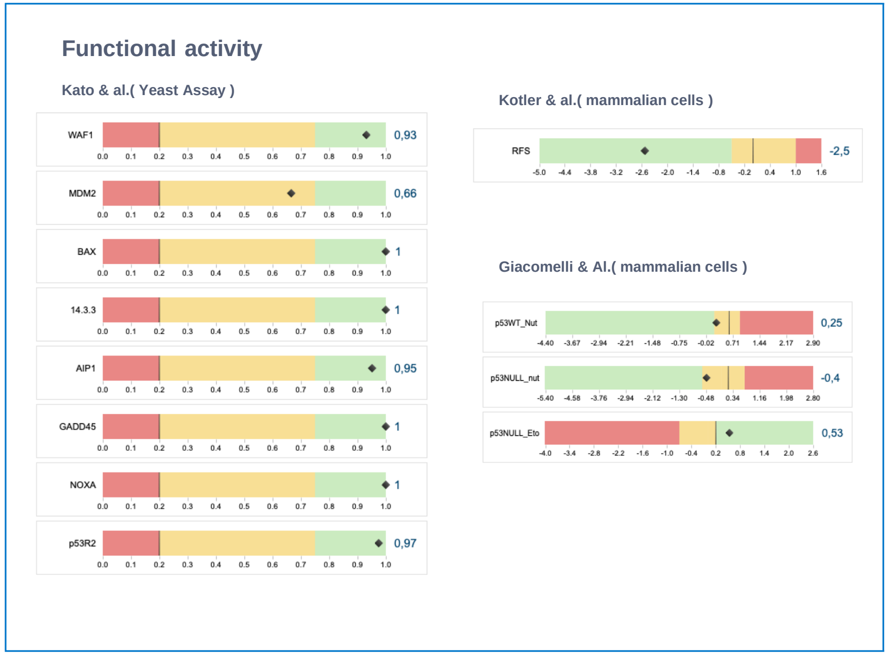

Supplementary Figure S4B

TP53 Functional analysis and ACMG Assessment

Variant Nomenclature

HG19 variant
chr17:g.7578184G>A

HG38 variant
chr17:g.7674866G>A

TP53 RefSeqGene (LRG_321)
NG_017013.2:g.17685C>T

NM_000546.6
c.665C>T

NP_000537.3
p.Pro222Leu

Mutation Consequence
Missense_variant

p.P222L

Functional activity

Kato & al.(Yeast Assay)

Kotler & al.(mammalian cells)

Giacomelli & Al.(mammalian cells)

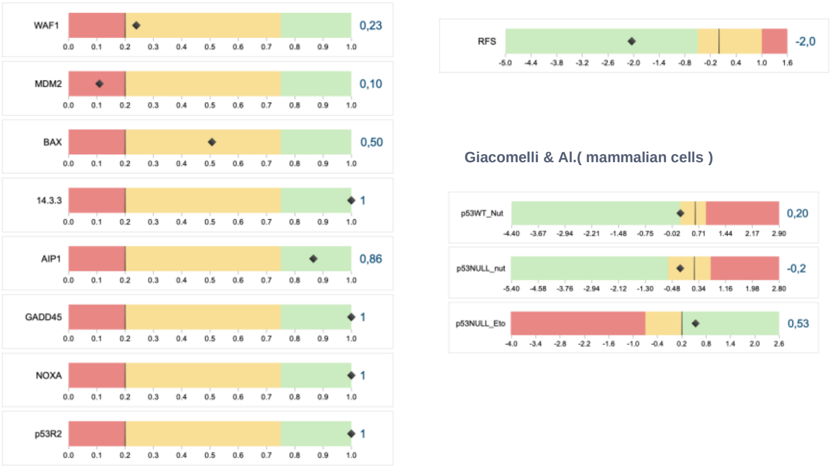

Supplementary Figure S4B

TP53 Functional analysis and ACMG Assessment

Variant Nomenclature

GRCh37.p13 chr 17
chr17:g.7577577T>C

GRCh38.p12 chr 17
chr17:g.7674259T>C

TP53 RefSeqGene (LRG_321)
NG_017013.2:g.18292A>G

NM_000546.6
c.704A>G

NP_000537.3
p.Asn235Ser

Mutation Consequence
Missense

p.P235S

Functional activity

Kato & al.(Yeast Assay)

Kotler & al.(mammalian cells)

Giacomelli & Al.(mammalian cells)

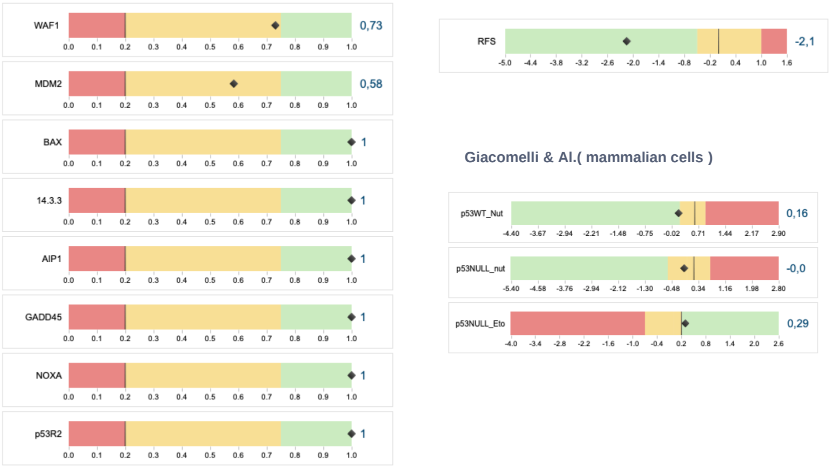

Supplementary Figure S4B

TP53 Functional analysis and ACMG Assessment

Variant Nomenclature

GRCh37.p13 chr 17	chr17:g.7577568C>T
GRCh38.p12 chr 17	chr17:g.7674250C>T
TP53 RefSeqGene (LRG_321)	NG_017013.2:g.18301G>A
NM_000546.6	c.713G>A
NP_000537.3	p.Cys238Tyr
Mutation Consequence	Missense

p.C238Y

Functional activity

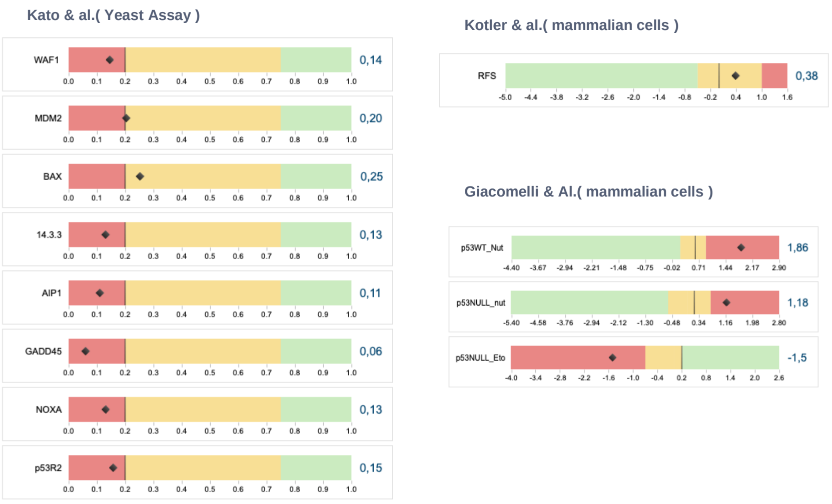

Supplementary Figure S4B

TP53 Functional analysis and ACMG Assessment

Variant Nomenclature

GRCh37.p13 chr 17	chr17:g.7577538C>T
GRCh38.p12 chr 17	chr17:g.7674220C>T
TP53 RefSeqGene (LRG_321)	NG_017013.2:g.18331G>A
NM_000546.6	c.743G>A
NP_000537.3	p.Arg248Gln
Mutation Consequence	Missense

p.R248Q

Functional activity

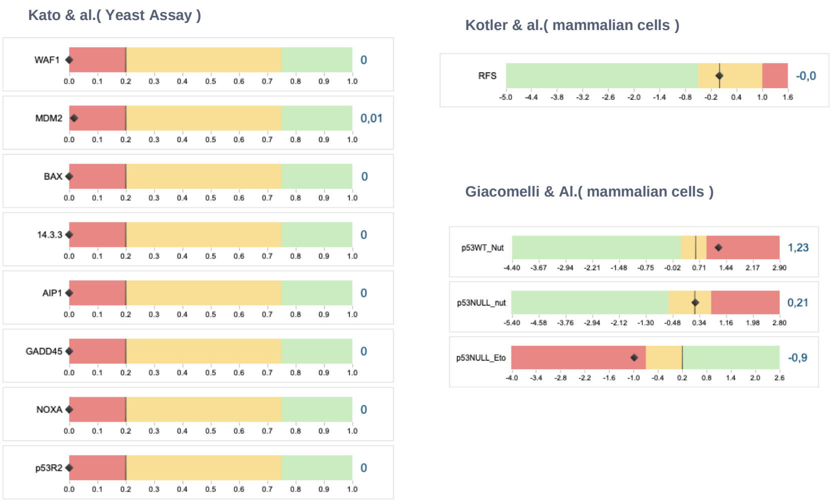

Supplementary Figure S4B

TP53 Functional analysis and ACMG Assessment

Variant Nomenclature

GRCh37.p13 chr 17
chr17:g.7577521T>C

GRCh38.p12 chr 17
chr17:g.7674203T>C

TP53 RefSeqGene (LRG_321)
NG_017013.2:g.18348A>G

NM_000546.6
c.760A>G

NP_000537.3
p.Ile254Val

Mutation Consequence
Missense

p.I254V

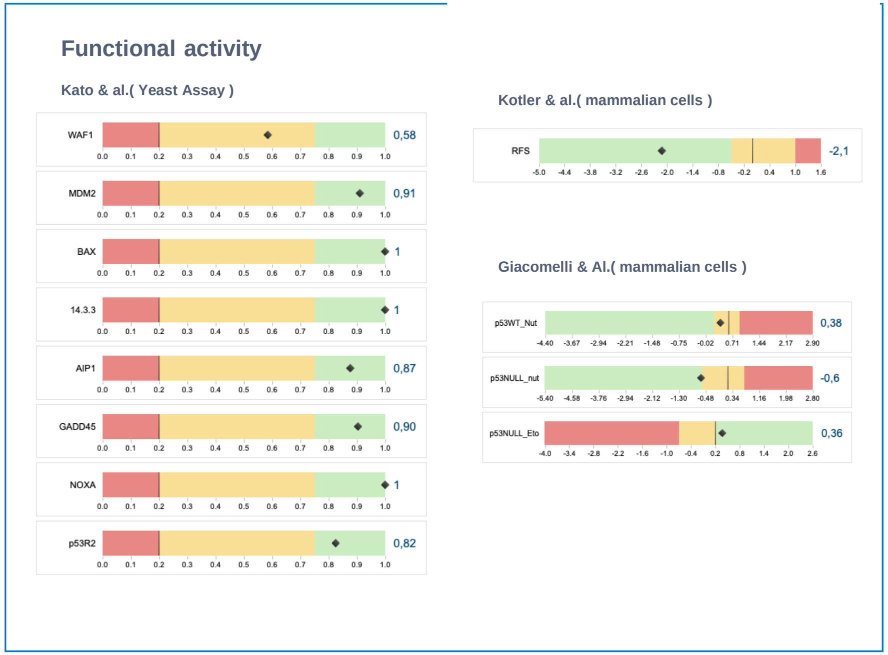

Supplementary Figure S4B

TP53 Functional analysis and ACMG Assessment

Variant Nomenclature

GRCh37.p13 chr 17
chr17:g.7577091G>A

GRCh38.p12 chr 17
chr17:g.7673773G>A

TP53 RefSeqGene (LRG_321)
NG_017013.2:g.18778C>T

NM_000546.6
c.847C>T

NP_000537.3
p.Arg283Cys

Mutation Consequence
Missense

p.R283C

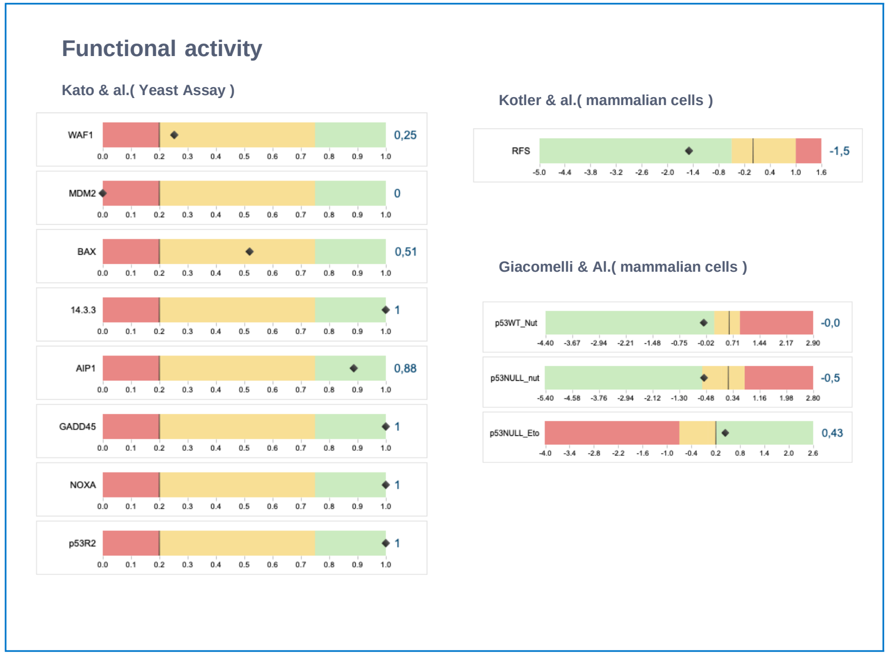

Supplementary Figure S4B

TP53 Functional analysis and ACMG Assessment

Variant Nomenclature

GRCh37.p13 chr 17

chr17:g.7577090C>T

GRCh38.p12 chr 17

chr17:g.7673772C>T

TP53 RefSeqGene (LRG_321)

NG_017013.2:g.18779G>A

NM_000546.6

c.848G>A

NP_000537.3

p.Arg283His

Mutation Consequence

Missense

p.R283H

Functional activity

Kato & al.(Yeast Assay)

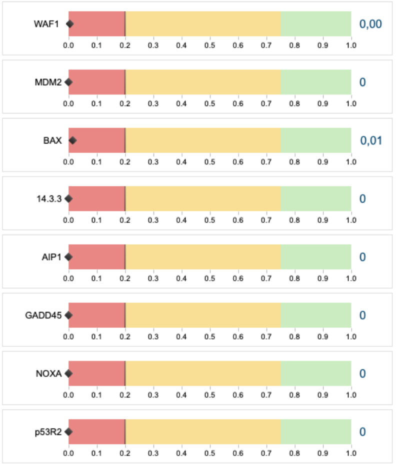

Kotler & al.(mammalian cells)

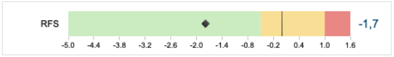

Giacomelli & Al.(mammalian cells)

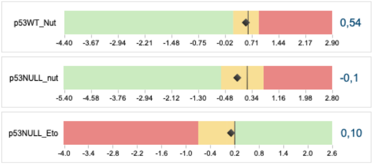

Supplementary Figure S4B

TP53 Functional analysis and ACMG Assessment

Variant Nomenclature

GRCh37.p13 chr 17

chr17:g.7577069C>T

GRCh38.p12 chr 17

chr17:g.7673751C>T

TP53 RefSeqGene (LRG_321)

NG_017013.2:g.18800G>A

NM_000546.6

c.869G>A

NP_000537.3

p.Arg290His

Mutation Consequence

Missense

p.R290H

Functional activity

Kato & al.(Yeast Assay)

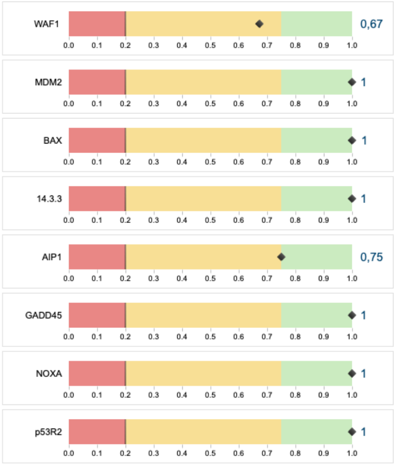

Kotler & al.(mammalian cells)

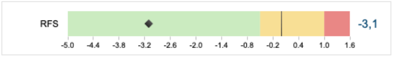

Giacomelli & Al.(mammalian cells)

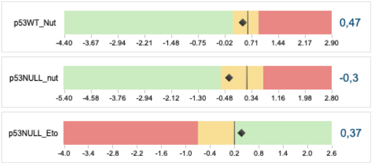

Supplementary Figure S4B

TP53 variant p.Ala63Gly: functional assessment

Variant Nomenclature

GRCh37.p13 chr 17
chr17:g.7577061C>A

GRCh38.p12 chr 17
chr17:g.7673743C>A

TP53 RefSeqGene (LRG_321)
NG_017013.2:g.18808G>T

NM_000546.6
c.877G>T

NP_000537.3
p.Gly293Trp

Mutation Consequence
Missense

p.G293W

Functional activity

Kato & al.(Yeast Assay)

Kotler & al.(mammalian cells)

Giacomelli & Al.(mammalian cells)

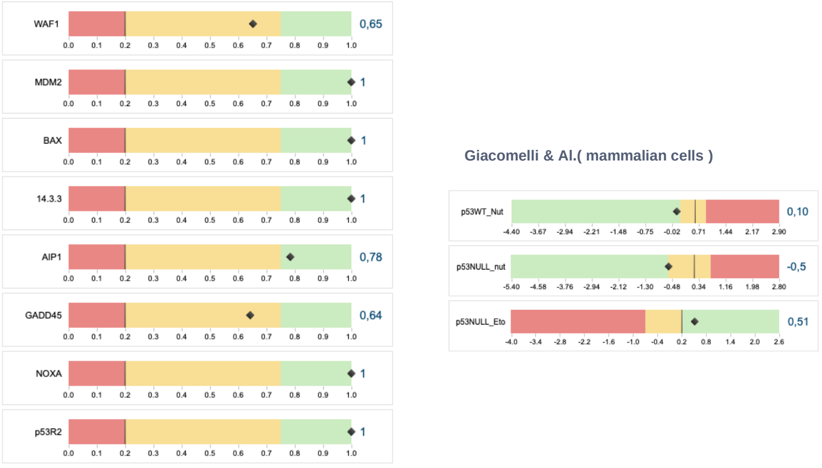

Supplementary Figure S4B

TP53 variant p.Ala63Gly: functional assessment

Variant Nomenclature

GRCh37.p13 chr 17
chr17:g.7577054G>A

GRCh38.p12 chr 17
chr17:g.7673736G>A

TP53 RefSeqGene (LRG_321)
NG_017013.2:g.18815C>T

NM_000546.6
c.884C>T

NP_000537.3
p.Pro295Leu

Mutation Consequence
Missense

p.P295L

Functional activity

Kato & al.(Yeast Assay)

Kotler & al.(mammalian cells)

Giacomelli & Al.(mammalian cells)

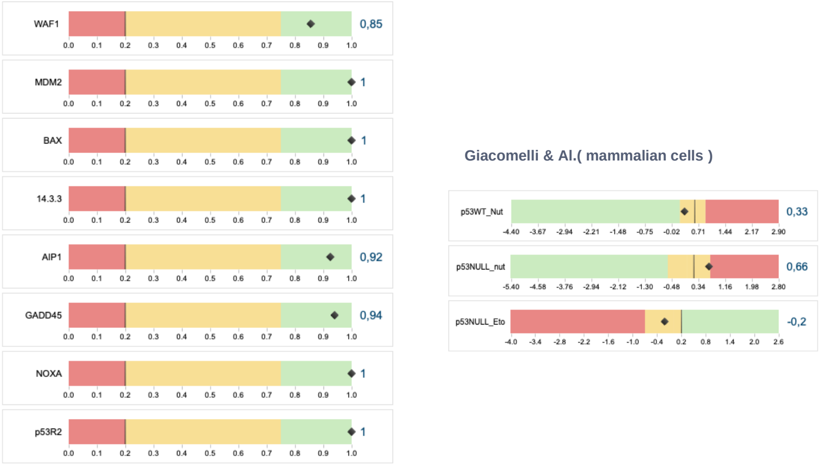

Supplementary Figure S4B

TP53 Functional analysis and ACMG Assessment

Variant Nomenclature

HG19 variant
chr17:g.7576911G>C

HG38 variant
chr17:g.7673593G>C

TP53 RefSeqGene (LRG_321)
NG_017013.2:g.18958C>G

NM_000546.6
c.935C>G

NP_000537.3
p.Thr312Ser

Mutation Consequence
Missense_variant

p.T312S

Functional activity

Kato & al.(Yeast Assay)

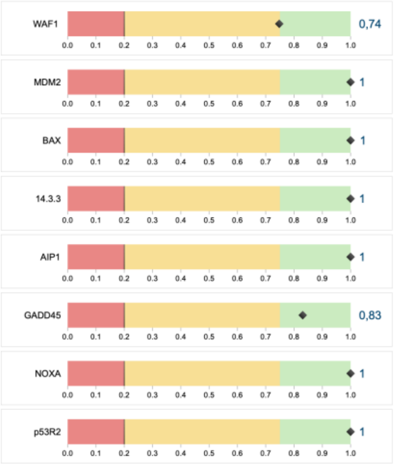

Kotler & al.(mammalian cells)

Giacomelli & Al.(mammalian cells)

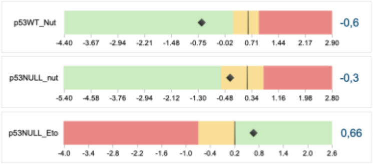

Supplementary Figure S4B

TP53 variant p.Ala63Gly: functional assessment

Variant Nomenclature

GRCh37.p13 chr 17
chr17:g.7576897G>T

GRCh38.p12 chr 17
chr17:g.7673579G>T

TP53 RefSeqGene (LRG_321)
NG_017013.2:g.18972C>A

NM_000546.6
c.949C>A

NP_000537.3
p.Gln317Lys

Mutation Consequence
Missense

p.Q317K

Functional activity

Kato & al.(Yeast Assay)

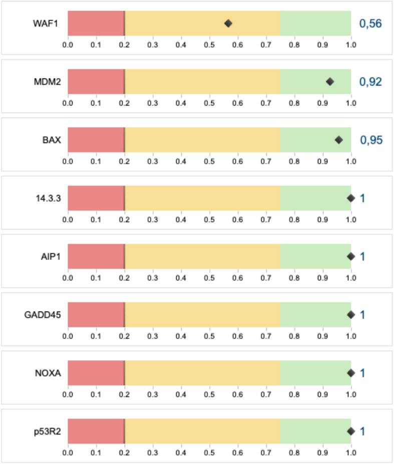

Kotler & al.(mammalian cells)

Giacomelli & Al.(mammalian cells)

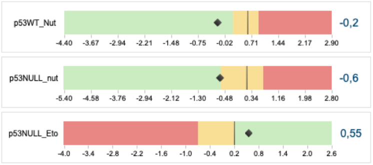

Supplementary Figure S4B

TP53 Functional analysis and ACMG Assessment

Variant Nomenclature

GRCh37.p13 chr 17
chr17:g.7574029C>T

GRCh38.p12 chr 17
chr17:g.7670711C>T

TP53 RefSeqGene (LRG_321)
NG_017013.2:g.21840G>A

NM_000546.6
c.998G>A

NP_000537.3
p.Arg333His

Mutation Consequence
Missense

p.R333H

Functional activity

Kato & al.(Yeast Assay)

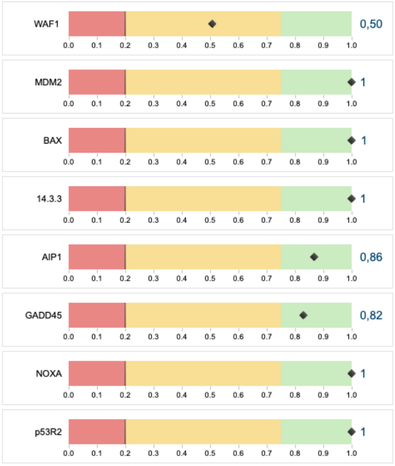

Kotler & al.(mammalian cells)

Giacomelli & Al.(mammalian cells)

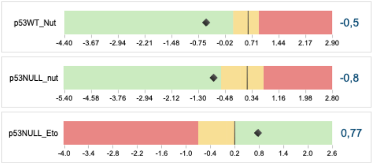

Supplementary Figure S4B

TP53 Functional analysis and ACMG Assessment

Variant Nomenclature

HG19 variant
chr17:g.7574012C>T

HG38 variant
chr17:g.7670694C>T

TP53 RefSeqGene (LRG_321)
NG_017013.2:g.21857G>A

NM_000546.6
c.1015G>A

NP_000537.3
p.Glu339Lys

Mutation Consequence
Missense_variant

p.E339K

Functional activity

Kato & al.(Yeast Assay)

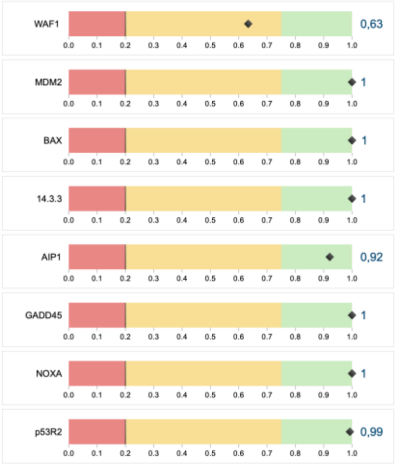

Kotler & al.(mammalian cells)

Giacomelli & Al.(mammalian cells)

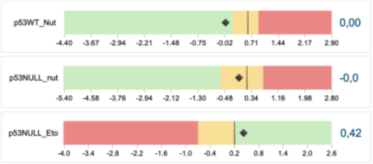

Supplementary Figure S4B

TP53 variant p.Ala63Gly: functional assessment

Variant Nomenclature

GRCh37.p13 chr 17

chr17:g.7574002C>T

GRCh38.p12 chr 17

chr17:g.7670684C>T

TP53 RefSeqGene (LRG_321)

NG_017013.2:g.21867G>A

NM_000546.6

c.1025G>A

NP_000537.3

p.Arg342Gln

Mutation Consequence

Missense

p.R342Q

Functional activity

Kato & al.(Yeast Assay)

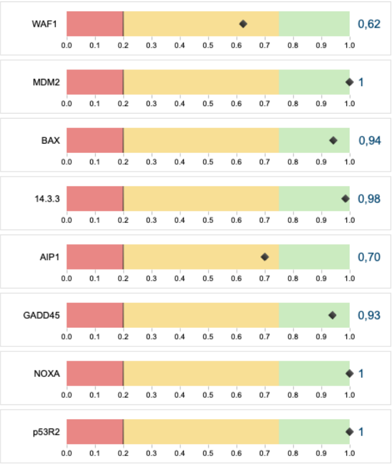

Kotler & al.(mammalian cells)

Giacomelli & Al.(mammalian cells)

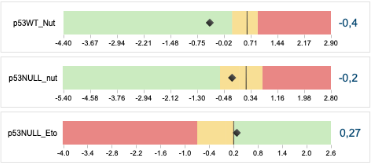

Supplementary Figure S4B

TP53 variant p.Ala63Gly: functional assessment

Variant Nomenclature

GRCh37.p13 chr 17

chr17:g.7573966T>C

GRCh38.p12 chr 17

chr17:g.7670648T>C

TP53 RefSeqGene (LRG_321)

NG_017013.2:g.21903A>G

NM_000546.6

c.1061A>G

NP_000537.3

p.Gln354Arg

Mutation Consequence

Missense

p.Q354R

Functional activity

Kato & al.(Yeast Assay)

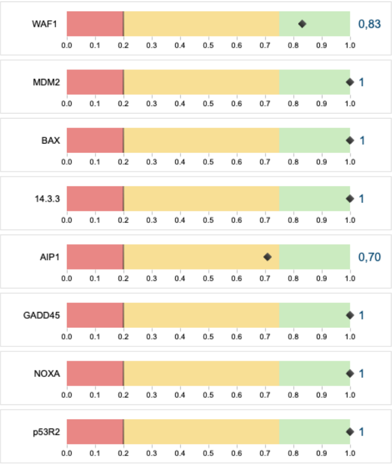

Kotler & al.(mammalian cells)

Giacomelli & Al.(mammalian cells)

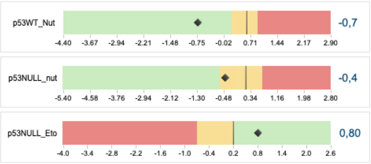

Supplementary Figure S4B

TP53 Functional analysis and ACMG Assessment

Variant Nomenclature

HG19 variant
chr17:g.7573954T>A

HG38 variant
chr17:g.7670636T>A

TP53 RefSeqGene (LRG_321)
NG_017013.2:g.21915A>T

NM_000546.6
c.1073A>T

NP_000537.3
p.Glu358Val

Mutation Consequence
Missense_variant

p.G358V

Functional activity

Kato & al.(Yeast Assay)

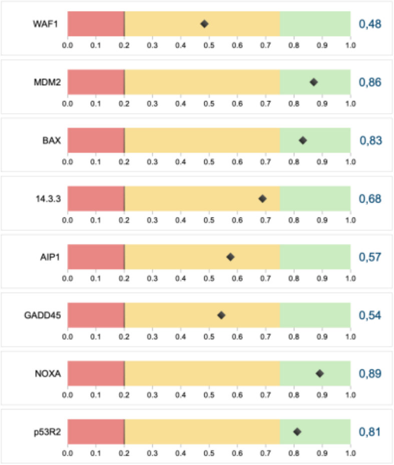

Kotler & al.(mammalian cells)

Giacomelli & Al.(mammalian cells)

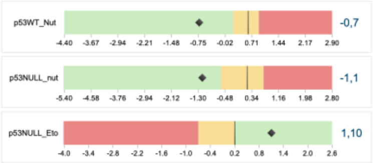

Supplementary Figure S4B

TP53 Functional analysis and ACMG Assessment

Variant Nomenclature

HG19 variant
chr17:g.7573948C>G

HG38 variant
chr17:g.7670630C>G

TP53 RefSeqGene (LRG_321)
NG_017013.2:g.21921G>C

NM_000546.6
c.1079G>C

NP_000537.3
p.Gly360Ala

Mutation Consequence
Missense_variant

p.G360A

Functional activity

Kato & al.(Yeast Assay)

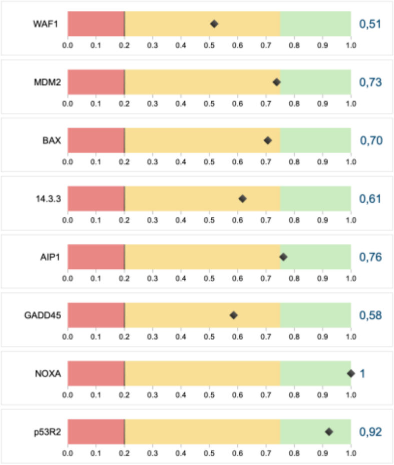

Kotler & al.(mammalian cells)

Giacomelli & Al.(mammalian cells)

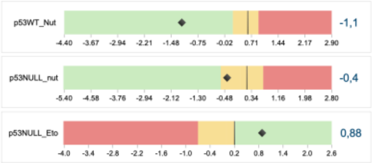

Supplementary Figure S4B

TP53 variant p.Ala63Gly: functional assessment

Variant Nomenclature

GRCh37.p13 chr 17	chr17:g.7573931A>C
GRCh38.p12 chr 17	chr17:g.7670613A>C
TP53 RefSeqGene (LRG_321)	NG_017013.2:g.21938T>G
NM_000546.6	c.1096T>G
NP_000537.3	p.Ser366Ala
Mutation Consequence	Missense

p.S366A

Functional activity

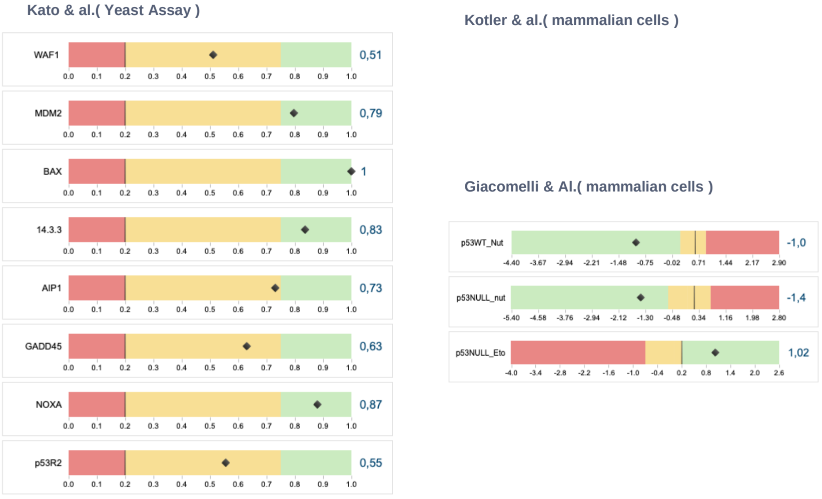

Supplementary Figure S4B

TP53 variant p.Ala63Gly: functional assessment

Variant Nomenclature

GRCh37.p13 chr 17	chr17:g.7572989C>G
GRCh38.p12 chr 17	chr17:g.7669671C>G
TP53 RefSeqGene (LRG_321)	NG_017013.2:g.22880G>C
NM_000546.6	c.1120G>C
NP_000537.3	p.Gly374Arg
Mutation Consequence	Missense

p.G374R

Functional activity

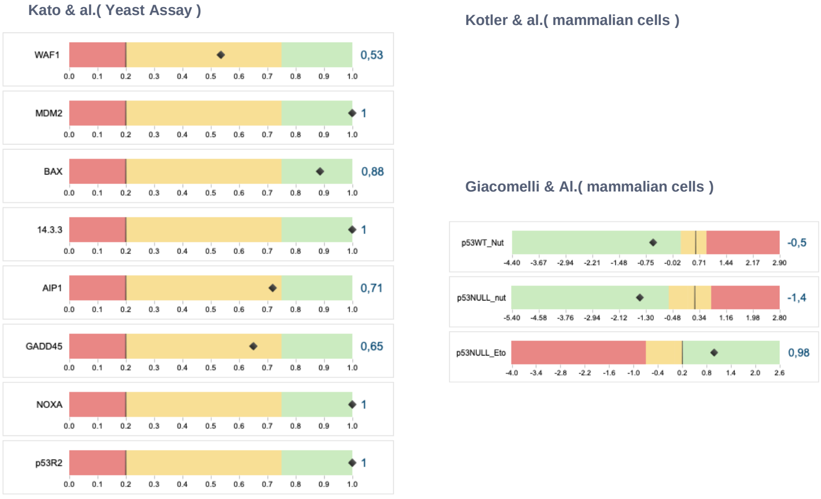

Supplementary Figure S4B

TP53 variant p.Ala63Gly: functional assessment

Variant Nomenclature

GRCh37.p13 chr 17

chr17:g.7572980T>G

GRCh38.p12 chr 17

chr17:g.7669662T>G

TP53 RefSeqGene (LRG_321)

NG_017013.2:g.22889A>C

NM_000546.6

c.1129A>C

NP_000537.3

p.Thr377Pro

Mutation Consequence

Missense

p.T377P

Functional activity

Kato & al.(Yeast Assay)

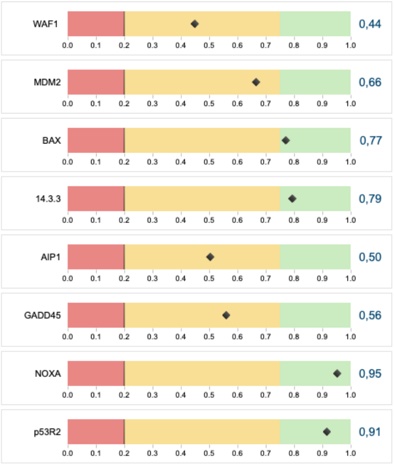

Kotler & al.(mammalian cells)

Giacomelli & Al.(mammalian cells)

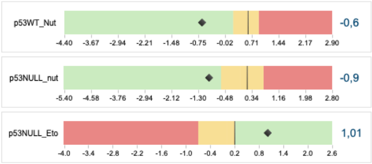

Supplementary Figure S4B
