# Supplementary material for: TP53_PROF: a machine learning model to predict impact of missense mutations in TP53
Source: Brief Bioinform. 2022 Jan 18;23(2):bbab524. doi: 10.1093/bib/bbab524 (PMC8921628; doi:10.1093/bib/bbab524)
Supplement: TP53_variants_update_bbab524 [file tp53_variants_update_bbab524.docx]

**TP53 variants summary**

**Figure:** localization of TP53 variants with unusual properties.

The intron/exon structure of the gene (coding exons 2 to 11, grey boxes) is presented at the top and structural domains of the protein are depicted below (green).

TP53 benign SNP are shown in blue.

The four variants localized in the last codon of exon 5 6 and 9 (p.T125=,p.T125M, p.E224D and p.Q331H) have been repeatedly shown to impair TP53 splicing (see below).

Other variants relevant to the study are indicated below the TP53 protein

Properties of each variant are fully described in this document.

Domain organization of the TP53 protein: TAD: transactivation domain; PRD: proline riche domain; OD: tetramerization domain; RD: regulatory domain.

**c.672G>T or c.672G>C p.(Glu224Asp)**

| **Occurrence in UMD** | **Occurrence in TCGA** | **Occurrence in GENIE** | **Occurrence in ICGC** | **Occurrence in MSKSCC** | **Occurrence in COSMIC** | **Occurrence in gnomAD** |
| --- | --- | --- | --- | --- | --- | --- |
| **212** | **5** | **26** | **2** | **18** | **34** | **4 10-^6^ *** |

*p.Glu224Asp

Last base exon nucleotide 672 has been found to be mutated in various types of cancer with multiple nucleotides substitutions leading either to a synonymous variant (G>A) or to a conservative amino acid substitution (G>T and G>C)

The three variants have been predicted to impair splicing

The three variants have been shown to be associated with intron retention in various tumors

[https://validsplicemut.cytognomix.com](https://validsplicemut.cytognomix.com/)/

**This variant is considered as pathogenic**

Jung, H., D. Lee, J. Lee, D. Park, Y. J. Kim, W. Y. Park, D. Hong, P. J. Park, and E. Lee. 2015. Intron retention is a widespread mechanism of tumor-suppressor inactivation. *Nat Genet* 47: 1242-1248

Carbonnier, V., B. Leroy, S. Rosenberg, and T. Soussi. 2020. Comprehensive assessment of TP53 loss of function using multiple combinatorial mutagenesis libraries. *Sci Rep* 10: 20368.

Mucaki, E. J., B. C. Shirley, and P. K. Rogan. 2020. Expression Changes Confirm Genomic Variants Predicted to Result in Allele-Specific, Alternative mRNA Splicing. *Front Genet* 11: 109.

Shirley, B. C., E. J. Mucaki, and P. K. Rogan. 2018. Pan-cancer repository of validated natural and cryptic mRNA splicing mutations. *F1000Res* 7: 1908.

**c.993G>C, c.993G>T (p.(Gln331His)) or c.993G>A (p.Gln331=)**

| **Occurrence in UMD** | **Occurrence in TCGA** | **Occurrence in GENIE** | **Occurrence in ICGC** | **Occurrence in MSKSCC** | **Occurrence in COSMIC** | **Occurrence in gnomAD** |
| --- | --- | --- | --- | --- | --- | --- |
| **85** | **6** | **8** | **3** | **6** | **21** | **0** |

Last base exon nucleotide 993 has been found to be mutated in various types of cancer with multiple nucleotides substitutions leading either to a synonymous variant (G>A) or to a conservative amino acid substitution (G>T and G>C)

The three variants have been predicted to impair splicing

The three variants have been shown to be associated with intron retention in various tumors

[https://validsplicemut.cytognomix.com](https://validsplicemut.cytognomix.com/)/

**This variant is considered as pathogenic**

Jung, H., D. Lee, J. Lee, D. Park, Y. J. Kim, W. Y. Park, D. Hong, P. J. Park, and E. Lee. 2015. Intron retention is a widespread mechanism of tumor-suppressor inactivation. *Nat Genet* 47: 1242-1248

Carbonnier, V., B. Leroy, S. Rosenberg, and T. Soussi. 2020. Comprehensive assessment of TP53 loss of function using multiple combinatorial mutagenesis libraries. *Sci Rep* 10: 20368.

Mucaki, E. J., B. C. Shirley, and P. K. Rogan. 2020. Expression Changes Confirm Genomic Variants Predicted to Result in Allele-Specific, Alternative mRNA Splicing. *Front Genet* 11: 109.

Shirley, B. C., E. J. Mucaki, and P. K. Rogan. 2018. Pan-cancer repository of validated natural and cryptic mRNA splicing mutations. *F1000Res* 7: 1908.

**c.375G>A or c.375G>T or c.375G>C p.(Thr125=)**

| **Occurrence in UMD** | **Occurrence in TCGA** | **Occurrence in GENIE** | **Occurrence in ICGC** | **Occurrence in MSKSCC** | **Occurrence in COSMIC** | **Occurrence in gnomAD** |
| --- | --- | --- | --- | --- | --- | --- |
| **291** | **0*** | **35** | **10** | **0*** | **49** | **0** |

* These databases do not report synonymous variants

**c.374C>T p.(Thr125Met)**

| **Occurrence in UMD** | **Occurrence in TCGA** | **Occurrence in GENIE** | **Occurrence in ICGC** | **Occurrence in MSKSCC** | **Occurrence in COSMIC** | **Occurrence in gnomAD** |
| --- | --- | --- | --- | --- | --- | --- |
| **124** | **5** | **21** | **4** | **6** | **33** | **3 10^-5^** |

Last base exon nucleotide 375 has been found to be mutated in various types of cancer with multiple nucleotides substitutions leading to a synonymous variant.

The three variants have been predicted to impair splicing

The three variants have been shown to be associated with intron retention in various tumors

<https://www.ncbi.nlm.nih.gov/clinvar/variation/177825/>

Varley JM, Chapman P, McGown G, Thorncroft M, White GR, Greaves MJ, Scott D, Spreadborough A, Tricker KJ, Birch JM, Evans DG, Reddel R, Camplejohn RS, Burn J, Boyle JM (1998) Genetic and functional studies of a germline TP53 splicing mutation in a Li-Fraumeni-like family. Oncogene 16:3291-3298

Varley JM, Attwooll C, White G, McGown G, Thorncroft M, Kelsey AM, Greaves M, Boyle J, Birch JM (2001) Characterization of germline TP53 splicing mutations and their genetic and functional analysis. Oncogene 20:2647-2654

| **c.541C>T** | **p.(Arg181Cys)** |
| --- | --- |

| **Occurrence in UMD** | **Occurrence in TCGA** | **Occurrence in GENIE** | **Occurrence in ICGC** | **Occurrence in MSKSCC** | **Occurrence in COSMIC** | **Occurrence in gnomAD** |
| --- | --- | --- | --- | --- | --- | --- |
| **154** | **2** | **17** | **5** | **16** | **40** | **0** |

Localised in the H1 helix of the P53 protein

Essential for DNA Binding Cooperativity of p53

Modulates the Decision between Cell-Cycle Arrest and Apoptosis

p.R181C is defective for apoptosis but not for growth arrest

p.R181C transactivate genes associated with growth arrest (p21WAF1) but is impaired for genes associated with apoptosis

TP53 R178C knockin mouse modeling the human TP53 R181C mutation show a modest but consistent increase in tumorigenesis and a decreased body fat content.

A founder mutation (p.Arg181Cys) has been detected in multiple families in the Middle East with inherited breast cancer. Due to the low penetrance of this mutation, homozygotes individuals (p.Arg181Cys/p.Arg181Cys) have been identified, a unique feature for TP53.

**This variant is considered as pathogenic**

Schlereth, K., R. Beinoraviciute-Kellner, M. K. Zeitlinger, A. C. Bretz, M. Sauer, J. P. Charles, F. Vogiatzi, E. Leich, B. Samans, M. Eilers, C. Kisker, A. Rosenwald, and T. Stiewe. 2010. DNA binding cooperativity of p53 modulates the decision between cell-cycle arrest and apoptosis. *Mol Cell* 38: 356-368.

Zick, A., L. Kadouri, S. Cohen, M. Frohlinger, T. Hamburger, N. Zvi, M. Plaser, E. Avital, S. Breuier, F. Elian, A. Salah, Y. Goldberg, and T. Peretz. 2017. Recurrent TP53 missense mutation in cancer patients of Arab descent. *Fam Cancer* 16: 295-301.

Lolas Hamameh, S., P. Renbaum, L. Kamal, D. Dweik, M. Salahat, T. Jaraysa, A. Abu Rayyan, S. Casadei, J. B. Mandell, S. Gulsuner, M. K. Lee, T. Walsh, M. C. King, E. Levy-Lahad, and M. Kanaan. 2017. Genomic analysis of inherited breast cancer among Palestinian women: Genetic heterogeneity and a founder mutation in TP53. *Int J Cancer* 141: 750-756.

Kang, J. G., C. U. Lago, J. E. Lee, J. H. Park, M. P. Donnelly, M. F. Starost, C. Liu, J. Kwon, A. C. Noguchi, K. Ge, P. Y. Wang, and P. M. Hwang. 2020. A Mouse Homolog of a Human TP53 Germline Mutation Reveals a Lipolytic Activity of p53. *Cell Rep* 30: 783-792.e5.

**c.1001G>T p.(Gly334Val)**

| **Occurrence in UMD** | **Occurrence in TCGA** | **Occurrence in GENIE** | **Occurrence in ICGC** | **Occurrence in MSKSCC** | **Occurrence in COSMIC** | **Occurrence in gnomAD** |
| --- | --- | --- | --- | --- | --- | --- |
| **86** | **5** | **16** | **3** | **4** | **21** | **0** |

The crystal structure of the p53-TD (residues 325–363) consists of a β strand and an α helix linked by the G334 residue. 61 The p53 tetramer is formed as a dimer of dimers with all four subunits geometrically equivalent. In vivo, the p53 dimerization occurs cotranslationally and the tetramerization occurs post-translationally.

Although, TP53 transcriptional activity of variants at codon 334 is not fully impaired, multiple structural analysis indicate that G334V variant forms amyloid aggregates compromising tetramer formation.

Forced overexpression of this variant in p53 null cells will not display a loss of activity using conventional assays such as transactivation or growth arrest.

Variants p.G334A, G334H or G334R have been also shown to have a mild effect on TP53 function.

Germline variant p.G334R has been associated with an increase risk of cancer in Ashkenazi Jews

**This variant is considered as pathogenic**

Powers, J., E. M. Pinto, T. Barnoud, J. C. Leung, T. Martynyuk, A. V. Kossenkov, A. H. Philips, H. Desai, R. Hausler, G. Kelly, A. N. Le, M. M. Li, S. P. MacFarland, L. C. Pyle, K. Zelley, K. L. Nathanson, S. M. Domchek, T. P. Slavin, J. N. Weitzel, J. E. Stopfer, J. E. Garber, V. Joseph, K. Offit, J. S. Dolinsky, S. Gutierrez, K. McGoldrick, F. J. Couch, B. Levin, M. C. Edelman, C. F. Levy, S. L. Spunt, R. W. Kriwacki, G. P. Zambetti, R. C. Ribeiro, M. E. Murphy, and K. N. Maxwell. 2020. A Rare TP53 Mutation Predominant in Ashkenazi Jews Confers Risk of Multiple Cancers. *Cancer Res* 80: 3732-3744.

Higashimoto, Y., Y. Asanomi, S. Takakusagi, M. S. Lewis, K. Uosaki, S. R. Durell, C. W. Anderson, E. Appella, and K. Sakaguchi. 2006. Unfolding, aggregation, and amyloid formation by the tetramerization domain from mutant p53 associated with lung cancer. *Biochemistry* 45: 1608-1619.

Kamada, R., T. Nomura, C. W. Anderson, and K. Sakaguchi. 2011. Cancer-associated p53 tetramerization domain mutants: quantitative analysis reveals a low threshold for tumor suppressor inactivation. *J Biol Chem* 286: 252-258.

Gordo, S., V. Martos, E. Santos, M. Menendez, C. Bo, E. Giralt, and J. de Mendoza. 2008. Stability and structural recovery of the tetramerization domain of p53-R337H mutant induced by a designed templating ligand. *Proc Natl Acad Sci U S A* 105: 16426-16431.

**c.1010G>TA p.(Arg337His)**

| **Occurrence in UMD** | **Occurrence in TCGA** | **Occurrence in GENIE** | **Occurrence in ICGC** | **Occurrence in MSKSCC** | **Occurrence in COSMIC** | **Occurrence in gnomAD** |
| --- | --- | --- | --- | --- | --- | --- |
| **62** | **5** | **16** | **3** | **4** | **21** | **1.1910^-5^** |

Although, TP53 transcriptional activity of variants at codon 337 is not fully impaired, multiple structural analysis indicate that R337H variant forms amyloid aggregates compromising tetramer formation.

Forced overexpression of this variant in p53 null cells will not display a loss of activity using conventional assays such as transactivation or growth arrest.

The founder mutation TP53 p.R337H is detected in 0.3% of the general population in southern Brazil. This mutation is associated with an increased risk of childhood adrenal cortical carcinoma (ACC) but is also common in Brazilian LFS/LFL families

Ribeiro, RC, F Sandrini, B Figueiredo, GP Zambetti, E Michalkiewicz, AR Lafferty, L DeLacerda, M Rabin, C Cadwell, G Sampaio, I Cat, CA Stratakis, and R Sandrini. “An Inherited P53 Mutation That Contributes in a Tissue-Specific Manner to Pediatric Adrenal Cortical Carcinoma.” *Proc Natl Acad Sci U S A* 98, no. 16 (2001): 9330–35.

Hainaut, P. “Tumor-Specific Mutations in P53: The Acid Test.” *Nat Med* 8, no. 1 (2002): 21–23.

Park, JH, J Li, MF Starost, C Liu, J Zhuang, J Chen, MI Achatz, JG Kang, PY Wang, SA Savage, and PM Hwang. “Mouse Homolog of the Human Tp53 R337h Mutation Reveals Its Role in Tumorigenesis.” *Cancer Res* 78, no. 18 (2018): 5375–83.

Gordo, S, V Martos, E Santos, M Menendez, C Bo, E Giralt, and J de Mendoza. “Stability and Structural Recovery of the Tetramerization Domain of P53-R337h Mutant Induced By a Designed Templating Ligand.” *Proc Natl Acad Sci U S A* 105, no. 43 (2008): 16426–31.

Lee, AS, C Galea, EL DiGiammarino, B Jun, G Murti, RC Ribeiro, G Zambetti, CP Schultz, and RW Kriwacki. “Reversible Amyloid Formation By the P53 Tetramerization Domain and a Cancer-Associated Mutant.” *J Mol Biol* 327, no. 3 (2003): 699–709.
